# The Effect of Tobacco Smoking on Musculoskeletal Health: A Systematic Review

**DOI:** 10.1155/2018/4184190

**Published:** 2018-07-11

**Authors:** Ahmad M. AL-Bashaireh, Linda G. Haddad, Michael Weaver, Debra Lynch Kelly, Xing Chengguo, Saunjoo Yoon

**Affiliations:** ^1^College of Nursing, University of Florida, Gainesville, FL, USA; ^2^College of Health and Human Services, University of North Carolina Wilmington, Wilmington, NC, USA; ^3^College of Pharmacy, University of Florida, Gainesville, FL, USA

## Abstract

This systematic review explored associations between smoking and health outcomes involving the musculoskeletal system. AMSTAR criteria were followed. A comprehensive search of PubMed, Web of Science, and Science Direct returned 243 articles meeting inclusion criteria. A majority of studies found smoking has negative effects on the musculoskeletal system. In research on bones, smoking was associated with lower BMD, increased fracture risk, periodontitis, alveolar bone loss, and dental implant failure. In research on joints, smoking was associated with increased joint disease activity, poor functional outcomes, and poor therapeutic response. There was also evidence of adverse effects on muscles, tendons, cartilage, and ligaments. There were few studies on the musculoskeletal health outcomes of secondhand smoke, smoking cessation, or other modes of smoking, such as waterpipes or electronic cigarettes. This review found evidence that suggests tobacco smoking has negative effects on the health outcomes of the musculoskeletal system. There is a need for further research to understand mechanisms of action for the effects of smoking on the musculoskeletal system and to increase awareness of healthcare providers and community members of the adverse effects of smoking on the musculoskeletal system.

## 1. Introduction

Tobacco smoke has more than 7,000 harmful chemical compounds that enter a human body either directly through smoking, indirectly through secondhand exposure to smoke exhaled by a smoker, or through downstream smoke released from a cigarette or pipe [1]. Both smokers and nonsmokers are at risk of exposure to the compounds of smoked tobacco that accumulate on the surfaces in a poorly ventilated environment; this method of exposure is known as thirdhand smoke exposure [2]. In the United States, there are approximately 500,000 annual deaths causally related to smoking and secondhand exposure to smoke [3].

Tobacco smoking has known adverse consequences on most human body systems. Researchers have focused more attention on the deleterious effects of smoking for high mortality diseases, such as cancer and diseases of the cardiovascular and respiratory systems, with less research attention on other body systems, such as the musculoskeletal system [3]. The musculoskeletal system is one of the largest human body systems, comprised of bones, joints, muscles, cartilage, tendons, ligaments, and other connective tissues [4]. An intact and functioning musculoskeletal (locomotor) system is necessary to perform activities of daily living and maintain quality of life [5, 6]. Several studies have investigated the association between smoking and musculoskeletal disorders. According to the recent Surgeon General report, the causal relationship between tobacco smoking and rheumatoid arthritis, periodontitis, and hip fractures has been confirmed [3]; however, there is inconclusive evidence to support causality between smoking and many other musculoskeletal disorders.

Searching online databases revealed significant growth in the body of literature investigating relationships between tobacco smoking and the musculoskeletal system. During our comprehensive online search, we did not encounter any systematic reviews examining those relationships; however, we did find 10 systematic reviews of the effects of tobacco smoking on components of the musculoskeletal system. Five systematic reviews focused on smoking and the effects on dental implants and found smoking increases the risk of peri-implant bone loss and implant failure [7–11]. Another systematic review revealed an association between smoking and lumbar disc herniation [12]. Three other reviews found smoking was related to negative postoperative outcomes on knee ligaments [13], higher complication rates after anterior cruciate ligament (ACL) reconstruction [14], and slowed healing of rotator cuff repair [15]. Also, one review found smoking was associated with rotator cuff tears and other shoulder symptoms [16]. Our review will be the first to collect and assess all the recent literature on the effects of smoking on the musculoskeletal system. This systematic review will orient scientists interested in the health effects of smoking about the state of the science over the last decade as they conduct more advanced research. Also, the amalgamation of these data in one document will be helpful to the research community as there is a high degree of similarity and shared characteristics between musculoskeletal system components.

This systematic review evaluated literature published in the last decade to summarize the evidence regarding the effect of smoking on the musculoskeletal system. This systematic review will answer two main questions: Is there an association between tobacco smoking and musculoskeletal health? What are the effects of tobacco smoking on the musculoskeletal health?

## 2. Methodology

This systematic review followed the criteria of A Measurement Tool to Assess Systematic Review (AMSTAR). Before the onset of the systematic review, a specific protocol was developed to minimize bias. This protocol included a priori research questions, a comprehensive literature search, inclusion criteria for studies, screening methods and reasons for exclusion, data abstraction, scientific study quality, data analysis, and synthesis.

A comprehensive literature search using PubMed, Web of Science, and Science Direct was conducted. This search covered 10 years from January 1, 2007, to March 18, 2017, and included only articles written in English. The search strategies included a combination of the following key words:* smoking, musculoskeletal system, bone, bones, joints, muscles, tendons, ligaments*, and* cartilage*. Medical Subject Headings (MeSH) were used during the search of PubMed. This step was helpful to expand the search; for example, the entry terms for MeSH of smoking were as follows:* smoking, cigar smoking, cigar, tobacco smoking, tobacco, hookah smoking, smoking, hookah, waterpipe smoking, waterpipe, pipe smoking, pipe, cigarette smoking,* and* cigarette*. All retrieved records were pulled from databases using EndNote X7. Duplicated records were removed via EndNote or manually when EndNote failed to recognize duplicates discovered by the authors during title/abstract reviews. After that, abstracts of the retained records were screened for inclusion criteria: English language, human subjects, published January 1, 2007–March 18, 2017, and investigating effects of smoking on the musculoskeletal system. Retained records then underwent full-text screening and records that did not meet the inclusion criteria or were editorials, commentaries, dissertations, case studies, or reviews (e.g., overview, systematic review, and meta-analysis) were excluded. A total of 243 final full-text articles were included in the review and used for data abstraction.

Based on an a priori protocol, data abstraction from selected full-text articles included citation (authors, year), study design, sample characteristics (size, age, sex, race/ethnicity, and type of sampling), study purpose, findings, comments, and/or limitations on data quality and validity. Two independent authors (first and second) extracted data using a standard form. The data abstraction process was piloted for the first 10 articles; it was successful and was used for the remaining articles. Any disagreements between authors were resolved through discussion.

The findings in this review were synthesized qualitatively as there was heterogeneity in study designs and populations. Our narrative analyses considered study design and quality.

## 3. Results

**Figure 1:**
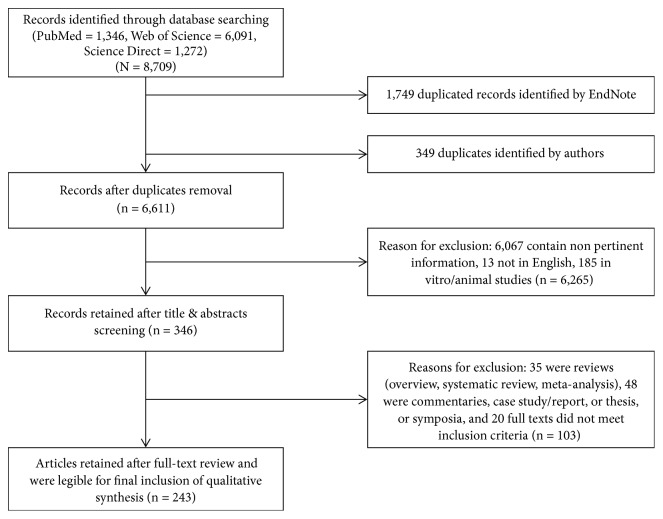
Process of literature search.

The comprehensive search of the literature identified 8,709 potentially relevant records; however, only 243 records met the inclusion criteria and underwent data abstraction and synthesis (Figure 1). The 243 articles were reviewed and the effects of tobacco smoking on musculoskeletal system were classified into 7 categories: (1) tobacco smoking and bones* (n* =132), which were subdivided into (a) bone mass: bone mineral density (BMD), bone mineral content (BMC), and bone turnover* (n* = 40); (b) fractures ( n = 16); (c) alveolar bone* (n* = 4); (d) periodontitis* (n* = 34); (e) implants* (n* = 33); and (f) grafts* (n* = 5); (2) tobacco smoking and joints* (n* = 54), which were divided into four subcategories: (a) rheumatoid arthritis (RA)* (n* = 29), (b) osteoarthritis (OA)* (n* = 14), (c) spondyloarthritis (SA)* (n* = 7), and (d) temporomandibular joint disorders* (n* = 4); (3) tobacco smoking and skeletal muscles* (n* = 20); (4) tobacco smoking and cartilage* (n* = 19), which were divided into two subcategories: (a) cartilage* (n* = 7) and (b) spinal cartilage* (n* = 12); (5) tobacco smoking and tendons* (n* = 6); (6) tobacco smoking and ligaments* (n* = 4); and (7) intrauterine and secondhand smoking effects on the musculoskeletal system* (n* = 8).

This review included studies using various designs: cohort studies* (n* = 106; 67 were prospective and 39 were retrospective), cross-sectional studies* (n* = 90), case-control studies* (n* = 16), randomized control trials (RCTs)* (n* = 14), and quasi-experimental studies* (n* = 10). Other study designs included secondary data analysis* (n* =5) and cross-sequential design* (n* = 2).  Table 1 presents the classification of study designs and related information based on the categories and subcategories.  Table 2 summarizes the effect of smoking on major outcomes of musculoskeletal health.  Table 3 provides comprehensive information on each study in the review.

### 3.1. Tobacco Smoking and Bones (*n*=132)

#### 3.1.1. Bone Mass: BMD, BMC, and Bone Turnover* (n* = 40)

Overall characteristics of these studies were as follows: 15 studies were conducted in males, 12 studies were conducted in females, and 13 included both sexes; 22 studies used data or samples from large-scale longitudinal studies; all studies used self-report to assess smoking habits, with the exception of 6 studies that used objective measures in addition to self-report: 3 assessed level of cotinine [24, 48, 49] and 3 assessed level of exhaled carbon monoxide (EXCO) [22, 36, 41].  Table 3 provides comprehensive details on the findings from those studies for effects of smoking on selected bone-related outcomes.

According to a majority of studies, smoking had adverse effects on BMD across age categories and sex. In males, regardless of age, method, and site of measurement for bone density, the cross-sectional studies found smokers had significantly lower BMD than nonsmokers [27, 42–44, 46, 52, 54, 55]. The cohort studies found male smokers exhibited a significant decline in BMD [32, 33, 50, 54]. There was only one cross-sectional study that reported no significant difference in calcaneus BMD between 3 groups: an alcohol drinking-only group, combined alcohol drinking and smoking group, and control nondrinking/nonsmoking [39]. In adolescent females, 2 cross-sectional studies found a high frequency of smoking was associated with lower rate of total body BMC [29] and hip BMD [28, 29]; these findings were supported by cohort studies that found initiation of smoking at age 13 affected bone accrual and was associated with low mean BMD at age 17 [33, 47]. Another cross-sectional study of adolescent females reported significant linear relationships between urinary cotinine and BMD of the femoral neck, total femur, and lumbar spine [48]. However, only one cross-sectional study in adolescent females found no significant difference in BMC and BMD between smokers and nonsmokers [30]. In premenopausal women, one cross-sectional study reported the BMD of smokers was not significantly different than the BMD of nonsmokers [20].

In postmenopausal women, cross-sectional study findings demonstrated postmenopausal women who smoked had significantly lower BMD than postmenopausal women who did not smoke [23, 56] and an increased risk of falls regardless of the BMD T-score [21]. Two randomized control studies were conducted in postmenopausal women. One study found consumption of blackberries was effective in reducing bone loss of the total body BMD in a smokers' group* (P* = 0.0284) [38]. Another study found quitting smoking significantly associated with increased body weight, fat, muscles, and functional mass that affected BMD [41]. A study using data from one RCT found administration of nasal estradiol for 2 years increased the lumbar spine BMD of smokers* (P* = 0.03) but did not increase total hip BMD* (P* = 0.89)[18]. Finally, two cross-sectional studies enrolled both males and females, and one reported BMD and BMC were significantly lower in smokers than those of nonsmokers [26]; the second study used a small sample and found no association between pack-years and BMD [31].

Biological mechanisms were examined in several studies, most of which were cross-sectional. A comparative analysis of smokers to nonsmokers found smokers had a higher receptor activator of nuclear factor-B ligand (RANKL-positive) CD4 (+) and CD8 (+) T cells (All* P *< 0.001) [25], a lower periosteal gene expression of bone morphogenetic proteins (BMP-2, BMP-4, and BMP-6) [24], and a lower mean concentration of bone marrow progenitor cells (BMPCs)* (P* = 0.004) [17]. Both BMP and BMPCs are required for musculoskeletal healing and regeneration. Smokers also had lower antioxidant enzymes (superoxide dismutase, glutathione peroxidase, and paraoxonase), higher levels of oxidative stress products (malondialdehyde, nitric oxide)* (P* < 0.001) [23], lower levels of parathyroid hormone (PTH) [34, 36], vitamin D [26, 36, 37], biomarkers of serum osteocalcin, and 24-hour urinary excretion of calcium [34].

The correlational analysis did not find a significant effect for serum osteocalcin (OC) or tartrate resistant acid phosphatase isoenzyme 5b (TRACP 5b) [55]. The interaction between smoking and other compounds found smoking with calcium intake did not reach statistical significance for BMD [19]; however, elderly women with the lowest tertile of choline who were exposed to nicotine had the highest risk for low BMD (OR=4.56, 95%CI: 1.87-11.11) [49]. One prospective study found growth hormone (GH) therapy after 3-5 years did not significantly improve total BMC of smokers* (P* = 0.09) [40].

The interaction between smoking and genetic factors was also investigated. A potential interaction was reported between smoking and receptor-related Protein 5 (LRP5) C135242T (rs545382) on osteoporosis in postmenopausal women [35] and between smoking and polymorphism of glutathione S-transferases (GSTT1) on bone quality index in young adult men [45]. One cross-sectional study reported a relationship between PTH and hip BMD only for nonsmokers [53]. Finally, in a cross-sectional methodological study, a strong correlation between bioelectrical impedance analysis (sBIA) and dual energy X-ray absorptiometry (DEXA)] regarding whole-body fat mass (FM) and lean mass (LM) (*r* > 0.9,* p* < 0.001) was found [51].

#### 3.1.2. Fracture* (n* = 16)

The overall characteristics of these studies were as follows: 12 studies were conducted in both sexes: 2 studies in females only [61, 71] and 2 studies in males only [62, 70]; 12 studies were cohort studies; 3 obtained data or samples from large-scale longitudinal studies; all studies used self-report to assess smoking habits with the exception of one study that assessed levels of cotinine [67].  Table 3 provides comprehensive details on studies which examined the prevalence of fracture in smokers, the association between smoking and fracture risk, fracture healing, the biological mechanism of fracture in smokers, and the interaction of smoking and other fracture risks.

Fracture was more prevalent in male smokers than in the males who never smoked* (P* < 0.05) [70]. Smoking was also found to increase the likelihood of fracture. In one cohort study of elderly men, current smoking increased the risk of all new [hazard ratio (HR) = 1.76, 95% Cl: 1.19-2.61] and osteoporotic fractures (HR = 2.14, 95%Cl: 1.18-3.88) [62]. Similar findings were reported in elderly women; former and current smokers compared to nonsmokers had increased risk for fracture [59, 71], including nonvertebral fractures in patients with diabetes (OR = 3.47, 95%CI: 1.82-6.62,* P* = 0.001) [61]. Regardless of fracture site, six cohort studies and one case-control study examining both sexes found smoking was significantly associated with poor fracture outcomes, such as nonunion (*P* < 0.01) [57, 58, 60, 66], lower trabecular strength (beta = -0.323;* P* = 0.045) and toughness (beta = -0.403;* P* = 0.018) [68], and delayed mean healing time [64]. No significant association was found between fracture and delay in filling [69] work absenteeism* (P* = 0.1177) [66] or low mental and physical-function scores on the Short Form 36 (SF-36) [66].

Two prospective cohort studies of both sexes investigating the postsurgery level of serum transforming growth factor-beta 1 (TGF-beta 1) found TGF-beta 1 was lower in smokers than in nonsmokers at 4 weeks [65] and 8 weeks [63]. The trend of lower level of TGF-beta 1 in smokers than that of nonsmokers was observed in both groups of patients with normally healed fractures and delayed healed fractures [65]. Finally, two studies examined interactional effect of smoking and other factors. In the first study, changes in BMI had an effect on fracture risk in nonsmokers, but not in smokers [72]. In the second study, plasma dimethylglycine (DMG) increased the risk of hip fracture in cohort of elderly males and females (HR = 1.70, 95 % CI: 1.28-2.26), and such risks were noticeably increased in women exposed to nicotine (HR = 3.41, 95%CI: 1.40-8.28) [67].

#### 3.1.3. Alveolar Bone* (n* = 4)

In two cross-sectional studies, one study found smokers had significantly lower alveolar bone density values* (P* ≤ 0.002) and a greater distance from cemento-enamel-junction to the alveolar bone crest* (P* < 0.0001) [74]. The second study did not find smoking significantly correlated with alveolar crest height loss [75]. The third cross-sectional study found smoking negatively affected the expression of bone sialoprotein (BSP) and osteocalcin (OC) mRNA* (P*< .05) and positively altered the expression of Type I collagen (COL-I)* (P*< .05); however, smoking was not statistically correlated with the expression of mRNA for tumor necrosis factor-alpha (TNF-*α*), transforming growth factor-beta (TGF-*β*), or osteoprotegerin (OPG) [73]. The fourth study, an RCT, found that, despite involvement of smokers in dental hygiene program, smokers had significantly lower density of alveolar bone by Day 365 (*P* < 0.05) and Day 545 of follow-up during the dental hygiene program (*P* < 0.01) [76].  Table 3 provides comprehensive details on the 4 studies that examined the effect of smoking on the alveolar bone.

#### 3.1.4. Periodontitis* (n*=34)

Thirty-four studies examined the prevalence and clinical parameters of periodontitis in smokers, the biological mechanism of smoking, the interaction between smoking and periodontitis risk factors, and interventions to minimize periodontitis in smokers. The characteristics of these studies were as follows: 27 studies were conducted in both sexes; 7 studied males only. Twenty-four studies were cross-sectional, 5 studies were RCTs, 4 studies were cohort, and 1 study was a case-control. Two studies out of 34 obtained data or samples from large-scale longitudinal studies. All the studies used self-report to assess smoking, with the exception of three studies that assessed levels of cotinine [88, 99, 101].  Table 3 provides comprehensive details on the studies that examined the relationship between smoking and periodontitis.

Eleven studies investigated the prevalence and association of smoking on periodontitis and periodontal parameters. Ten studies were cross-sectional. The comparative analysis found smokers compared to nonsmokers had significantly deeper periodontal pockets [77, 89, 90, 92, 108], higher mean clinical attachment loss (CAL) [78, 91, 92, 108], higher mean plaque scores [78, 91, 92], greater fraction of teeth with apical periodontitis [79], higher marginal bone loss, and a greater number of missing teeth [92]. In a correlational analysis, smoking was strongly associated with alveolar bone loss [84] and the percentage of palatal periodontal pockets ≥ 6 mm [77]. Heavy smoking was also associated with higher prevalence [79, 94] and severity of periodontitis [77, 94]. A comparative study between different modalities of smoking found cigarette smokers had higher frequency of probing pocket depth ≥ 4 mm and a higher incidence of severe periodontitis compared to nontobacco users [90]. Two studies compared cigarettes smokers to waterpipe and narghile users and found a similarity between groups on most periodontal parameters [92, 95].

Fourteen studies examined the potential biological mechanism for smoking in periodontitis and what potential biomarkers may be affected. Thirteen studies were cross-sectional designs and enrolled both sexes. Three studies examined smoking and nonsmoking subjects without periodontitis and found smokers had significantly higher synthesis of lipoxygenases and isoprostanes in the extracted periapical granuloma [83], higher whole salivary IL-1 beta and IL-6* (P* < 0.05) [93] but a lower total amount of platelet-derived growth factor (PDGF-AB)* (P* = 0.014) in gingival crevicular fluid [85]. Four studies were conducted in smokers and nonsmokers with periodontitis and found smokers had significantly lower levels of salivary osteocalcin (OC)* (P* < 0.001) [88]; a lower median serum level of OPG* (P* = 0.0006) [88]; higher levels of prostaglandin E-2, lactoferrin, albumin, aspartate aminotransferase, lactate dehydrogenase, and alkaline phosphatase [96]. The groups had similar levels of salivary C-telopeptide pyridinoline cross-links of Type I collagen* (P* > 0.05) [88]; median serum receptor activator of nuclear factor kappa-B ligand (RANKL)* (P* = 0.0942) [99]; and gingival crevicular levels of RANKL and osteoprotegerin (OPG) [107] and similar proportion of identified pathogens [96]. Seven studies enrolled both smokers and nonsmokers in groups with or without periodontitis. Based on periodontal status, the group with periodontitis (smokers and nonsmokers) had significantly higher plasma sRANKL, TNF, a proliferation-inducing ligand (APRIL and BAFF) and lower OPG* (P* < 0.01) [101], and higher salivary OC* (P* < 0.05) [102] than the healthy control group (smokers and nonsmokers without periodontitis). Interestingly, these two studies found levels for some of these markers were altered by smoking; sRANKL and TNF concentrations were significantly greater (*P* = 0.011,* P* = 0.001; respectively), and OPG concentration was significantly lower (*P* = 0.001) in smokers with periodontitis; however, such trend was not seen for salivary OC [101, 102]. The results from these studies indicated that smokers had more lymphocyte and higher levels of both IFN-*γ* and IL-13, regardless of periodontal status [82], had higher salivary sCD44 profiles* (P* < 0.001) with the highest levels recorded in smokers in the periodontitis group [87], and had significantly higher levels of salivary calcium level* (P*< 0.05) [97]. A subgroup analysis for smoking and periodontal status found that smokers with chronic periodontitis exhibited significantly higher levels of sIgA [98] and lower plasma OPG concentrations* (P* = 0.007) but higher sRANKL/OPG ratio* (P* = 0.01) than smokers without periodontitis [103]; however, smokers and nonsmokers with periodontitis exhibited similar values for plasma sIgA, sRANKL, and OPG concentrations. Smoking is one of the greatest risks for periodontitis and may increase host susceptibility to tissue destruction especially in presence of other factors such as the functional defect of leukocyte and monocyte [98]. These findings indicate periodontal inflammation in smoker with chronic periodontitis patients, as evidenced by high levels of sIgA, seems to lower plasma OPG levels and thereby increase the RANKL/OPG ratio and possibly play a role in the increased susceptibility for alveolar bone destruction in smoker subjects.

One cross-sectional study suggested the interaction between smoking and vitamin D receptor gene polymorphism (CC+CT genotypes of FokI) increased the risk of periodontitis (OR = 9.6, 95%CI: 4.5- 20.4). The combined effect was 3.7 times greater than expected from the sum of individual effects [80]. Eight interventional studies examined therapies to manage periodontitis. One observational cohort study that monitored the effect of smoking cessation found quitters had a higher reduction of mean probing depth and CAL relative to nonquitters* (P* ≤ 0.05) [105]. Another prospective observational study found periodontal maintenance therapy every 3-4 months inhibited the progression of CAL, probing depth, and tooth loss in smokers [86]. Wan et al. [109] found in a prospective cohort study that anterior teeth, sites without plaque, and nonsmoking were significantly associated with a greater reduction in probing pocket depth [109]. Three RCTs found adjunct treatments of low-dose doxycycline for 6 months [100], systemic azithromycin [81], or a daily dose of 325 mg of aspirin [106] did not significantly improve periodontal parameters in smokers with chronic periodontitis. In contrast, 2 RCTs in smokers with chronic periodontitis successfully improved some periodontal parameters. Compared with treatment using only scaling and root planing (SRP), the treatment using Simvastatin (1.2% biodegradable controlled-release gel) as an adjunct to scaling and root planing (SRP) significantly reduced probing depth and significantly increased bone filling (all* P* < 0.001) [104]. Smoking is one of the greatest risks for periodontitis and is associated with poor periodontal parameters; such finding provides evidence that the treatment used Simvastatin besides SRP in smokers suffering from chronic periodontitis was more effective in reducing the negative effect of smoking on the periodontal parameter than the treatment using only SRP. The second RCT found the treatment using modified YJ (mYJ) Chinese medicinal herbs in a nonsurgical treatment for smokers suffering from periodontitis was associated with higher computer-assisted densitometry values than the treatment using original YJ Chinese medicinal herbs with nonsurgical treatments* (P* = 0.025) [110]. Also, this finding provides evidence that the use of mYJ Chinese medicinal herbs in a nonsurgical treatment was effective in reducing the negative effect of smoking on the periodontal parameter as evidenced by the increases in radiographic alveolar bone density.

#### 3.1.5. Bone Implants* (n* = 33)

There were 33 studies which investigated implant survival/failure rates, clinical parameters of success/failure, risk factors of implant survival, interaction between smoking and risk factors on the implant survival, effects of implants on surrounding tissue, complications associated with implants, biological mechanisms of smoking effect on implants, and interventions to reduce the effects of smoking and enhance implant survival rate in smokers. The characteristics of these studies were as follows: all 33 studies examined dental implants and enrolled both sexes; 19 were cohort studies; 7 were cross-sectional studies; 4 were RCTs; and 3 were case-control studies. Eleven studies had small samples. All studies used self-report to assess smoking.  Table 3 provides comprehensive detail on these studies exploring the effects of smoking on bone implants.

Thirteen studies examined the effect of smoking on dental implant survival with special consideration of implant type and time of follow-up. Eleven studies were cohort studies. Two studies investigated smoking and early implant failure and the first study found early implant failure was threefold higher in smokers than nonsmokers [141] while the second study found frequency of tobacco smoking was not associated with early implant failure [136]. Ten studies examined long-term survival/failure of dental implants. Two studies reported smoking did not influence implant survival rates [115, 123], although 8 studies provided contradictory findings. A correlation analysis found smoking status [112, 139], and pack-years [139] were inversely associated with dental implant survival. A comparative analysis between smokers and nonsmokers found smokers had lower implant survival rates [114, 119, 124, 130, 132, 142]. A subgroup analysis based on implant type found smokers had higher failure rates for turned [132] and smooth-surface implants [114]. One study of only tobacco smokers found implant survival with turned or screw surfaces was similar in tobacco smokers regardless of periodontal status [111]. Five of 13 studies measured marginal bone loss; 4 reported smokers demonstrated significantly greater marginal bone loss than nonsmokers [119, 123, 141, 142], and two studies did not report a significant difference [130, 131].

Six studies examined the clinical effects of implants on surrounding tissue in smokers. One retrospective cohort study found smoking was associated with overall complications (e.g., implant loss, infection, peri-implantitis, and mucositis) (P = 0.008) [129]. Five studies examined histometric parameters for dental mini-implants, and one case-control study carried out on smokers found mean bone-to-implant contact (BIC%) better in sandblasted acid-etched surfaces than machined surfaces (22.19 ± 14.68% versus 10.40 ± 14.16%, P < 0.001) [117]. Smoking is associated with increased risk of bone implant failure due to its negative effect on tissues surrounding the implant; such finding indicates that the negative effect of smoking on histometric measurements after dental mini-implants was significantly minimized through using of implants with sandblasted acid-etched but it was not improved with the use of implants with machined surfaces. The remaining 4 studies were prospective cohort studies and found smokers had significantly lower BIC% [120, 133], lower bone density in thread areas (BA%) [133], less stability at 3, 4, 6, and 8 weeks after surgery [135], and less regrowth of papillae and midfacial soft tissue [128].

Eight studies examined the biological mechanism of smoking on tissue surrounding dental implants and explored potential biomarkers that could be affected by this mechanism. Five of eight were cross-sectional studies; one study analyzed peri-implants fluid of smokers' prior implant placements and found smoking negatively altered the mRNA expression of bone sialoprotein (BSP) and osteocalcin (OC) and positively affected the expression of Type I collagen (COL-I)* (P* < 0.05). However, smoking was not correlated with the expression of TNF-*α*, transforming growth factor-beat (TGF-*β*), or OPG* (P* > 0.05) [73]. Four studies analyzed peri-implant fluid and found smokers and nonsmokers had similar levels of pathogens [113, 140], OPG, and RANKL/OPG [126]. However, there were contradictory findings regarding the level of cytokines (IL-4, IL-8, or TNF- *α*) in smokers and nonsmokers, as they were reported to be similar in one study [113], significantly lower in one study [126], and significantly higher in yet another study [137]. One case-control study found heavy smokers with an IL-1 polymorphism did not increase their risk for peri-implantitis [121]. One short-term prospective study found a 7-day follow-up for the whole genome array of implant adherent cells was not different between smokers and nonsmokers [138]. The long-term prospective cohort study found smokers with previous periodontal disease had significant clinical signs of inflammation and significantly higher counts of pathogenic bacteria [127].

Four studies were randomized control trials; 2 measured peri-implant parameters for implants with different configurations. One trial found smoking did not influence peri-implant soft tissue response (recession and the papilla index) [116]; a second study found smoking doubled marginal bone loss regardless of treatment [134]. One methodological RCT using stereolithographic surgical guides found smoking was associated with inaccurate implant placement [118]. A therapeutic RCT found mechanical debridement with adjunct antimicrobial did not significantly improve parameters of bleeding on probing, probing depth, or crestal bone loss in smokers [122]. There were positive outcomes (high implant survival, bone level, and low rate of biological complications) reported by one retrospective cohort study where the authors monitored dental implant rehabilitation in patients with systemic disorders and smoking habits [125].

#### 3.1.6. Bone Graft* (n* = 5)

Two of the 5 studies were randomized clinical trials. The first trial found smokers who received an acellular dermal matrix graft (ADMG) with enamel matrix derivative (EMD) had a higher mean gain in recession height and root coverage than smokers who received ADMG alone [143]. This finding indicates that the treatment combining EMD with ADMG was found to be more effective in reducing the negative effect of smoking on root coverage than the treatment using only ADMG. A second trial found regenerative treatment of platelet-rich plasma combined with a bovine-derived xenograft did not improve periodontal parameters in smokers [147]. Three long-term prospective cohorts compared smokers to nonsmokers and found smokers had significantly higher marginal bone loss up to 4 years after onlay bone grafting in the atrophic maxilla [145]. These patients also had higher tissue inflammation around augmentation sites once they received bone graft titanium- reinforced ePTFE membranes [144] and had similar survival rates for dental implants after A Le Fort I osteotomy and interpositional bone graft in combination with implants in the atrophic maxilla [146].  Table 3 provides comprehensive details on the 5 studies that examined the effects of smoking on bone graft.

### 3.2. Tobacco Smoking and Joints* (n* =* 54)*

#### 3.2.1. Rheumatoid Arthritis* (n* = 29)

The overall characteristics for these 29 studies were as follows: all studies enrolled males and females, 17studies were cohort studies, 7 were cross-sectional studies, 3 were case-control studies, 1 was an RCT, and 1 was a secondary analysis. Twelve studies obtained data or samples from large-scale longitudinal studies and all used self-report to assess smoking habits with an exception of one study that assessed level of cotinine [159].  Table 3 provides comprehensive detail about studies that examined the effect of smoking on several outcomes in patients with RA.

Five studies of varying design (2 cross-sectional studies, 2 prospective cohort studies, and 1 case-control study) enrolled patients from both sexes of similar age. These studies examined the effect of smoking on RA clinical outcomes, such as disease activity, functional capacity, radiographic damage, serology, and existence of extraarticular manifestations. Overall, the collective results were that smokers had significantly higher scores on the Disease Activity Score of 28 joints (DAS 28) [162, 167], the functional disability score (Health Assessment Questionnaire) [162], the simple erosion narrowing score [167], CRP [162], and a rheumatoid factor titer [167]. These patients demonstrated severe extraarticular RA [162] and took significantly more disease-modifying antirheumatic drugs (DMARD) [176]. One study reported no difference in DAS28 and radiographic scores between smokers and nonsmokers [176]. Smoking was found to be independently associated with DAS28-CRP3 in human leukocyte antigen-shared epitope (HLA-SE-positive) patients, but not in HLA-SE-negative patients* (P* for interaction = 0.02) [158], higher Modified Health Assessment Questionnaire [158] scores, and greater number of rheumatoid nodules [161]. Smoking and RA remission were investigated in two studies. A cross-sectional study reported current smokers had higher remission rates than persons who had never smoked or former smokers [154]; however, a prospective cohort study reported lower remission rates in current smokers compared to persons who had never smoked or former smokers at 12-month follow-up [169]. Two prospective studies examined smoking on RA progression, and one study with a large sample size reported radiographic progression for joint damage was not significantly different between smokers and nonsmokers (*P* = 0.26), but further analysis by authors found smoking intensity (pack-day) to be inversely associated with radiographic progression [152]. Meanwhile, a second study with a small sample size found current smoking associated with radiographic progression [165].

There were 10 studies that examined the effect of smoking on response to RA therapies. A first group of 5 cohort studies was conducted in patients with early stage RA. Four studies found smoking associated with poor response after 3 months of methotrexate or anti-TNF-*α* therapy [166], after 6 months of combined therapy of methotrexate and sulfasalazine [163], and after 12 months of glucocorticoids and DMARDs [169, 170]. One study reported no difference in response to therapy after 12 months' follow-up in patients who continued or quit smoking [175]. Similar findings were reported in 5 studies that enrolled RA patients regardless of disease stage. Four studies reported current smoking was associated with poor response after 3 months [148, 160, 171] and 12 months [160] of anti-TNF-*α* therapy and after 48 and 102 weeks of therapy that included methotrexate [159]. One of the 5 studies reported exposure to secondhand smoking did not influence the response to RA therapy after 3, 6, and 12 months and 2 years [168].

Seven studies (2 case-control studies, 3 cohort studies, 1 cross-sectional study, and 1 secondary analysis) investigated the interaction between smoking and other factors on RA. The results of these studies found a significant increase in disease activity when there was an interaction between heavy smoking and HLA-DR beta 1 4-amino acid haplotype primarily Positions 11 and 13 [155], between smoking and all positive anti-citrullinated peptide antibodies (ACPA) [153, 157, 164], and between ever smoking and mannose-binding lectin (MBL2) genotype YA/YA [156]. Further results were increased signs of joint inflammation in first-degree relatives who were younger than 50 and had smoked more than 10 pack-years [172]. There was no interaction found between smoking and endothelial growth factor A haplotype [VEGFA-2578 AA genotype and (A_2578-C_460-G+405)], but endothelial growth factor A haplotype was found to be associated with reduced disease activity in patients of RA who had never smoked [151]. Four studies investigated smoking effects on certain mechanisms and biological markers in patients with RA. One prospective cohort study found smoking and ACPA predicted persistence of high levels of survivin (OR = 4.36, 95% CI: 2.64-7.20, P < 0.001, positive predictive value 0.66, and specificity 0.83) [174]. Two cross-sectional studies compared level of essential and trace elements of smoker and nonsmokers with RA and matched healthy controls of smokers and nonsmokers to determine if there were any associations between toxic elements, cigarette smoking, deficiency of essential trace elements, and risk of arthritis. One study found smokers and nonsmokers RA patients had significantly higher hair levels of toxic elements (Cd and Pb) and lower hair levels of trace elements (Zn, Cu, and Mn) than those of smokers and nonsmokers healthy individuals [149]. The second study found smokers with RA had significantly higher hair and blood levels of toxic element (Cd, Pb, Hg, and AS) and lower hair and blood levels of trace elements (Zn, Cu, Mn, and Se) [150]. Finally, another cross-sectional study reported smoking pack-years was inversely correlated to body fat composition in patients with RA [173].

#### 3.2.2. Osteoarthritis (*n* = 14)

Three cross-sectional studies provided disparate findings on the effects of smoking and OA. One study reported smoking was not significantly associated with hand OA in a Chuvashian community [182]; two other studies reported an inverse relationship between smoking and radiographic knee OA* (P* = 0.019) [190] and between indirect smoking and knee and hip OA (OR = 0.271; 95% CI: 0.088-0.828) [183]. One prospective study reported smoking was not significantly associated with the prevalence or incidence of radiographic knee OA [188]. Three prospective studies found smoking associated with higher pain scores [177, 178], increased risk for cartilage loss at the medial tibiofemoral joint (OR = 2.3, 95% CI:1.0 - 5.4), and increased risk for cartilage loss at the patellofemoral joint (OR = 2.5, 95% CI: 1.1 - 5.7) [177]; however, smoking reduced the risk of total joint replacement (TJR) in presence of [rs1051730 T] alleles (HR = 0.84, 95% CI: 0.76 – 0.98, per T allele) [181]. Two prospective studies found smoking significantly associated with higher complication rates [189], but not with functional outcomes after a tibial osteotomy in patient with RA [180]. There were five studies that examined the association of smoking with the risk for joint replacement and the risk for complications after joint surgery. Two cohort studies investigated the risks for joint surgery: one prospective cohort study reported smoking increased the risk for total joint replacement (TJR) in males [186]; however, conflicting evidence was reported by another retrospective cohort study that found an inverse association between smoking and TJR (adjusted-HRs: 0.60; 95% CI: 0.48-0.75, and 0.70; 95% CI: 0.56-0.86 in men and women, respectively), but this study investigated the risk for only primary TJR and included both sexes [187]. Another prospective study of patients who underwent total hip or knee arthroplasty found no difference in perioperative mortality rates between smokers and nonsmokers; however, smokers had a higher complication rate [179]. In two retrospective studies, smoking significantly increased the risk for early failure of total hip arthroplasty [185] and wound breakdown after total ankle replacements [184].  Table 3 provides comprehensive details on the 14 studies in this subsection.

#### 3.2.3. Spondyloarthritis* (n* = 7).

Three of these seven studies investigated the effects of smoking on biological markers in patients with SA; 5 studies investigated effect of smoking on clinical, functional, and imaging outcomes of SA. Three cross-sectional studies found smoking was associated with lower matrix metalloproteinase-generated Type II collagen fragment in patients with SA* (P* = 0.02) [193] and higher level of vascular endothelial growth factor in patients with ankylosing spondylitis (VEGF)* (P* < 0.05) [195, 196]. Three cross-sectional studies, two in patients with ankylosing spondylitis and one in patients with early axial spondyloarthritis, and one prospective cohort study in patients with early axial spondyloarthritis reported smoking was associated with higher pain scores [191, 192], disease activity and functional status [191, 192, 195], poor quality of life [191, 192], and spinal radiographic/MRI progression [191, 194]. Also, pack-years were positively correlated with duration of inflammatory back pain (*r =* 0.628,* P* < 0.001), Bath AS Functional Index (BASFI) (*r =* 0.443,* P* < 0.001), and the severity of radiographic damage assessed by the modified Stroke AS Spine Score (mSASSS) (r = 0.683,* P* < 0.001) [195]. Finally, one case-control study found collagen IX tryptophan (Trp+2) alleles and smoking status did not influence the risk for cervical spondylotic myelopathy (OR = 1.34, 95% CI = 0.85-2.18,* P* > 0.05); however, smoking intensity with collagen IX tryptophan (Trp+2) exhibited a dose-response relationship with cervical spondylotic myelopathy [197].  Table 3 provides comprehensive details about the 7 studies that examined the effects of smoking on SA.

#### 3.2.4. Temporomandibular Joint Disorders* (n* = 4)

Of the four studies in this subsection, 4 studies with varying designs compared smokers to nonsmokers and found smokers had higher temporomandibular joint disorder (TMD) pain intensity [198–201]. Further analysis of these studies found no differences in pain intensity between smokers and nonsmokers after adjustment for demographic variables [201]. The number of cigarettes was associated with pain intensity only in females [198] and females younger than 30 were more likely to develop TMD symptoms than females over the age of 30 [200].  Table 3 provides comprehensive details on the four studies in this subsection.

### 3.3. Tobacco Smoking and Skeletal Muscles (n = 20)

Compared to bones and joints, few studies investigated the effect of tobacco smoking on skeletal muscles. The overall characteristics of these 20 studies were as follows: 11 studies enrolled both sexes, 8 enrolled only males, and 1 enrolled only females, 10 studies were quasi-experimental, 5 were cross-sectional studies, 4 were cohort studies, and 1 was a case-control study, and all studies used self-report to assess smoking habits.  Table 3 provides comprehensive details on these studies which examined the effects of smoking on the anatomical, biological, metabolic, physical, and functional outcomes of skeletal muscles.

Two studies investigated the association between smoking and the anatomy of skeletal muscles. A cross-sectional study reported smokers had lower Types I and IIa muscle fibers than nonsmokers indicating smokers' skeletal muscles had oxidative fiber atrophy [204]. The prospective cohort study of only males reported rectus femoris volume (RFVOL) at baseline (prior training) was lesser in smokers than in nonsmokers, although RFVOL was significantly increased with training, and due to those authors suggested that training reversed the effects of smoking [214]. Three quasi-experimental studies investigated the biological effects of smoking. The results of those studies reported smokers had decreased local muscle O2Hb [206], thiobarbituric acid [211], and catalase [211] levels, an increase in inflammatory markers (sTNFR1) [211] and similar VO2 [206, 211], lactate [206], superoxide dismutase (SOD) [211], and succinate dehydrogenase (SDH) activity [220], inflammatory cytokines (IL-6, IL-10, and sTNFR2), myoglobin concentration [220], and capillarization [220] during leg muscle exercises.

Twelve studies examined the effects of smoking on physical and functional properties of skeletal muscle: 7 studies examined muscle strength, 2 studies examined muscle thickness, and 3 focused on maximal voluntary contraction. The findings of the 3 pretest/posttest, 2 prospective cohort, and 2 cross-sectional studies on muscle strength were that smoking was significantly associated with a reduction in back extensor muscle strength [202, 208], grip strength [203, 215], and knee muscle strength [207]. One prospective cohort study reported that parameters of body composition and muscle strength were increased in subjects who quit smoking compared to subjects who continued smoking [218]. One study reported an inverse correlation between pack-years and muscle strength [217]. Two pretest/posttest studies were not congruent in terms of findings regarding percentage of change in muscle thickness (PCMT) and relative contribution ratio (RCR) of both internal oblique (IO) and transversus abdominis (TrA) muscles [205, 209]. The first study reported PCMT and RCR were not significantly different between smokers and nonsmokers [209]; however, the second study reported significant differences between smokers and nonsmokers in regard to PCMT of the TrA and in RCR of both TrA and IO [205]. Three pretest/posttest studies of male smokers and nonsmokers had similar findings regarding the maximal voluntary contraction for quadriceps muscles [210, 221], rectus abdominis, and external oblique [216]; however, maximal voluntary contraction was significantly higher in smokers than in nonsmokers [216].

Three studies (case-control, cross-sectional, and prospective cohort) investigated the interaction of smoking and obstructive lung disease on skeletal muscles. Those studies found smoking in the presence of obstructive lung disease to be significantly associated with increased muscle injury [212] and lower weight and lean mass [219]; however, smoking, regardless of patient spirometry status, was the only independent variable associated with lower quadriceps Klotho levels [213].

### 3.4. Tobacco Smoking and Cartilage (n = 19)

#### 3.4.1. Knee Joint Cartilage* (n* = 7)

Four studies (2 prospective cohorts, 1 case-control, and 1 cross-sectional) described the effect of smoking on knee joint cartilage. A cross-sectional analysis of these studies found both smoking and pack-years were positively associated with the volume of tibia cartilage [228] and femoral medial, intercondylar, and lateral cartilage [226]. There was an inverse association between smoking and cartilage strain ratio [226].There was no consensus regarding the risk of tibiofemoral cartilage defects; one study reported smokers experienced a higher risk for medial and lateral tibiofemoral cartilage defect (OR: 4.91,* P* < 0.05), and such risk was increased with pack-years (OR 9.90 and OR 12.98, respectively, for heavy smoking versus never smoked,* P* < 0.05) [225]; another study reported smoking was not associated with tibiofemoral cartilage defect [228]. Interestingly, the prospective cohort study found both smoking and pack-years associated with an increased annual loss of medial but not lateral tibia or patellar cartilage [224]. There were 3 studies (2 cohorts and 1 case-control) that reported on the postoperative effects of smoking. Smokers experienced significant early meniscus repair failure* (P* = 0.0076) [223], less improvement in Modified Cincinnati Knee score after 2 years of autologous chondrocyte implantation surgery for full-thickness chondral defects of the knee* (P* < 0.05) [227], and a lower satisfaction rate after knee microfracture intervention [222].  Table 3 provides comprehensive details on the 7 studies in this subsection.

#### 3.4.2. Spinal Cartilage* (n* = 12)

Twelve studies examined the effects of smoking on spinal cartilage. One secondary data analysis reported smoking was not associated with disc degeneration and low back pain; however, the combination of smoking and hard physical work increased risk of vertebral inflammatory processes (OR = 4.9, 95%CI: 1.6-13.0) [236]. There were 11 cohort (10 retrospective, 1 prospective) studies of patients who underwent spinal surgery. Interestingly, these studies found smoking was significantly associated with an increased risk of reoperation [230, 237, 238], higher infection rates [235, 239], higher risk of 30-day morbidity* (P* = 0.04) [239], and use of analgesic medication [234, 240], but there was no consensus on spinal fusion rate, length of stay, or complication rate. Three studies reported smoking was significantly associated with a lower spinal fusion rate [232, 234, 235]; however, one study reported spinal fusion rate was not affected by smoking status [231]. One study reported smoking was associated with longer length of hospital stay* (P* < 0.001) [235], whereas the other study reported no association with length of hospital stay* (P* = 0.99) [233, 237]. Two studies reported smoking was not associated with overall complications [229, 233].  Table 3 provides comprehensive details for the 12 studies that enrolled both sexes and investigated the association of smoking with vertebral disc degeneration, pain, and the effect of smoking on spinal surgery outcomes.

### 3.5. Tobacco Smoking and Tendons (n = 6)

Three studies investigated rotator cuff tendons; two cross-sectional studies reported smokers presented with more advanced degenerative changes in their supraspinatus tendons* (P* < 0.001) [245] and reported that a higher total of smoked cigarettes was associated with the severity of rotator cuff tears (Type II versus Type I,* P* = 0.032) [242]. The retrospective cohort study in patients with calcified calcific tendinitis of the rotator cuff found smoking was significantly associated with a failure of needle aspiration of calcific deposits* (n*ACD) (adjusted OR = 1.7, 95% CI: 1.0-2.7,* P* = 0.04) [246]. One case-control study reported smokers had significantly thinner patellar and Achilles tendons in the proximal, middle, and distal thirds region of the tendons and significant lower strain ratio measurements in the same regions* (P* < 0.05); pack-years were inversely related to patellar tendon thickness* (P* < 0.05) [241]. One cross-sectional study reported smokers had significant improvement in finger range of motion over nonsmokers after tendon grafting [243]. Finally, one prospective study reported that the Constant score was significantly lower in smokers than nonsmokers 1 year postoperatively after rotator cuff reconstruction (71 versus 75,* P* = 0.017) [244].  Table 3 provides comprehensive details on the 6 studies that investigated the effects of smoking on the anatomical or functional characteristics of tendons.

### 3.6. Tobacco Smoking and Ligaments (n = 4)

Compared to nonsmokers, smokers were found to have significantly poorer outcomes regarding stability [249], Lysholm Knee Score, International Knee Documentation Committee (IKDC) subjective score, and IKDC objective grade [249, 250] after ACL reconstruction. A dose-dependent association was noted between pack-years and postoperative anterior translation (*P* = 0.015) and IKDC objective grade (*P* = 0.002) [250]. Tobacco use was associated with a significantly increased risk of postoperative venous thromboembolism (OR = 1.9;* P* = 0.035) [248] and subsequent ACL reconstruction (OR = 1.7;* P* < 0.0001) [248]; but it was not found to be significant for postoperative stiffness (OR = 0.9;* P* = 0.656) [248]. There was no consensus regarding risks of postoperative infection. One study reported tobacco use increased risk infection (OR = 2.3;* P* < 0.0001) [248], and another study reported smoking was not a significant risk factor (OR=2.5;* P* = 0.167) [247].  Table 3 provides more details on the 4 cohort studies that enrolled both sexes and investigated effect of smoking on the clinical outcomes and complications after ACL reconstruction.

### 3.7. Intrauterine and Secondhand Smoking Effect on Musculoskeletal System (n = 8)

These studies were classified into two groups: first group investigated associations between secondhand smoke and musculoskeletal system disorders, and the second investigated the effects of intrauterine exposure by mothers who smoked or mothers exposed to secondhand smoke and the long-term outcomes on the musculoskeletal system of offspring. The first group had two cross-sectional studies that reported subjects exposed to passive secondhand smoking had significantly lower phalangeal BMD (*P* < 0.01) [253] and higher risk for femoral neck osteoporosis (OR, 3.68; 95%CI: 1.23-10.92) than unexposed subjects [255]. The second group consisted of 6 studies; 3 studies were cohort studies and reported maternal smoking was significantly associated with lower aerobic fitness of male adolescents [252] and lower total body BMC in male adolescents, but not female adolescents [257]. Maternal smoking was not found to be associated with BMD [254, 257] or fractures in adolescents [254]. One prospective cohort and one cross-sectional study reported smoking by both parents during pregnancy had a significant effect on relative leg length (shorter) of offspring at ages 7-10 [258], increased spine BMC, and BMD in girls, but not boys at a mean age of 9.9 years [256]. Finally, one article, a secondary data analysis, reported exposure of nonsmoking pregnant mothers to secondhand smoke from paternal grandmothers was associated with taller girls, and greater bone and lean mass of both sexes at age 17, while exposure of nonsmokers pregnant mothers to secondhand smoking from maternal grandmothers was associated with increased weight of boys at age 17 [251].  Table 3 provides comprehensive details about the 8 studies.

## 4. Discussion

This systematic review provides evidence of the substantive negative effects of tobacco smoking on the musculoskeletal system. A majority of studies reviewed (132 of 243) focused on the deleterious effect of tobacco smoking on bones, followed by joints (54 of 243), with less emphasis on muscles, cartilage, tendons, and ligaments. At the bone level, there is sufficient evidence demonstrating tobacco smoking is associated with low BMD, an increased likelihood of fracture, delayed fracture healing, increased alveolar bone loss, increased risk of periodontitis, increased peri-implant bone loss, and implant failure. The inverse association for tobacco smoking with BMD was evident in males across ages, in adolescents of both sexes, and in postmenopausal females; however, these associations were not fully explained by biomarkers monitored to understand the mechanisms of smoking effects on bone metabolism. Studies investigating biological mechanisms were few and were limited by a lack of power or a failure to adjust for confounding variables.

The research on periodontitis provided general agreement on clinical outcomes and monitored biomarkers proposed to be affected by tobacco smoking. This may indicate different mechanisms for effects on the alveolar bone than the rest of the body or there may be factors other than smoking that have an isolated effect or interact with the effects of smoking to synergize or diminish the negative effect on human BMD. Our findings regarding the negative outcomes of smoking on bone implants and surrounding tissues were consistent with 5 systematic reviews conducted previously on smoking effects on dental implants [7–11].

The research on joints was more segregated and investigated the effects of smoking on specific joint disorders rather than whole joints. Most studies were focused on RA (29 of 54). There was consensus that smoking is associated with increased disease activity, functional disability, and poor response to therapy. There was also evidence of an interaction between smoking and HLA-DR beta 1 4-amino acid haplotype and ACPA. This interaction was significantly associated with increased RA disease activity. There was evidence of a negative effect on OA outcomes; however, findings were inconsistent. There was evidence that supported the association between smoking and increased pain in patients with TMD, increased disease activity, pain, and poor response to therapy in patients with spondylarthrosis in a pattern similar to that exhibited by patients with RA.

Studies of smoking effects on skeletal muscles provided clear evidence that smoking was associated with poor outcomes, particularly decreased muscle strength. However, these findings were inconsistent on whether smoking is associated with changes in muscle thickness or maximal voluntary contraction. For the effects of smoking on cartilage, this review provided evidence of a harmful association of smoking and pack-years with knee cartilage (increased in cartilage volume, decreased strain ratio, and poor postoperative outcome), low spinal fusion rate, and increased risk of spinal reoperation. A limited number of studies investigated the effect of smoking on tendons and ligaments. For tendons, the results found smoking and pack-years associated with thinner patellar and Achilles tendons, severe rotator cuff tears, and poor postoperative functional outcomes. The results of this review regarding the effect of smoking on tendons are consistent with the findings reported in a previous systematic review by Bishop et al. [16]. For ligaments, smoking was associated with poor functional and stability scores after ACL reconstruction, consistent with the findings in systematic reviews by Kanneganti et al. [13] and Novikov et al. [14] that reported negative effects of smoking on postoperative outcomes.

This systematic review identified few articles on secondhand (4 studies) or intrauterine exposure to smoke (6 studies). In terms of secondhand smoke, reviewed studies reported varying effects. There was an inverse association between secondhand smoke exposure and phalangeal BMD and a positive association with risk of femoral neck osteoporosis. A positive effect reported was the reduction in OA risk, and no effects were found in relation to response to RA therapy. Studies on intrauterine exposure were focused on long-term effects of exposure on the musculoskeletal outcomes of offspring. There was no consensus in the evidence on the effects of smoking on BMD, BMC, relative leg length, or other body composition parameters in male and female offspring.

This systematic review provided evidence of the negative effects of smoking on the musculoskeletal system.  Table 3 provides essential information to be considered in future studies. Definitions of smoking status and intensity of smoking based on self-report were inconsistent across studies. For example, when smoking status was treated as binomial category (smoking versus nonsmoking), one study may have added former smokers to the smoking category, while another may have placed former smokers in the nonsmoking category. Similar observations were noted regarding measurement of smoking intensity; one study may define a heavy smoker as an individual who smoked 10 cigarettes or more a day over the last 10 years, while another study may define a heavy smoker as an individual who smoked 10 or more cigarettes a day over the last 5 years. Such variations in classification may lead to a misinterpretation of overall smoking effects and introduce inconsistencies among reported findings. Objective measurements are considered more reliable assessments of smoking exposure; however, objective measurements were reported in only 12 of 243 studies, 9 of these studies measured level of cotinine, and 3 assessed levels of EXCO. We did not encounter any study in this review that measured nicotine dependence (e.g., the Fagerstrom Test for Nicotine Dependence), so we cannot conclude if there were differences in patterns of effects for use or dependence of tobacco smoking. There were a limited number of studies investigating the effects of secondhand smoke and other smoking modalities, such as hookahs, narghiles, or electronic cigarettes. This review encountered only 4 studies on secondhand smoking with varying effects reported. We also only encountered only 2 studies regarding waterpipe smoking and no studies investigated the effects of smoking electronic cigarettes on the musculoskeletal system.

This systematic review demonstrates the need for further research to understand the effects of smoking on the musculoskeletal system. Due to the limited evidence on muscles, cartilage, tendons, and ligaments, more studies using different research designs are needed. The need for studies using various research designs naturally extends to study the effects of waterpipe, electronic cigarettes, and secondhand smoke on the musculoskeletal system. Longitudinal observational and experimental studies are needed to conclusively understand the effects of smoking on bone and joint-related outcomes.

There are several factors to be considered in the design of future studies. There needs to be a consistent approach to evaluate self-reported exposure for smoking and a more objective assessment for smoking exposure. There is also a need to assess for other smoking products (polycyclic aromatic hydrocarbons, nitrosamines, etc.) rather than to assess only levels of cotinine or EXCO. There need to be more information and analysis of confounders and genetic factors that may interact with smoking effects on the musculoskeletal system. There is also a need for more and frequent monitoring for changes in smoking status in longitudinal studies. Future research should endeavor to examine more than one musculoskeletal component to shed light on the development and differentiation of cell types of the musculoskeletal system. Finally, there is a need for further research to provide insight into how to minimize the effects of smoking in patients who undergo musculoskeletal surgery. Also, we recommend more ancillary studies as part of large longitudinal studies such as Population Assessment of Tobacco and Health (PATH).

Our research had several limitations. First, this review included only English language articles. Second, in vitro studies were not included. Third, we did not search for specific diseases or disorders under the term musculoskeletal system; however, we believe our search was able to comprehensively capture these disorders that are subcategorized under each section in Table 3. Fourth, this review was focused primarily on the effect of tobacco smoking on musculoskeletal system, and due to that the search method in this review may have missed other studies that have smoking as variable. Finally, we did not find any research on electronic cigarettes with only 2 studies on waterpipes. We believe these areas warrant research and will likely attract attention in the future.

## 5. Conclusion

This systematic review provided clear evidence of the negative effects of smoking on the musculoskeletal system. Evidence found smoking associated with lower BMD, and increased risk for fracture, periodontitis, alveolar bone loss and implant failure, increased joint disease, poor functional outcomes, and poor therapeutic response. We also found evidence of adverse effects on muscles, tendons, cartilage, and ligaments, despite the scarcity of studies. As smoking continues to be an important public health concern, there is a need for further research to understand mechanisms of action for the effects of smoking on the musculoskeletal system and to increase awareness of healthcare providers and community members about the deleterious effects of smoking on the musculoskeletal system.

## Figures and Tables

**Table 1:** Summary of study characteristics.

		(1) Bones (n =132)						(2) Joints (n = 54)				(3) Skeletal Muscle (n=20)	(4) Cartilage (n = 19)		(5) Tendons (n=6)	(6) Ligaments (n=4)	(7) Secondhand Smoking (n=8)	Total
		A. Bone Mass (40)	B. Fracture (16)	C. Alveolar Bone (4)	D. Periodontitis (34)	E. Bone Implant (33)	F. Bone Graft (5)	A. Rheumatoid Arthritis (29)	B. Osteoarthritis (14)	C. Spondylarthrosis (7)	D.TMD (4)		A. Knee Joint Cartilage (7)	B. Spinal Cartilage (12)				
Study Design	Cohort	7	13	0	4	19	3	17	11	1	2	4	4	11	2	4	4	106
	Cross- sectional	27	2	2	24	7	0	7	3	5	1	5	1	0	3	0	3	90
	Case-control	1	1	1	1	3	0	3	0	1	1	1	2	0	1	0	0	16
	RCT	1	0	1	5	4	2	1	0	0	0	0	0	0	0	0	0	14
	Quasi- experimental	0	0	0	0	0	0	0	0	0	0	10	0	0	0	0	0	10
	Secondary data analysis	2	0	0	0	0	0	1	0	0	0	0	0	1	0	0	1	5
	Cross- sequential	2	0	0	0	0	0	0	0	0	0	0	0	0	0	0	0	2

Used small sample size ( reported by author)		2	6	1	7	11	2	5	0	0	0	13	0	0	2	0	0	49

Used data or sample from large study		17	3	0	2	0	0	12	2	0	0	2	0	0	0	0	4	42

Sex	Male	15	2	0	7	0	0	0	3	0	0	8	0	0	0	0	0	35
	Female	12	2	1	0	0	0	0	0	0	1	1	0	0	0	0	0	17
	Both sexes	13	12	3	27	33	5	29	11	7	3	11	7	12	6	4	8	191

Objective Measurements	Cotinine	3	1	0	3	0	0	1	0	0	0	0	0	0	0	0	1	9
	EXCO	3	0	0	0	0	0	0	0	0	0	0	0	0	0	0	0	3

**Table 2:** Summary for the effect of smoking on major outcomes of musculoskeletal health.

**Outcomes**	**Context of the body of evidence**	**Size of the body of evidence (number)**	**Quality of the body of evidence**	**Consistency of the body of evidence**	**Strength of a body of evidence**
**Effect of smoking on bones**					

Decreased BMD	Relevant and global	Large (24)	High	Highly consistent	Very strong

Decreased BMC	Relevant but specific	Small (3)	High	Low level of consistency	Limited

Increased risk for fracture	Relevant and global	Large (6)	High	Highly consistent	Very strong

Delayed fracture healing	Relevant and global	Large (5)	High	Highly consistent	Very strong

Increased alveolar bone loss	Relevant and global	Medium (9)	High	Moderately consistent	Strong

Poor periodontal parameters	Relevant and global	Large (17)	High	Highly consistent	Very strong

Increased bone implant failure	Relevant and global	Large (13)	High	Highly consistent	Very strong

Poor histometric parameters for tissues surround implants	Relevant but specific	Small (4)	High	Highly consistent	Strong

**Effect of smoking on joints**					

Increased RA disease activity	Relevant and global	Medium (5)	High	Moderately consistent	Medium

Increased remission of RA	Relevant but specific	Small (2)	High	Inconsistent	Limited

Increased RA progression	Relevant but specific	Small (2)	High	Inconsistent	Limited

Poor response to RA therapies	Relevant and global	Large (10)	High	Highly consistent	Very strong

Decreased incidence/prevalence of OA	Relevant but specific	Small (4)	High	Low level of consistency	Limited

Increased pain score associated with OA	Relevant but specific	Small (2)	High	Consistent	Medium

Increased risk for total joint replacement in OA patients	Relevant but specific	Small (2)	High	Inconsistent	Limited

Increased risk for complications after joint replacement in OA	Relevant and global	Small (4)	High	Highly consistent	Strong

Increased SA diseased activity	Relevant and global	Small (3)	Moderate	Highly consistent	Medium

Increased pain intensity in patients with TMD	Relevant and global	Small (4)	High	Moderately consistent	Strong

**Effect of smoking on muscle**					

Decreased muscle strength	Relevant and global	Medium (6)	High	Highly consistent	Very strong

Decreased maximal muscle voluntary contraction	Relevant but specific	Small (3)	High	Low level of consistency	Limited

**Effect of smoking on cartilage**					

Increased volume of knee joint cartilage	Relevant but specific	Small (2)	High	Consistent	Medium

Increased defect of knee joint cartilage	Relevant but specific	Small (2)	High	Mixed	Limited

Poor postoperative outcomes after knee joint surgery	Relevant but specific	Small (3)	High	Highly consistent	Strong

Increased risk for spinal reoperation	Relevant but specific	Small (3)	High	Highly consistent	Strong

Decreased spinal fusion rate	Relevant and global	Small (4)	Moderate	Moderately consistent	Medium

**Effect of smoking on tendons**					

Increased severity of rotator cuff tears	Relevant but specific	Small (2)	High	Consistent	Medium

Poor outcomes after rotator cuff reconstruction	Relevant but specific	Small (2)	High	Consistent	Medium

**Effect of smoking on ligaments**					

Poor functional and stability scores after ACL reconstruction	Relevant and global	Small (4)	High	Moderately consistent	Medium

**Table 3:** Tobacco smoking and musculoskeletal system.

Citation	Design	Sample Size/Age/Sex/Type of Sample	Study Purpose	Findings
**Section 1: Bone (132 Articles)**				

***1.A: Tobacco Smoking and Bone Mineral Density (BMD), Bone Mineral Content (BMC), and Bone Turnover (BT) (40 Articles)***				

Beyth et al., 2015 [17]	Cross-sectional study	(i) N = 26 iliac bone marrow samples collected during pelvic surgery: 13 nonsmokers, 13 smokers (ii) The average of age (Mean ± SD): 36.0 ± 11.87 for nonsmokers 39.0 ± 12.91 for smokers (iii) 16 were males, and 10 were females	(i) To investigate whether smoking is associated with lower levels of bone marrow progenitor cells (BMPCs) express CD15 which is necessary for musculoskeletal healing/regeneration	(i) Compared to nonsmokers, smokers had lower average concentration of BMPCs (3.52 x 105 /mL ± 2.45 x 105 /mL versus 1.31 x 105 /mL ± 1.61 x 105 /mL for smokers, t = 3.2, P = 0.004)

Bjarnason et al., 2009 [18]	Post hoc exploratory study based on data from RCT study	(i) N = 270: 192 nonsmokers, 78 smokers (ii) The average of age (Mean ± SD): 53.5 ± 1.9 for nonsmokers 53.1 ± 1.6 for smokers (iii) All participants were postmenopausal Caucasian women	(i) To investigate the influence of smoking on bone during therapy (nasally administrated estradiol with oral progesterone) in early postmenopausal women, and to observe the impact of smoking on bone in untreated women	(i) During treatment with nasal estradiol, the BMD of the lumbar spine for smokers had increased at 2 years but with less than that in nonsmokers (2.6% versus 3.9%, P = 0.03). Similar trend exhibited among controls (-3.6% versus -2.4%, P = 0.08) (ii) When smokers compared to nonsmokers, there was no difference in the response to estradiol in hip BMD (P = 0.89), whereas the change in the hip on the placebo was similar to that seen in the spine (P = 0.08). Supportive changes were seen within urinary CTX and serum OC

Breitling, 2015 [19]	Cross-sectional study [sample NHANES III)]	(i) N = 14,116: 6,882 never-smokers, 3,532 former smokers, 3,702 current smokers (ii) The median age of the participants was 44 years (iii) 6,830 were males, and 7,286 were females	(i) To examine the relationships between BMD and calcium intake (dietary) and to examine the interaction (smoking and calcium intake) on BMD	(i) There was an overall positive trend between calcium intake and bone mineral density among three smoking behavior categories (ii) The interaction of smoking with calcium intake on BMD did not reach statistical significance and the dose-response curves became more similar across smoking behavior strata after adjustment for several factors (BMI and physical activity)

Callreus et al., 2013 [20]	Cross-sectional study [sample from PEAK-25 cohort]	(i) N = 1,054: 591 never-smokers, 187 former smokers, 276 current smokers (ii) All participants were 25-year-old women	(i) To evaluate the association between smoking and bone mass (BMD and fracture risk) in 25-year-old women	(i) BMD and relative fracture risk did not differ between never-smokers and former and current smokers (ii) Among current smokers, BMD (femoral neck) decreased as cigarette consumption increased in dose-response effect (P = 0.037) (iii) BMD was not significantly lower in young women who had smoked for long duration (P = 0.07) or started smoking early (P = 0.64) (iv) After smoking cessation, lower BMD persisted up to 24 months, becoming comparable to never-smokers after 24 months

Cangussu et al., 2012 [21]	Cross-sectional study	(i) N = 225 women in amenorrhea: group 1: 140 with T-score > -2.0 SD (116 nonsmoker, 24 current smokers) and group 2: 85 with T-score ≤ -2 SD (72 nonsmokers, 13 current smokers) (ii) The average of age was 54 (50-57) for patients group 1, and it was 58 (52-64) for group 2 (iii) All participants were postmenopausal women	(i) To evaluate association of BMD with postural balance in postmenopausal women and its relation to risk for falls and to investigate if smoking increase the risk for fall.	(i) Compared to those with BMD ≤ -2 SD, patients with BMD > -2.0 SD were found to be younger, had shorter time since menopause, and had higher BMI (P < 0.05) (ii) The fall episodes were reported in 57.8% of the participants without any significant differences between the two groups of BMD (iii) The postural balance assessed via stabilometric parameters was not differed between the two groups (P > 0.05) (iv) The risk of fall increased with current smoking (OR = 2.19, 95%CI: 1.22-3.21), age, and corrected visual deficit. However, hormone therapy use was significantly correlated with reduced risk for falls (OR = 0.48, 95%CI: 0.26-0.88)

Caram et al., 2016 [22]	Cross-sectional study	(i) N = 96: 32 never-smokers, 32 smokers (>10 pack-year), and 32 mild/moderate COPD (current or former smokers) (ii) The average of age (Mean, range): (49.5, 46-58) for never-smokers, (53, 5-55) for smokers, and (64.5, 58-74.5) for COPD patients (iii) 28 were males, and 68 were females	(i) To investigate the joint effect of smoking and COPD on the health status, body composition, and exercise capacity	(i) Compared to never-smokers, patients with COPD had lower fat-free mass (FFM) [P = 0.02] and fat-free mass index (FFMI) [P = 0.008](ii) Six-minute walk distance (6MWD) was lower in COPD and smokers than never-smokers (P = 0.01) (iii) Compared to never-smokers, smokers had worse SF-36 score for functional capacity (P < 0.001) (iv) Compared to smokers and never-smokers, COPD patient had lower SF-36 score for physical functioning (P < 0.001) and role-emotional (P < 0.001) (v) Both COPD diagnosis and smoking inversely associated with FFMI, 6MWD and health status

Cetin et al., 2009 [23]	Cross-sectional study	(i) N = 60: 30 nonsmokers, and 30 smokers (ii) The average of age (Mean ± SD): 51.2 ± 3.4 for nonsmokers, 49.7 ± 3.5 for smokers (iii) All participants were postmenopausal women (iv) Participants were randomly selected	(i) To investigate the impact of smoking on the oxidative status in postmenopausal women and to assess the relationship between BMD and oxidant/antioxidant parameters	(i) The rates of osteopenia and osteoporosis in smokers and nonsmokers were 75% and 52.5%, respectively (ii) The T-scores were significantly lower in smokers than in nonsmokers (median: -2.7 versus -1.4, P < 0.001) (iii) Activities of antioxidant enzymes (superoxide dismutase, glutathione peroxidase, and paraoxonase) were lower and the levels of oxidative stress products (malondialdehyde, nitric oxide) were higher in smokers than in nonsmokers (P < 0.001) (iv) In the smoking group, there was a significant correlation between decreased T-score and oxidative stress parameters

Chassanidis et al., 2012 [24]	Cross-sectional study	(i) N = 105: 45 fractured bone (26 nonsmokers, 19 smokers), and 60 nonfractured bone (39 nonsmokers, 21 smokers) (ii) The average of age (Mean, Rang): 49 (21 to 87) for fractured, and 52 (19 to 82) for nonfractured (iii) 62 were males, and 43 were females	(i) To evaluate the effect of smoking on the expression of the bone morphogenetic proteins (BMPs) of human periosteum	(i) Compared to nonsmokers, smokers remarkably had lower reduction in the gene expression of BMP-2, BMP-4, and BMP-6, demonstrating the effect of smoking on periosteal mRNA transcription for BMPs (ii) Compared to fractured group, nonfracture groups exhibited a higher gene expression of BMP-2, BMP-4 and, BMP-7

Chen et al., 2016 [25]	Cross-sectional study	(i) N = 131: 36 nonsmokers, 38 smokers had normal lung function and 57 had COPD (ii) The average of age (Mean ± SD): 74.0 ± 9.49 nonsmokers, 72.44 ± 12.27 smokers, and 77.7 ± 10.89 (iii) All participants were male	(i) To evaluate the expression of receptor activator of nuclear factor-B ligand in CD4 (+)/CD8 (+) T cells and Th17 cells and the role of these cells in COPD and smoker population in bone loss	(i) Compared to nonsmokers and COPD patients, smokers had higher frequencies of RANKL-CD4 (+) and CD8 (+) T cells (All P < 0.001). (ii) COPD patients had significantly higher proportion of CD(+) T cells expressed RANKL and IL-17 than that of nonsmokers (P = 0.010). (iii) All groups had similar frequency for RANKL expression in Th17 (P = 0.508). (iv) There were an inverse correlation between the frequency of RANKL (+) CD4 (+) T cells and BMD of the lumbar vertebrae (r =-0.229, P = 0.01) and femoral neck (r=-0.350, P < 0.001).

Christie et al., 2009 [26]	Cross-sectional study	(i) N = 69 twin (ii) Average of age (Mean ± SD): 53 ± 8.9 with a range of (40-76) years (iii) 13 were males, and 56 were females	(i) To examine whether mechanism of bone loss in pair twins could be related to smoking	(i) Percentage within-pair difference (WPD) that was calculated based on the differences between smokers and nonsmokers were found to be significant for BMD of femoral neck (-5.6%, 95%CI: -9.0 to -2.2, P = 0.002), total hip (- 6.2%, 95%CI: -9.4 to -2.9, P ≤ 0.001), and whole-body BMC (- 4.1%, 95%CI: -7.2 to -1.1, P = 0.012). However, it was found to be not significant for lumbar spine (-3.5%, 95%CI:(-7.0 to 0.0, P = 0.058), and forearm (-0.8%, 95%CI: -2.6 to -1.0, P = 0.290). (ii) WPD for fat mass was also lower in smoking twins (-12.8%, 95%CI:-20.7 to -4.8, P = 0.005), and lean mass marginally significant (-2.8, 95%CI: -5.9 to 0.3, P = 0.083)

De Jong et al., 2014 [27]	Cross-sectional study [sample from the NELSON trial]	(i) N = 1,140: 609 current (≥16 pack-year) and 531 former smokers (ii) 437 participants had COPD (iii) The average of age (Mean ± SD): 62.5 ± 5.2 (iv) All participants were males.	(i) To investigate the association of smoking and COPD with BMD and vertebral fracture.	(i) The prevalence of vertebral fracture among the cohort was 8.8% (100). Current smokers had higher rate of vertebral fracture than that of former smokers (11.3% versus 5.8%, P = 0.001), but it was not different based on COPD status (9.6% versus 8.3%, P = 0.430) (ii) The adjusted odds ratio (AOR) for fracture was “AOR = 1.79. 95%CI: 1.13-2.84” in current smokers and “adjusted OR = 1.08, 95%CI: 0.69-1.67” in COPD participants. Both smoking and COPD were independently associated with BMD adjusted for age and BMI (iii) BMD assessed by Hounsfield Units (HU) was lower in current than in former smokers (103.2HU versus 108.7HU, P = 0.006) and was lower in COPD patients than patients without COPD (100.7 HU versus 108.9HU, P < 0.001)

Dorn et al., 2013 [28]	Cross sequential design	(i) N = 262: 171 nonsmokers, 91 smokers (ii) The average of age (Mean ± SD) at time 1: 14.35 ± 2.16 years (iii) All participants were females (iv) 32% black, and 62% white	(i) To examine the impact of depressive and anxiety symptoms, smoking, and alcohol use on bone accrual in girls 11-19 years with age cohort of 11, 13, 15, and 17 years	(i) The lower rates of lumbar spine and total hip BMD of ages 13-19 were associated with higher frequency of smoking (ii) There was an association between high depressive symptoms and lower lumbar spine BMD across 11-19 years of age (iii) Alcohol intake and anxiety had no effect on bone outcome, and depressive symptoms had no effect on total body BMC

Dorn et al., 2011 [29]	Cross sequential design	(i) N = 262: 171 nonsmokers, 91 smokers (ii) The average of age (Mean ± SD): 14.9 ± 2.2 years (iii) All participants were females (iv) The majority were Caucasian (61.8%) or African American (32.8%) with some mixed race/other (5.4%)	(i) To examine the association between depressive and anxiety symptoms, smoking, and alcohol use on bone health whether the association between depressive and anxiety symptoms varied by smoking or alcohol use individually or by combined use	(i) The higher state of depressive symptoms was associated with lower BMC and BMD (ii) Participants with lowest use of smoking had higher BMD (hip, femoral neck), however; no differences were observed by alcohol use (iii) Compared with alcohol (regular, nonusers), the regular users of both cigarettes and alcohol had a stronger negative association between depressive or anxiety symptoms and total body BMC

Dorn et al., 2008 [30]	Cross-sectional study	(i) N = 207: 107 never-smokers, 94 ever-smokers (ii) The average of age (Mean ± SD): 14.0 ± 2.2 years (iii) All participants were females. (iv) 62.2% white, 32.3% African American (32.3%), and 5.5% mixed race/other	(i) To investigate the relationships between anxiety, depression, smoking, and BMD among adolescent girls	(i) Higher depressive symptoms were correlated with lower total body BMC and BMD but not associated with hip or spine BMC and BMD. (ii) In white women, the lower levels of total body BMC and hip BMC and BMD were associated with high score of anxiety. (iii) There were no differences in age-adjusted BMC or BMD between ever-smokers and never-smokers. However, ever-smokers had higher depressive and anxiety symptoms than never-smokers. (iv) There was no significant interaction between smoking status and depression or anxiety status. (v) High level of anxiety was associated with lower BMC in group of ever-smoking

Drage et al., 2007 [31]	Cross-sectional study	(i) N = 18: 5 nonsmokers, 13 smokers (ii) The average of age (Mean ± SD): 67.1 ± 12.6 (iii) 9 were males, and 9 were females	(i) To investigate the association between jaws BMD and other skeletal site BMD and to investigate the influence of smoking on the BMD	(i) The BMD of the ramus was similar to the femur; however it was significantly lower than the BMD of lumbar spine. (ii) BMD of anterior maxilla was lower than BMD values of femur and ramus. (iii) There was a strong relationship between ramus BMD and spine and hip BMD, but there were no relationships between BMD of other jaw's areas and BMD skeletal sites. (iv) There was an inverse association between increasing age and BMD of both hip and the ramus. However, there was no significant association between BMD of hip, spine, and jaw and years edentulous or cigarette years

Eleftheriou et al., 2013 [32]	Retrospective cohort study	(i) N = 723 healthy male military recruits: 329 nonsmokers, 41 Ex-smokers, 35 recent Ex-smokers, and 244 current smokers (ii) The average of age was 19.92 with a range of 16-18 years. (iii) All participants were Caucasian males	(i) To investigate the influence of young men lifestyles factors of smoking, alcohol, and physical activity on the peak bone mass as evidenced by the changes on the bone structure and geometry	(i) Smoking was associated with well-maintained bone geometry but worse BMD (P = 0.0001) and calcaneal quantitative ultrasound (QUS) (P ≤ 0.0005). (ii) Alcohol consumption at moderate level was associated with higher BMD (P ≤ 0.015). (iii) The increment in weight-bearing exercise was associated with improved periosteal bone apposition and total hip and femoral neck BMD (P ≤ 0.0001) at cortical (P < 0.0001) and periosteal level (P = 0.016)

Emaus et al., 2014 [33]	Prospective cohort study [sample from Tromso study in Northern Norway]	(i) N = 4,664 at baseline (ii) The range of age for all participants was 30-80 years (iii) Baseline visit: 2,084 were males, and 2,580 were females (iv) Second visit: 1,113 were males, and 1,401 were females	(i) To examine the effect of combined profiles of smoking, physical activity, and BMI on lifetime bone loss	(i) When study compared peaks at ages 40 to 80 years, the group of nonsmoking, physically active men with BMI of 30 kg/m2 had varied loss rate at femoral neck (4% to 14%), while loss rate reached approximately 30% at femoral sites in group of heavy smoking, physically inactive men with a BMI value of 18 kg/m2 (ii) BMI was detrimental factor for bone loss in women; the estimated loss in lifestyle groups with BMI of 18 kg/m2 was more than 30%

Fujiyoshi et al., 2016 [34]	Cross-sectional study	(i) N = 376: 240 never-smokers, 64 former smokers, 72 current smokers (ii) The range of age was 24-36 years (iii) 205 were males, and 171 were females (iv) 181 were white, 195 were black	(i) To examine whether smoking was associated with serum parathyroid hormone (PTH) independent of correlates of PTH among young adults and explore potential mechanisms	(i) Compared to nonsmokers, current smoker had lower PTH and there was no evidence of an interaction by race and sex. (ii) The lowest level of PTH was detected in current smokers followed by former smokers (intermediate level), while the highest levels were seen in never-smokers (mean of PTH: 23.6, 26.7, 27.4 pg/mL, respectively: P for trend = 0.006, adjusted for calcium intake confounders). (iii) Current smoker had the lowest biomarkers concentration of serum osteocalcin and 24-hour urinary excretion of calcium.

Giampietro et al., 2010 [35]	Nested case-control study [sample from Marshfield Clinic Personalized Medicine Cohort]	(i) N = 376: 309 with osteoporosis (cases) [217 never-smokers, 15 former and 90 current smokers], and 293 with normal BMD (controls) [176 never-smokers, 27 former and 29 current smokers] (ii) The average of age (Mean ± SD): 70.4 ± 9.4 for cases and 61.6 ± 8 for control (iii) All participants were Caucasian postmenopausal women	(i) To examine the impact of statin use, cigarette smoking, and genetic polymorphism on development of osteoporosis	(i) There was an association among IL6-634G > C (rs1800796) and osteoporosis (OR for CC + CG = 2.51, P = 0.0047)), independent of statin use or smoking status. (ii) Based on smoking stratification, there was an association between lipoprotein receptor-related protein 5 (LRP5) C135242T (rs545382) and osteoporosis emerged (OR = 2.8, P = 0.03) which suggested a potential environmental interaction

Kargin et al., 2016 [36]	Cross-sectional study	(i) N = 170: 85 nonsmokers, and 85 smokers (ii) The average of age ( Mean ± SD): 43.58 ± 6.58 nonsmokers, and 43.52 ± 6.72 smokers (iii) All participants were males	(i) To compare the bone turnover markers between smoker and nonsmoker males	(i) Smoker's C-terminal telopeptide (CTX) level was significantly lower than that of the nonsmokers ((0.30 ± 26.97 ng/ml versus 65.10 ± 42.41 ng/ml, P = 0.007) (ii) Smoker's mean serum parathyroid hormone (PTH) level was significantly lower than that of nonsmokers (23.75 ± 9.88 pg/ml versus 31.35 ± 13.15 pg/ml, P ≤ 0.001), and parallel findings were observed for vitamin D (16.75 ± 8.73 ng/ml versus 19.50 ± 8.97 ng/ml, P = 0.044)

Kassi et al., 2015 [37]	Cross-sectional study	(i) N = 181: 117 nonsmokers, and 64 smokers (ii) The average of age ( Mean ± SD): 34.69 ± 7.38 with a range of age 20-50 years (iii) All participants were males (iv) Random sample	(i) To determine the prevalence of vitamin D (25(OH)D, D-2 and D-3) insufficiency and its association with smoking, BMD, and bone markers	(i) The prevalence of 25(OH)D < 20ng/ml) was 50.3%. (ii) There was a strong correlation between 25 (OH) D and smoking (P < 0.001), and 25(OH)D level was significantly lower in smokers than in nonsmokers. (iii) In total population, regardless of the age group, there were no correlations between 25(OH)D and BMD in the femoral neck and the lumbar spine. Also, 25(OH)D was not correlated with bone turnover markers: serum osteocalcin, P1NP, and b-CTXs levels.

Kaume et al., 2014 [38]	Randomized Controlled Trial (RCT)	(i) N = 65: 20 nonsmokers, and 45 smokers randomly assigned to: smoker control (n = 21); smoker received blackberries (n = 8); or smoker received blueberries (n = 16) (ii) The range of age was 46.5-68.0 years (iii) All participants were males, and 98% were white	(i) To determine whether the consumption of antioxidant-rich berries prevents smoking-induced bone loss in postmenopausal women	(i) The bone loss of the total body BMD was significant in smoker control and smoke blueberries groups, but not in nonsmokers and smokers blackberries groups (P = 0.0284)

Kim et al., 2007 [39]	Cross-sectional study	(i) N = 463: alcohol-only drinking (n = 254), combined alcohol drinking and smoking (n = 125), and control nondrinking/nonsmoking (n = 84) (ii) Average of age was 23 and the range of age was 20-26 years (iii) All participants were Korean males	(i) To investigate effects of alcohol and tobacco smoking on BMD and bone metabolism	(i) There were no significant differences in BMD of the calcaneus among the 3 groups. However, blood total alkaline phosphatase activity (ALP) was significantly lower in the combined drinking and smoking group than in the control group (P < 0.05). (ii) There were negative relationships between duration of alcohol consumption and ALP N-mid osteocalcin levels (all P < 0.001). (iii) Daily cigarette use and smoking duration showed a significantly negative correlation with ALP (P < 0.001)

Klefter et al., 2012 [40]	Prospective cohort study [data from the KIMS and NordiNet]	(i) N = 102 GHD patients: 66 nonsmokers, 36 smokers (ii) Median of age was 42.7 and the range of age was 14.7-75.5 years	(i) To determine if body composition and BMC and BMD differed between smokers and nonsmokers with growth hormone- (GH-) treated GH-deficiency (GHD)	(i) At baseline there were no differences in body composition or bone parameters between smokers and nonsmokers. (ii) With later visits after 3-5 years of GH therapy, lean body mass (LBM) had increased by 10.3% in smokers and 10.5% in nonsmokers (both P < 0.0001 versus baseline). (iii) With later visits after 3-5 years of GH therapy, total BMC had increased by 1.8% (P < 0.05) in nonsmokers, while the changes in smokers were not statistically significant (P = 0.09). (iv) Increase in lumbar BMD was seen in both groups, but it was significantly greater among nonsmokers (6.5% versus 2.6%, P < 0.05).

Kleppinger et al., 2010 [41]	Secondary data analyses from RCT	(i) N = 152 underwent smoking cessation using nicotine patch: 42 quitters, 77 continued smoking (ii) The mean of age was 56 ± 7, and the range of age was 41-78 years (iii) All participants were postmenopausal women	(i) To examine the effects of 16 months of cigarette abstinence on body composition (BC)	(i) Over time and after controlling for the baseline BC measures and other confounders, quitters had significantly increased body weight (P < 0.001), fat mass (P < 0.001), and functional muscle mass (P = 0.004)

Kooij et al., 2015 [42]	Cross-sectional study [sample from AGEhIV Cohort Study]	(i) N = 1,101: 581 HIV-positive receiving combination antiretroviral therapy (cART) [231 nonsmokers,172 current, 178 former smokers], and 520 HIV-negative [200 nonsmokers,122 current, 198 former smokers] (ii) Age ≥ 45 years, and all participants were males	(i) To examine the association of BMD with body weight and smoking based on HIV status	(i) Osteoporosis was significantly prevalent among HIV-positive rather than HIV-negative patients (13.3% versus 6.7%; P < 0.001). However, such association was not observed after adjustment smoking and body weight

Kuo et al., 2008 [43]	Cross-sectional study	(i) N = 837: 532 never, 258 current, 47 former smokers (ii) The average of age was 52.2 ± 6.7 with a range of 46-60 years. (iii) All participants were males	(i) To investigate the effect of cigarettes smoking on the BMD in healthy Taiwanese middle-aged men	(i) Compared to never-smokers, current smokers had significantly lower lumbar BMD (P = 0.024), but femoral neck BMD was found to be similar in smokers and nonsmokers. (ii) Both former smokers and never-smokers had similar BMD at any measured sites, indicating that quitting smoking had a positive effect on BMD. (iii) There was a negative association between smoking and lumbar BMD (r = -0.166, P = 0.004), but there no association was seen between smoking and femoral neck BMD.

Lee et al., 2014 [44]	Cross-sectional study [sample from KNHANES]	(i) N = 770 (ii) Range of age was 30-95 years (iii) All participants were males	(i) To investigate an association between urinary cotinine and BMD in femoral neck and lumbar spine in male older than 30 years.	(i) The means of femoral neck BMD (T-score) significantly decreased with increasing age (P < 0.001). The same trend was observed in lumbar spine BMD. Although education (P < 0.001) and income (P = 0.021) were associated with femoral neck T-score, only education (P = 0.034) was associated with lumbar spine T-score. (ii) Compared to group with ≤ 10 *μ*g/mL cotinine, group with > 10 *μ*g/mL of cotinine had lower femoral neck T-score (-0.43 ± 0.98 versus -0.33 ± 0.89, P = 0.114). (iii) According to multiple linear regressions, age, urine cotinine, and BMI were related to femoral neck and lumbar spine T-score.

Liu et al., 2013 [45]	Cross-sectional study	(i) N = 231 (ii) The range of age was 20-39 years (iii) All participants were males	(i) To examine whether the genetic polymorphism of Glutathione S-transferases (GSTM1, GSTT1) influence effects of smoking on bone.	(i) Genotypes were determined for null alleles of GSTM1 and GSTT1. (ii) Among subjects with GSTM1 null genotype, smoking was inversely associated with speed of sound (SOS) (beta = -0.28, 95%CI:-34.01 to -8.65) and bone quality index (BQI) and “beta = -0.30, 95%CI, -16.41 to -4.49, respectively.” P for interaction = 0.04 for SOS and 0.08 for BQI (iii) Among those with GSTM1 present allele, smoking was not associated with SOS and BQI (beta = -0.02, 95%CI: -15.01 to 12.59, and beta = -0.09, 95%CI: -8.75 to 3.02, respectively). No interaction was found between the GSTT1 polymorphism and smoking.

Lorentzon et al., 2007 [46]	Cross-sectional study [ sample from GOOD]	(i) N = 1,068: 975 nonsmokers, 93 smokers (ii) The average of age (Mean ± SD): 18.9 ± 0.6 for nonsmokers, and 19.0 ± 0.6 years for smokers (iii) All participants were young males	(i) To investigate if smoking habit associated with bone size and areal or volumetric BMD (aBMD or vBMD)	(i) Smokers had significantly lower aBMD of the total body, lumbar spine, and trochanter than nonsmokers. (ii) Smokers had lower cortical thickness of both the radius and tibia than the nonsmokers, whereas no difference was seen for cortical vBMD. (iii) Smokers had higher levels of testosterone (total and free) and lower 25-OH-D than nonsmokers and the adjustment for such differences did not alter the associations between smoking and bone parameters.

Lucas et al., 2012 [47]	Prospective cohort study	(i) N = 731: at age 13 years, one-fourth tried smoking, while 59% used alcohol and 20% had both (ii) The range of age was 13-17 (iii) All participants were females	(i) To quantify the short-and long-term associations between smoking and alcohol drinking initiation and bone mineral density in adolescent girls	(i) Lower mean BMD was observed at age of 17 years (late adolescence) in girls who had ever smoked by 13, and similar trend was observed for those that consumed alcohol at that age

Myong, et al., 2013 [48]	Cross-sectional study [data from KNHANES IV]	(i) N = 4,260 (ii) The range of age was 13-17 (iii) All participants were females	(i) To evaluate the dose-dependent relationship between smoking (urinary cotinine) and BMD in females among Korean females.	(i) There were significant linear relationships between urinary cotinine and BMD of femoral neck, total femur, and lumbar spine (beta = -0.006, -0.006, and -0.008, respectively, all P < 0.05).

Oyen et al., 2014 [49]	Prospective community-based cohort study [sample from The Hordaland Health Study]	(i) N = 7,074 (ii) The range of age was 46-49 for first cohort, and 71-74 years for second cohort (iii) Both cohorts had participants from both sexes	(i) To examine the associations of plasma choline and betaine with BMD, the risk of hip fractures, and possible effect modification by nicotine exposure	(i) Participants in the lowest and middle tertiles of plasma choline had an increased risk of low BMD (OR = 2.00, 95%CI: 1.69-2.37, OR=1.39, 95%CI: 1.17-1.66, respectively, P trend<0.001) (ii) Elderly women in the lowest and middle tertile of plasma choline had significantly increased risk for low BMD (OR = 2.84, OR = 1.80, respectively). The highest OR was seen in elderly women in lowest tertile of choline who were exposed to nicotine (OR=4.56, 95%CI: 1.87-11.11). (iii) In elderly women and men, low plasma choline was also associated with an increased risk of hip fracture (lowest tertile: HR=1.45, 95%CI: 1.08-1.94; middle tertile: HR =1.13, 95%CI: 0.83-1.54, p trend=0.012). Plasma betaine was not related to BMD or hip fracture.

Pompe et al., 2017 [50]	Prospective cohort study [side study from NELSON]	(i) N = 408: at baseline 197 current and 211 Ex-smokers (ii) The average of age (Mean, Range): 59.4, 55.9-63.5 years (iii) All participants were males	(i) To examine the association of current and former smoking with BMD decline after 3-year follow-up	(i) At baseline, BMD was not significantly deferred between current and former smokers (P = 0.96). (ii) Later after 3 years, there was a decline in BMD of current smokers (P = 0.001); however there were no changes in BMD of former smokers (P = 0.34). (iii) Current smoking remained independently associated with BMD decline after adjustment for baseline BMD and BMI (−3.8 HU, p = 0.003). However, age, pack-year, and presence of fracture at baseline was not associated with BMD decline

Rom et al., 2015 [51]	Cross-sectional study	(i) N = 49 heavy smokers (ii) The average of age (Mean, Range): 43.8 ± 12.0 with a range of 22-64 (iii) 23 were males, and 26 were females	(i) To compare the assessment for body composition of segmental bioelectrical impedance analysis (sBIA) with the method of dual energy X-ray absorptiometry (DEXA)	(i) There were high correlations between sIBA and DEXA in regard to whole-body fat and lean mass (r > 0.9, p < 0.001). (ii) Lesser degrees of agreement were found for BMC, appendicular lean mass, and fat mass

Rudang et al., 2012 [52]	Prospective cohort population-based study (5-year longitudinal) [sample from GOOD]	(i) N = 833: heavy smokers (ii) The range of age was 18020 at baseline (iii) All participants were males	(i) To examine the cross-sectional associations between current smoking and trabecular microarchitecture of the radius and tibia. Also, to monitor such association after 5 years of follow-up	(i) Compared to nonsmoker men at baseline and during follow-up, men started smoking since baseline had smaller increase in the mean of areal bone mineral density (aBMD) at the total body (P < 0.01) and lumbar spine (P = 0.04) and substantially greater decreases in aBMD at the total hip (P < 0.01) and femoral neck (P < 0.01). (ii) Men who had started to smoke had a minor increment of the tibial cortical cross-sectional area (CSA) (P = 0.03), and a larger decrement of trabecular volumetric BMD (vBMD) (P < 0.001) than men who were nonsmokers. (iii) The follow-up finding shows that the tibial trabecular vBMD was significantly lower in smokers than in the nonsmokers (7.0%, P < 0.01).

Sneve et al. 2008 [53]	Cross-sectional study. [sample from Tromso Study]	(i) N = 2,810 (ii) The average of age (Mean ± SD): 65.5 ± 9.5 for males, and 63.6 ± 10.0 for females (iii) 1,442 were males and 1,368 for females	(i) To explore the relation between serum PTH and BMD, adjusted for lifestyle factors including smoking.	(i) In both genders, age, BMI, and serum PTH were strong predictors of hip BMD. (ii) There was no significant relation between serum PTH and BMD at the distal or ultradistal forearm. (iii) The relation between PTH and BMD at the hip was significant only in current nonsmokers given that the data of smokers and nonsmokers were analyzed separately. (iv) Compared to current smokers, current nonsmokers had significantly higher BMD, while former smokers had BMD values located in between current smokers and never-smokers.

Szulc et al., 2011 [54]	Cross-sectional study [sample of STRAMBO study]	(i) N = 810: 261 never-smokers, 502 former smokers, 47 current smokers (ii) The range of age was 60-87 years (iii) All participants were males	(i) To investigate the association of trabecular microarchitecture at distal radius and distal tibia and smoking in male population	(i) Compared with never-smokers, current smokers had lower trabecular number and trabecular volumetric density, more heterogeneous trabecular, higher urinary deoxypyridinoline, and CRP. However, there were no differences in cortical parameters and aBMD between the current smokers and never-smokers. (ii) Former smoker's aBMD and all bone microarchitectural parameters did not differ from the never-smokers

Tamaki et al., 2011 [55]	Cross-sectional study [sample from FORMEN study]	(i) N = 1,576: 366 never-smokers, 1,210 ever-smokers (933 former smokers, 277 current smokers) (ii) The average of age (Mean ± SD): 73.1 ± 5.2 years (iii) All participants were males	(i) To examine whether smoking and its cessation influence bone status and metabolism in Japanese men ≥ 65 years	(i) There were significant differences in the lumbar BMD between the three groups of never-smokers and former and current smokers (P = 0.005). However, total hip BMD was found to be similar between the three groups (P = 0.078). (ii) Among never- and ever-smokers, lumbar spine and total hip BMD decreased with the number of pack-years or the number of smoking years, respectively. (iii) Smoking did not reveal significant effect for serum osteocalcin (OC) or tartrate resistant acid phosphatase isoenzyme 5b (TRACP 5b).

Tamaki et al., 2010 [56]	Cross-sectional study [sample from JPOS Study]	(i) N = 789: 664 never-smokers, 125 ever-smokers (ii) The range of age was 20-40 years (iii) All participants were females	(i) To examine whether smoking exposure influences BMD or the risk of low bone status in premenopausal women	(i) Compared with never-smokers, ever-smokers had lower lumbar spine BMD (adjusted OR = 2.03, 95%CI: 1.12 - 5.82). (ii) The OR for bone status (lumbar spine) was 1.59 (95%CI: 0.65 - 3.91) in those with < 3 pack-years of tobacco use and 2.55 (95%CI: 1.12 - 5.82) in those with 3 or more pack-years of tobacco use

***1.B: Tobacco Smoking and Fracture (16 Articles)***				

Clement et al., 2016 [57]	Retrospective cohort study	(i) N =92 cases of displaced midshaft fractures: 71 nonsmokers, 21 smokers (ii) Average age in years (Mean ± SD): 32.2 ± 11.4 (iii) 80 were males, and 12 were females	(i) To identifies early risk factors for symptomatic nonunion of displaced midshaft fractures of the clavicle and to identify of an at-risk group who may benefit from the surgery.	(i) There were 13 patients (14%) with symptomatic nonunion requiring surgery. (ii) The independent predictors for nonunion were smoking (OR = 40.76, 95%CI: 1.38 - 120.30) and the six-week Disabilities of the Arm Shoulder and Hand (DASH) score (OR = 1.11, 95%CI; 1.01 - 1.22, for each point increase). (iii) The threshold value to predict nonunion was a DASH score of 35 or more at six weeks. (iv) The presence of smoking and the threshold value of DASH increased the risk of nonunion to 44%, and when only one of them is present this risk reduced to 17% and to 20%, while when neither of them was present the risk was reduced to 2%.

Dinah et al., 2007 [58]	Retrospective cohort study	(i) N = 34 patients underwent 37 operations: 20 nonsmokers, 17 smokers (ii) Average of age was 26.8 with a range of 13.4 – 52.9 years (iii) 32 were males, and 2 were females	(i) To examine the effect of smoking on the operative management for nonunion carpal scaphoid	(i) The success rate of the all kinds of operations was 59.5% (22/37). This success rate was 82.4% in nonsmokers and dropped to 40.0% in smokers (P < 0.01). (ii) There was a significant association between smoking and nonunion (P = 0.02).

Du et al., 2011 [59]	Cross-sectional study	(i) N = 703: 281 nonsmokers, 422 smokers (former and current) (ii) Mean of age was 93.48 years (iii) 226 were males, and 477 were females	(i) To observe the relationships of osteoporotic fracture with habits of smoking, tea consumption, alcohol consumption, and exercise among very old unrelated Chinese nonagenarians and centenarians	(i) In older Chinese people, there were significant associations between the increased risk of osteoporotic fracture and current or former alcohol drinking, and the risk for osteoporotic fracture was significantly reduced with habit of former exercise. Smoking and tea consumption were found not to be associated with osteoporotic fracture. (ii) The former habit of alcohol consumption was significantly associated with a greater risk of osteoporotic fracture (OR = 2.47, 95%CI: 1.07- 5.53), but the former habit of exercise was associated with a lower risk of osteoporotic fracture

Hernigou & Schuind, 2013 [60]	Retrospective case-control study	(i) N = 116: 38 diaphyseal nonunions (10 femurs, 16 tibias, and 12 humerus) and 76 age-sex-location matched controls. (ii) The average of age was 42 with a range of 16-85 years. (iii) 87 were males, and 27 were females	(i) To impact of tobacco abuse in the consolidation of diaphyseal fracture (femur, tibia, or humerus)	(i) Regardless of whether the fracture was open or closed, tobacco smoking was significantly associated with nonunion (P < 0.01)

Jorgensen et al., 2011 [61]	Prospective cohort study (Sample from Tromso Study)	(i) N = 4,160 postmenopausal women: 3,947 without ( 1,997 never, 915 former, and 1,035 current smokers) and 213 with diabetes ( 128 never-smokers, 47 former smokers, and 38 current smokers) (ii) The range of age was 55-74 years (iii) All participants were females	(i) To investigate whether smoking is associated with risk for nonvertebral fractures with special emphasis on diabetes mellitus (DM)	(i) There were 1,015 without and 66 with diabetes that sustained a new nonvertebral fracture. (ii) There was a significant relationship between smoking status and increased risk of fracture in women with (P = 0.02) and without diabetes (P < 0.001), and there was a strong interaction between smoking status and diabetes on fracture risk (P = 0.004). (iii) Compared to the diabetic women who were never-smokers, women with diabetes who were current smokers had a higher risk of nonvertebral fractures (OR = 3.47, 95%CI: 1.82-6.62, P = 0.001).

Jutberger et al., 2010 [62]	Prospective cohort study (Sample from Mr OS Sweden)	(i) N = 3,003: 1,058 never-smokers, 1,693 former smokers, 252 current smokers (ii) The range of age was 69-80 years (iii) All participants were males	(i) To examine the relationships between smoking and BMD and the incidence and prevalence of fracture	(i) The baseline data show that current smokers had a lower total hip and lumbar BMD (P < 0.001). (ii) Smokers had a high risk for vertebral fractures (OR = 3.18, 95%Cl: 1.88-5.36). (iii) Current smokers had an increased risk of all new (HR = 1.76, 95% Cl: 1.19-2.61) and osteoporotic fractures (HR = 2.14, 95%Cl: 1.18-3.88)

Kaiser et al., 2012 [63]	Retrospective cohort study	(i) N = 51patients with long bone fracture from 3 case-control studies (ii) The mean of age was 59.3 with a range of 17-90 years (iii) 22 were males, and 29 were females	(i) To investigate whether smoking, alcohol consumption, diabetes mellitus, and demographic factors (age, sex) influence the expression of Transforming Growth Factor-Beta1 after fracture of long bones (TGF-beta 1) which may act as predictor for delayed bone healing	(i) At 8 weeks after surgery, compared to nonsmokers, smokers had significantly lower TFG-beta 1 serum concentrations. (ii) At 24 weeks after surgery, compared to females, male patients had significantly higher TFG-beta 1 serum concentrations. Also, younger patients had significantly higher concentrations compared to older patients. (iii) At 6 weeks after surgery, TGF-beta 1 concentrations were significantly higher in patients without diabetes compared to those with diabetes. Also, patients with chronic alcohol abuse had significantly higher concentrations compared to those patients without chronic alcohol abuse. (iv) The concentration of TGF-beta 1 serum vary based on smoking status, alcohol abuse, diabetes mellitus, age, and gender

Krannitz et al., 2007 [64]	Retrospective cohort study	(i) N = 52: 26 underwent surgical and 26 underwent conservative treatment for supination-external rotation II fibular fractures: 27 smokes, and 15 patient who bore weight early (ii) The range of age was 15-85 (iii) 24 were males and 26 were females	(i) To compare the time interval of radiographic healing in both the surgically and conservatively managed supination-external rotation II fibular fractures smoking and premature weight-bearing population	(i) Conservative group had higher mean time for cortical bridging than that of the surgical group (89.38 days versus 48.69). (ii) Smokers and patients with prematurely bore weight in both groups of surgical and conservative groups had significantly longer mean times of healing.

Moghaddam et al., 2010 [65]	Prospective cohort study	(i) N = 248 patient underwent surgical treatment for long bone: then final analyses were included: 14 (7 smokers, 7 nonsmokers) patients with delayed healing were compared with 14 (7 smokers, 7 nonsmokers) with normal healing (ii) Average of age was 48.0 in the nonunion and 47.4 years in the union group (iii) 187 were males, and 61 were females	(i) To compare the differences of TGF-beta 1 between cigarette smokers and non-cigarette smokers whether they have timely or delayed healing fracture	(i) At 4 weeks after surgery, in patients with normal fracture healing, compared to nonsmokers, smokers had lower levels of TGF-beta 1(50 ng/ml versus 70 ng/ml, P = 0.007). Also, at this time similar trends were observed in patients with delayed healing, and smokers had lower TGF-b1 than that of nonsmokers (38 ng/ml versus 47 ng/ml, P = 0.021). (ii) The serum concentration of TGF-beta 1 reached a plateau in all groups from 6 to 12 weeks after surgery, with a slight decrease observed in the final measurement at 1 year after surgery

Moghaddam et al., 2011 [66]	Prospective cohort study	(i) N = 85: 39 never-smokers, 45 smokers (former or current) (ii) The average of age 44 ± 15 with a range of 18-84 years (iii) 61 were males, and 24 were females	(i) To investigate the effects of cigarette smoking on the clinical, functional, psychosocial and occupational outcomes after isolated lower-leg fracture	(i) Compared to never-smoking, smoking had significantly increased risk for delayed union or nonunion (P = 0.0007) and increased time required for fracture healing (P = 0.003), but nonsmoking significantly increased time out of work (P = 0.1177). (ii) Smokers had 3-18-fold higher risk of impaired bone healing. (iii) The mean scores for mental scores and physical-function in the SF-36 were similar in both groups of smokers and nonsmokers

Oyen et al., 2015 [67]	Prospective cohort study (Sample from large community-based Hordaland Health Study)	(i) N = 5,315 with BMD reading, and 3, 310 with hip fracture (ii) The range of age for patient with BMD was 46-49 for middle age cohort group and 71-74 for old cohort, while it was 71-74 for patients with hip fracture (iii) For patient with BMD (2,245 males, 3,070 were females) and for patients with hip fracture (1,455 males, and 1,855 females)	(i) To investigate the associations of plasma dimethylglycine (DMG) with BMD and with hip fracture. Also, to investigate influence of nicotine on these associations	(i) Compared to participants with highest DMG tertile, participants with lowest tertile had a higher risk to have low BMD (OR = 1.68, 95%CI: 1.43-1.99). (ii) Compared to the subjects not exposed to nicotine, subjects who were exposed to nicotine had stronger association between BMD and DMG (OR = 2.31, 95%CI: 1.73-3.07 for exposed, and OR = 1.43, 95%CI: 1.16-1.75 for unexposed, P interaction = 0.008). (iii) There was an increased risk of hip fracture in older patient with low level of plasma DMG (HR = 1.70, 95 % CI: 1.28-2.26), and such risk was high in women exposed to nicotine (HR = 3.41, 95 %CI: 1.40-8.28).

Rodrigues et al., 2012 [68]	Case-control study	(i) N = 92: case: 40 hip fragility fractures (4 were smokers), control: 52 osteoarthritis patients (10 were smokers) (ii) The average of age (Mean ± SD): 80.0 ± 6.9 for hip fracture, and 69.0 ± 8.4 for osteoarthritis (iii) 34 were males, and 58 were females	(i) To assess differences, adjusted for clinical risk factors (CRFs), between bone macrostructural (trabecular mechanical behavior, femoral neck BMD, and femoral head diameter) and to determine if CRF parameter could predict mechanical behavior of the trabecular bone in hip fragility fractures	(i) Compared to osteoarthritis individuals, patients with hip fragility fracture had significantly lower trabecular bone stiffness (P = 0.042) and there were no differences in structural parameter between the groups. (ii) Both smoking (beta = -0.403; P = 0.018) and female gender (beta = -0.416; P = 0.008) were independently associated with lower stiffness in patients with hip fragility fracture. (iii) There was an inverse association between smoking and trabecular strength (beta = -0.323; P = 0.045) and toughness (beta = -0.403; P = 0.018). (iv) The 10-year risk of major (r = -0.550; P = 0.012) and hip fracture (r = -0.513; P = 0.021) was strongly correlated with femoral neck BMD but not with mechanical performance

Schroter et al., 2015 [69]	Prospective cohort study	(i) N = 70: 47 nonsmokers, and 23 smokers (ii) The average of age (Mean ± SD): 46 ± 8 years with a range of 19-56 (iii) 47 were males, and 23 were females	(i) To observe the rate of gap filling after open wedge high tibial osteotomy (HTO) that was conducted without bone graft and to study the influence of several factors (e.g., smoking, hinge fracture, and weight bearing) on the process of gap filling	(i) During all time of follow-up (6 and 12 weeks and 6, 12 and 18 months), there was a delay in the osteotomy gap filling rate between smokers but differences were not significant. However, early full weight bearing had no effect on the gap filling rate. (ii) A fracture of the lateral hinge was found in 39% (27) of the patients. The highest rate was type I fracture (14%), followed by type II fracture (13%), and type III fracture (6%). (iii) In patient with intact hinge (lateral), the highest rate in gap filling was observed in period between 12 weeks and 6 months after the surgery

Taes et al., 2010 [70]	Cross-sectional population-based study	(i) N = 677: RA: 397 never-smokers, 125 former smokers, 155 current smokers (ii) The average of age (Mean ± SD): 34.5 ± 5.5 never-smoker, 33.4 ±5.5 former smokers, and 35.7 ± 5.5 current smokers (iii) All participants were males	(i) To investigate smoking habits in relation to areal and volumetric bone parameters and fracture prevalence in young, healthy males	(i) Compared to the never-smokers, current and early smoker's had higher prevalence of fractures (P < 0.05) with an OR of 1.96 (95%CI: 1.18-3.24). (ii) The findings at tibia found that current smoking was associated with a decreased cortical thickness (beta = -0.034 ± 0.01, P = 0.020) and greater endosteal circumference (beta = 0.027 ± 0.009, P = 0.016). (iii) Smoking at early age (≤ 16 years) associated with higher fracture risk and lesser areal BMD. (iv) The interaction between current smoking and free estradiol was observed in model used to predict cortical thickness (beta = 0.29 ± 0.11, P = 0.01).

Thorin et al., 2016 [71]	Prospective cohort study	(i) N = 1,033: at baseline 679 nonsmokers, 209 former smokers, 145 current smokers (ii) All patients were included at 75 and followed for 10 years (iii) All participants were females	(i) To examine effects of smoking and smoking cessation on fracture risk	(i) Compared to nonsmokers, both former and current smokers had an increased risk for any fracture (HR = 1.30; 95%CI: 1.03-1.66, and HR = 1.32; 95%CI: 1.01-1.73, respectively) and any osteoporotic fracture (HR = 1.31; 95%CI: 1.01-1.70 and HR = 1.49; 95%CI: 1.11-1.98, respectively) (ii) Compared to nonsmokers, former smokers had an increased risk for proximal humerus fractures (HR = 2.23; 95%CI: 1.35-3.70), and current smokers had an increased risk for vertebral fractures (HR = 2.30; 95%CI: 1.57-3.38) (iii) Compared to current smokers, former smokers had a decreased risk for vertebral fractures, but not for other types of fractures

Wilsgaard et al., 2011 [72]	Prospective cohort study [sample from Tromso Study]	(i) N = 10,977 (ii) The range of age was 20-54 years at baseline (iii) 5,549 were males, and 5,428 were females	(i) To examine the association between BMI change and fracture risk in both genders and to examine smoking effect on this association.	(i) Ten years of follow-up resulted in 1,135 fractures. (ii) In nonsmokers but not in smokers, reduction in BMI was associated with increased nonvertebral fracture. (iii) In nonsmokers of males and females, the hazard ratio per 10 years for one unit decrease in BMI was 1.79 (95%CI: 1.17-2.75) and 1.60 (95%CI: 1.28-1.99), respectively. (iv) There was an inverse association between weight gain and risk of fracture in females who were nonsmokers.

***1.C: Tobacco Smoking and Alveolar Bone (4 Articles)***				

Campos et al., 2015 [73]	Case-control cross-sectional study	(i) 19 heavy smokers, and 19 nonsmokers (never smoked) (ii) Average age in years: smoker = 52.0 ±5.7, nonsmokers = 42.0 ± 12.5)	(i) To study the effect of smoking on the expression of alveolar bone-related markers	(i) Smoking was found to negatively affect the expression of bone sialoprotein (BSP) and osteocalcin (OCN) mRNA (P< .05) and it positively altered the expression of type I collagen (COL-I) (P< .05); however, smoking was not statistically correlated with the expression of mRNA for tumor necrosis factor-alpha (TNF-*α*), transforming growth factor-beta (TGF-*β*), and osteoprotegerin (OPG).

Levin L & Levin J, 2010 [74]	Cross-sectional study	(i) N = 480: 372 were nonsmokers, 108 were smokers (ii) Mean age of 19.2 ± 1 year with a range of 18-29 years. (iii) All participants were males.	(i) To investigate the effect of smoking on the alveolar bone height and density.	(i) Compared to the nonsmokers, smokers had significantly lower bone mineral density values (P ≤ 0.002). (ii) Smokers were found to have a significantly greater distance of cemento-enamel-junction (CEJ) to the alveolar bone crest (P < 0.0001). (iii) The distance from the CEJ to the alveolar bone crest was found to be positively associated with the number of cigarettes/days (P < 0.001) and the years of smoking (P = 0.03).

Mesa et al., 2014 [75]	Case-control study	(i) N = 315: 242 nonsmokers, 71 smokers (>10 cigarettes /day), and 2 unknown (ii) Average of age was 36.6 ± 5.3 with 16.2% in range of 21-30 years, and 83.8% in range of 31-46 years. (iii) Caucasian	(i) To examine the relationships between tobacco consumption with alveolar crest height (ACH) loss and mandibular bone mass	(i) ACH loss was greater in older patients with P = 0.012 and patients had fewer mandibular teeth (P < 0.001). The relationship between ACH loss and tobacco consumption was close to significant (P = 0.079). (ii) There were significant associations between ACH and the number of mandibular teeth (P < 0.001) and tobacco consumption (P = 0.048). (iii) There were a significant association between alveolar and basal bone densities and number of mandibular teeth (P = 0.012) and cortical width (P = 0.030)

Rosa et al., 2008 [76]	Experimental: Parallel-arm Prospective design with 4 points: preexperimental (baseline/0 day), 180, 365, and 545 days	(i) N = 81: 42 smokers, and 39 never smoked (ii) The mean of age for smokers and nonsmokers 20.5 years with a range of 18-25 years	(i) To evaluate the effect of smoking on the alveolar bone of young adult after involvement in rigorous dental hygiene program (DHP)	(i) The bone height measurement (BHM) indicated a significantly lower mean alveolar bone height in the smokers (P < 0.01). (ii) The smokers showed significantly lower computer-assisted densitometric image analysis (CADIA) values across the 4 points of the study and it was significantly different at baseline and 545 days (P < 0.01). (iii) With follow-up, CADIA values decreased in smokers and reached significant level by Day 545 (P < 0.05). (iv) The mean percentage of sites with decreased bone density had increased by Days 365 (P < 0.05) and 545 (P < 0.01).

***1.D: Tobacco Smoking and Periodontitis*** ***(34 Articles)***				

Adler et al., 2008 [77]	Retrospective case-control study	(i) N = 293: 155 nonsmokers, 138 smokers (ii) The mean of age was 60.1, with a range of 57-64 years (iii) 152 were males, and 141 were females	(i) To investigate the differences in periodontal probing pocket depth profile between smokers and nonsmokers as well as within the smoking group	(i) Compared to nonsmokers, smokers had significantly deeper periodontal pockets. (ii) There was a significant association between smoking and the percentage share of palatal periodontal pockets ≥ 6 mm

Al-Habashneh et al., 2009 [78]	Cross-sectional study	(i) N = 560: 370 nonsmokers, 190 smokers (ii) The range of age was 16-35 (iii) 268 were males, and 292 were females	(i) To assess the relationships between aggressive periodontitis (AgP), caries and smoking	(i) The prevalence of smoking was higher in subjects with chronic periodontitis (CP) (44.2%) than in subjects with chronic gingivitis (CG) and aggressive periodontitis (AgP) (44.2%, 27.4%, 29.9%, respectively) (ii) In AgP group only, the mean plaque scores were significantly higher for smoker than nonsmoker (P = 0.04) (iii) The CAL was significantly higher in smokers than in nonsmokers of CP and AgP groups (P = 0.04, 0.01 respectively)

Bukmir et al., 2016 [79]	Cross-sectional study	(i) N = 259: 151 never-smokers, 108 current smokers (ii) The average of age (Mean ± SD): 41.9 ± 16.2 never-smokers, 38.8 ± 13.6 years current smokers (iii) 88 were males, and 177 were females	(i) To investigate the difference in the periapical status of endodontically treated and untreated teeth in current smokers and never-smokers	(i) Compared with never-smokers, current smokers had higher fraction of teeth with apical periodontitis (AP) (0.13 versus 0.10; P = 0.025). (ii) Smokers were 16.4 times more likely to have AP than nonsmoker (95% CI: 5.7- 47.7; P < 0.001), and male person was 3.1 more likely to have AP than a female (95%CI: 1.1-8.9; P = 0.039). The likelihood of AP increases with age. (iii) While controlling for age, gender, and overall number of teeth, smokers will have two teeth with AP more than nonsmokers

Chantarangsu et al., 2016 [80]	Cross-sectional study	(i) N = 1,460: 873 nonsmokers, 337 former, and 250 current smokers (ii) The average of age (Mean ± SD): 47.3 ± 4.4, with a range of 30-66 years (iii) 1,044 were males, and 416 were females	(i) To investigate whether susceptibility to chronic periodontitis (CP) in a Thai population is associated with vitamin D receptor (VDR) polymorphisms and smoking	(i) The CC+CT genotypes of FokI polymorphism were associated with severe CP (OR = 1.9, 95%CI: 1.3 - 2.8). However, other VDR polymorphisms (BsmI, ApaI, and TaqI) were not significantly associated with CP. (ii) The combination of smoking and having genotype positive increased OR to 9.6 (95%CI: 4.5 - 20.4), and such joint effect was 3.7 times (95% CI: 1.2 to 11.1) greater than the sum of their individual effects

Dastoor et al., 2007 [81]	Randomized, placebo-controlled, double-masked clinical trial	(i) N = 30 heavy smokers (> 1 pack/day) with moderate-severe CP randomized to: Test [surgery + 3 days 500 mg AZM], Control [surgery + 3 days placebo] (ii) The average of age (Mean ± SD): 49.4 ± 7.81 (iii) 17 were males, and 13 were females	(i) To evaluate effects of systemic azithromycin (AZM) as an adjunct treatment in combination with periodontal pocket reduction surgery in the treatment of CP in smokers	(i) Compared with control group: (a) at 2 weeks, the test group had significantly less gingival index better; (b) at 1 month, the test group had significantly better wound healing indices and, (c) at 3 months, the test group had sustained reduction in red-complex bacteria. (ii) The AZM therapy in combination with pocket reduction surgery did not significantly enhance PD reduction or CAL gain.

de Heens et al., 2009 [82]	Cross-sectional study	(i) N = 54: 30 periodontal treated patients (12 smokers and 18 nonsmokers), and 24 healthy subject control (12 smokers, and 12 nonsmokers) (ii) The range of age was 25-65 years (iii) 21 were males (11 in patient, 10 in control), and 32 were females (18 in patient, 14 control)	(i) To investigate the T lymphocytic cytokine production (Th1 and Th2) in smokers and nonsmokers with or without periodontitis	(i) Smokers had more lymphocyte and higher levels of both IFN-*γ* and IL-13, regardless of being periodontal patient. However, in a multivariate analysis, only higher level of IL-13 was strongly explained by smoking (P < 0.001)

Eder et al., 2012 [83]	Cross-sectional study	(i) N = 45: 14 nonsmokers, 12 former smokers, and 19 current smokers (ii) The average of age (Mean ± SD): 47 ± 15 (iii) 29 were males, and 16 were females	(i) To investigate the conversion rate of C-14 labelled arachidonic acid (C-14-AA), the lipoxygenases (LOX) products and the endogenous synthesis of eicosanoids and isoprostanes (IPs) in extracted periapical granuloma and to assess if there are markers for bone destruction and to study the influence of cigarette smoking.	(i) Smoking increases significantly the synthesis of IPs and LOX-metabolites in granuloma. (ii) Smoking may have contributed to significant differences in eicosanoids profile qualitatively and quantitatively, Ips, and the conversion rate of C-14-AA independent of the size of the granuloma.

Edman et al., 2015 [84]	Cross-sectional study [sample obtained from 4 epidemiological study in Sweden ]	(i) N = 787-1,133 (ii) The range of age was 35-85 years (iii) Random sample	(i) To investigate 30-year time (1983-2013) trends to classify the prevalence and severity in alveolar bone loss (ABL) and to investigate whether ABL influenced by tobacco and socioeconomic factors	(i) The prevalence of severe ABL was not changed from 1983 (7%) to 2013 (6%). However, the prevalence of moderate ABL was decreased from 45% in 1983 to 16% in 2008 but increased to 33% in 2013 (P < 0.05). (ii) Calculus visible on radiograph was significantly increased from 2008 to 2013 (22% to 32%, P < 0.05). (iii) Smoking was the strongest factor associated with ABL; however, socioeconomic factors had limited influence on the severity of ABL.

Eren et al., 2015 [85]	Cross-sectional study	(i) N = 32 periodontally healthy subjects: 16 nonsmokers, 16 smokers (ii) The average of age (Mean ± SD): 22.7 ± 1.9 nonsmokers, 22.4 ± 1.3 smokers (iii) 18 were males, and 14 were females	(i) To investigate gingival crevicular fluid (GCF) levels of matrix metalloproteinase (MMP-1, and MMP-8), transforming growth factor- (TGF-) beta 1, platelet-derived growth factor (PDGF)-AB, and vascular endothelial growth factor (VEGF) in smoking versus nonsmoking periodontally healthy subjects	(i) Smokers had significantly lower total amount of PDGF-AB than nonsmokers (P = 0.014). However, total amounts of GCF, MMP-1, MMP-8, TGF-beta 1, and VEGF levels were similar in both groups

Fisher et al., 2008 [86]	A 3-year longitudinal study	(i) N= 81 had available longitudinal measurement: 65 nonsmokers, 16 smokers (ii) The average of age (Mean ± SD): 59 ± 14 nonsmokers, 54 ± 6 smokers (iii) 40 were males (8 smokers, 8 nonsmokers), and 41 were females (8 smokers, 33 nonsmokers)	(i) To assess longitudinal disease progression longitudinally in subjects with CP (smokers and nonsmokers) who are undergoing periodontal maintenance therapy every 3 to 4 months	(i) There were no differences in inflammatory indices at baseline or over time [plaque index (PI), bleeding on probing (BOP)]; both P > 0.05) between smokers and nonsmokers. (ii) Also, disease progression parameter (changes in prevalence and proportion of progressing sites and as mean clinical attachment loss (CAL), probing depth (PD), and tooth loss; all P > 0.05)

Ghallab & Shaker, 2010 [87]	Cross-sectional pilot study	(i) N = 44: 22 patients with CP (11 nonsmokers, 11 smokers) and 22 periodontally healthy controls (11 nonsmokers, 11 smokers) (ii) The range of age was 32-51 years in periodontal healthy controls and it was 32-55 in CP subjects (iii) 23 were males (13 healthy, 11 CP); 21 were females (9 healthy, 12 CP)	(i) To investigate the salivary sCD44 profiles of smokers and nonsmokers with and without CP in response to scaling and root planing (SRP)	(i) In both groups of CP and healthy subjects, baseline salivary sCD44 profiles were significantly higher in smokers than that of nonsmokers (P < 0.001) with the highest levels recorded in smokers within the CP group. (ii) After treatment, there was a significant decline in salivary sCD44 levels for smokers and nonsmokers in the CP group (P < 0.01); however, the difference was not significant.

Gurlek et al., 2009 [88]	Cross-sectional pilot study	(i) N = 67: 34 smokers, 22 nonsmokers, and 11 former smokers (ii) The range of age was 39-56 (iii) 44 were males; 23 were females	(i) To examine whether smoker patients with inflammatory periodontal disease had salivary concentrations of C-telopeptide pyridinoline cross-links of type I collagen (ICTP) and osteocalcin (OC) different than that of nonsmokers and/or ex-smokers counterparts	(i) Smoker had significantly higher plaque index than nonsmokers (P < 0.05). (ii) Compared to ex-smokers, smokers had significantly lower bleeding on probing (P < 0.05). (iii) Salivary ICTP levels were similar among the study groups (P > 0.05). Salivary ICTP levels were correlated negatively with number of teeth (P < 0.05) and positively with bleeding on probing (P < 0.01). (iv) OC levels in smoker group were significantly lower than nonsmoker's ex-smokers groups (P < 0.001). Salivary OC levels correlated negatively with years smoked (P < 0.01)

Heikkinen et al., 2008 [89]	Cross-sectional study	(i) N = 501: 329 nonsmokers, 127 smokers, 45 former smokers (ii) The range of age was 15-16 (iii) 258 were males, 243 were females	(i) To investigate the effect of smoking on periodontal health in teenagers and possible differences between genders	(i) Boys and girls who smoked had higher root calculus values than nonsmokers (P < 0.001). (ii) Compared to nonsmokers, smoking boys and girls also had more periodontal pockets ≥ 4 (score for boys = 4.6, 95%CI: 2.2 to 9.1, the score for girls = 5.4, 95%Cl: 1.1 to 23.2; P < 0.001)

Hugoson & Rolandsson, 2011 [90]	Cross-sectional epidemiological study [sample obtained from 3 cross-sectional epidemiological study in Sweden 20 years]	(i) N = 1,591: 1,142 nontobacco users, 345 cigarettes smokers, and 104 snus users (ii) The average of age 9Mean ± SD): 45.9 ± 16.9 nontobacco, 40.9 ± 15.8 smokers, and 34.9 ± 14.3 snus users (iii) 691 were males (526 nontobacco users, 156 smokers, 99 snus users), and 810 were females (616 nontobacco users, 189 smokers, 5 snus users) (iv) 90% were Caucasian (v) Stratified random sample: based on age	(i) To compare periodontal health for tobacco users [current cigarette smokers and the users of Swedish moist snuff (snus)] compared with nontobacco users	(i) Compared to nontobacco users, cigarette smokers had statistically significant less gingivitis, a higher frequency of periodontal probing pocket ≥ 4 mm, and a higher incidence of severe periodontitis. (ii) Use of snus was not significantly associated with gingivitis, frequency of probing pocket depth ≥ 4 mm, and periodontal disease.

Javed et al., 2015 [91]	Cross-sectional study	(i) N = 95: 45 with prediabetes (16 never-smokers, 29 smokers) and 50 healthy controls (23 never-smokers, 27 smokers) (ii) The average of age was 43.1 and the range of age was 36-55 years (iii) All participants were males	(i) To assess the periodontal status and whole salivary IL-1 beta and IL-6 levels among smokers and never-smokers with and without prediabetes	(i) Among prediabetes patients, periodontal parameters and whole salivary IL-1 beta and IL-6 levels were similar among smokers and never-smokers. However, among controls, periodontal parameters and whole salivary IL-1 beta and IL-6 levels were higher among smokers than never-smokers (P < 0.05)

Javed et al., 2016 [92]	Cross-sectional study	(i) N = 200: 50 WPs, 50 CSs, and 100 control (ii) The average of age (Mean ± SD): 48.5 ± 6.2 WPs, 50.1 ± 3.5 CSs, 46.5 ± 4.2 control (iii) All participants were males	(i) To compare the clinical and radiographic periodontal status between habitual water-pipes (WPs) smokers and cigarette smokers (CSs)	(i) Compared to control, both WPs and CSs had significantly higher number of missing teeth (P < 0.0001), plaque index (P < 0.0001), clinical attachment loss (P < 0.0001), probing depth ≥ 4 mm (P < 0.0001), and marginal bone loss (P < 0.0001). (ii) Bleeding on probing was significantly higher in controls than in WPs (P < 0.0001) and CSs (P < 0.0001). (iii) There was no significant difference in all investigated periodontal parameters between WPs and CSs

Javed et al., 2015 [93]	Cross-sectional study	(i) N = 100: 50 with T2DM (25 nonsmokers, 25 smokers) and 50 controls (25 never smokes, 25 smokers) (ii) The average of age (Mean ± SD): 52.6 ± 3.8 (iii) All participants were males	(i) To assess periodontal inflammatory conditions among smokers and never-smokers with and without type 2 diabetes mellitus (T2DM)	(i) Among patients with T2DM, periodontal parameters were similar among smokers and never-smokers with T2DM. (ii) Among controls, compared to never-smokers, smokers had significantly higher plaque index (P < 0.05), clinical attachment loss (P < 0.05), probing depth ≥ 4 mm (P < 0.05), and marginal bone loss (P < 0.05). (iii) Compared with smokers and never-smokers in control group, smokers with T2DM had poorer periodontal status (P < 0.05).

Khan et al., 2016 [94]	Cross-sectional study	(i) N = 443: 199 nonsmokers, 244 smokers [123 heavy (≥ 5 cigarettes/day), 121 light/moderate smokers(< 5 cigarettes/day) ] (ii) The average of age (Mean ± SD): 44.3 ± 6.5 (iii) 287 were males, and were 156 females	(i) To determine the prevalence and dose-response relationship of CP among smokers in Pakistan.	(i) The overall prevalence of CP among smokers was 81.6%. (ii) Compared to moderate/light smokers, heavy smokers had significantly higher prevalence (P < 0.001) and severity (P < 0.001) of periodontitis. (iii) Smokers had 3.5 times greater risk of chronic periodontitis when compared with nonsmokers (P < 0.001).

Khemiss et al., 2016 [95]	Cross-sectional study	(i) N = 120: 60 ENS (7 ± 3 narghile-years), 60 ECS (8 ± 3 pack-years) (ii) The average of age was 20-35 years (iii) All participants were males	(i) To compare the periodontal bone height (PBH) of exclusive narghile smokers (ENS) with that of exclusive cigarette smokers (ECS)	(i) Compared with the ECS group, ENS group had a significantly higher plaque index (1.54 ± 0.70 versus 1.84 ± 0.73, P = 0.02). (ii) Both groups of ECS and ENS had similar means of periodontal bone height (P = 0.56) and tooth brushing frequencies (P = 0.43) and had similar bone loss frequencies (P = 0.63)

Kibayashi et al., 2007 [96]	Prospective cohort (longitudinal) study	(i) N = 219 out of 256 examined at 1993 baseline completed PD measurements at 2003: 111 nonsmokers (never and ever), and 108 current smokers (ii) The average of age was 18-63 years (iii) 193 were males, and 26 were females	(i) To examine the prospective association between smoking and PD progression and the effects of smoking on salivary biomarkers related to periodontitis	(i) There were significant associations between PD progression and current smoking (OR = 2.27, 95% CI: 1.02 - 4.28) and hours of sleep (OR = 2.05, 95%CI: 1.11- 3.78). (ii) 38.5% of PD progression was attributable to current smoking. Furthermore, pack-years of smoking showed a dose-response relationship with disease progression. (iii) Compared to noncurrent smokers, current smokers had significantly higher level of salivary markers: prostaglandin E-2, lactoferrin, albumin, aspartate aminotransferase, lactate dehydrogenase, and alkaline phosphatase. (iv) The proportion of six identified pathogens did not differ between current and noncurrent smokers.

Kiss et al., 2010 [97]	Cross-sectional pilot study	(i) N = 44: 20 nonsmokers ([10 without periodontitis, 9 with chronic (CP), 1 with aggressive periodontitis(AP)], 24 smokers [4 without periodontitis, 16 with CP, and 4 with AP ] (ii) The average of age (Mean ± SD): 54.7 ± 15.6 nonsmokers, 50.2 ± 6.9 smokers (iii) 18 were males, and 14 were females	(i) To assess the differences of salivary calcium concentration between smokers and nonsmokers with or without periodontitis	(i) Compared to nonsmokers, smokers had significantly higher level of salivary calcium (57.76 *μ*g/mL ± 18.8 versus 44.6 *μ*g/mL ± 7.8, p < 0.05). (ii) Smoker's periodontal examination showed significantly greater bone loss, a deeper mean PD, and a higher amount of calculus (P < 0 .05). However, there were no statistically significant differences between the smokers and the nonsmokers regarding the plaque and bleeding indices

Koss et al., 2016 [98]	Cross-sectional study	(i) N = 55: 27 generalized aggressive periodontitis (14 nonsmokers, 13 smokers), 28 of healthy control group (14 nonsmokers, 14 smokers) (ii) The average of age (Mean ± SD): 26.9 ± 4.3 aggressive periodontitis, and 25.8 ± 2.9 years for health control (iii) 30 were males, and 25 were females	(i) To investigate the effect of smoking on antimicrobials and destructive proteins in the saliva of patients with generalized aggressive periodontal disease	(i) Level of sIgA was significantly differed between smokers with periodontal disease and healthy control subjects. (ii) The level of peroxidase was significantly differed between nonsmokers with periodontal disease and healthy control subjects. (iii) Collagenase activity was higher in smokers and nonsmokers with periodontal disease, and tobacco use was found to increase collagenase activity in healthy subjects

Lappin et al., 2007 [99]	Cross-sectional study	(i) N = 70: 35 nonsmokers, and 35 smokers (ii) The average of age (Mean, Range): 43.0 (40.0 -51.5) nonsmokers, 43.0 (41.0 - 50.5) for smokers (iii) 46 were males, and 24 were females	(i) To compare serum levels RANKL and OPG in age- and sex-matched groups of smokers and nonsmokers with identical levels of periodontal disease	(i) Compared to nonsmokers, smokers had significantly lower median serum level of OPG (23.76 pM versus 59.28 pM, P = 0.0006) but not for RANKL (41.47pM versus 48.23 pM, P = 0.0942). (ii) Tobacco consumption had statistically significant negative correlation with the concentrations of OPG.

Needleman et al., 2007 [100]	Randomized controlled trial	(i) N = 34: 16 test patients (LDD), 18 controls (Placebo) (ii) The range of age was 32-58 years at baseline	(i) The aim of this study was to investigate the effect of low-dose doxycycline (LDD) in smokers on nonsurgical periodontal therapy after 6 months' follow-up	(i) The velocity of change was statistically greater for the test group for clinical attachment level -0.19 mm/month (95% CI: -0.34 to 0.04; P = 0.012) and probing depth 0.30 mm/month (95%C: -0.42 to -0.17; P < 0.001). However, absolute changes in clinical or biochemical markers were not observed at 6 months, which indicates using LDD as an adjunct to nonsurgical periodontal therapy in smokers

Nile et al., 2013 [101]	Case-controlled cross-sectional study	(i) N = 200: 101 nonsmokers with periodontitis, 55 smokers with periodontitis, 27 healthy nonsmokers, 17 healthy smokers (ii) The range of age was 41-55 years for subjects with periodontitis, and 28-40 for healthy subjects (iii) There were an equal number of males and females	(i) To investigate plasma concentrations of TNF (TNSF1A), (sRANKL/ TNFSF11), a proliferation-inducing ligand (APRIL/TNFSF13), B-cell activating factor (BAFF/TNFSF13B and OPG/TNFRSF11B) in smokers and nonsmokers with and without CP	(i) Compared to healthy subjects, periodontitis patients had significantly higher plasma sRANKL, TNF, APRIL, and BAFF and lower OPG (P < 0.01). (ii) Smokers with periodontitis had significantly greater level of TNF (P = 0.011) and sRANKL (P = 0.001) and significantly lower level of OPG (P = 0.001), whereas APRIL or BAFF were little changed. (iii) Plasma APRIL, BAFF, sRANKL, and TNF correlated with PD and CAL

Ozcaka et al., 2011 [102]	Cross-sectional study	(i) N = 69: 33 with CP (25 nonsmokers, 8 smokers), 36 healthy control group (25 nonsmokers, 11 smokers) (ii) The range of age was 37-67 years for group of CP, and 35-64 for healthy control group (iii) 40 were males (23 CP, 17 control), and 29 were females (10 CP, 19 control)	(i) To investigate whether smoker CP patients exhibit different salivary concentrations of ICTP and OC compared to the nonsmoker counterparts	(i) Periodontal measurements were significantly different between the two groups of healthy control and group of CP (P < 0.001). However, these measurements were not differed between smoker and nonsmokers of CP group (P > 0.05). (ii) Compared to health control, patients with chronic periodontitis exhibited significantly higher salivary OC (P < 0.05). (iii) Within the group of CP, smoker had lower salivary OC levels than nonsmoker (P < 0.001). (iv) Within the healthy control group, smokers revealed higher log ICTP levels than nonsmoker (P < 0.001). (v) Within the subgroups of nonsmokers, nonsmoker chronic periodontitis exhibited higher log ICTP levels than nonsmoker (P < 0.05).

Ozcaka et al., 2010 [103]	Cross-sectional study	(i) N = 86: 44 with CP (31 nonsmokers, 13 smokers), 42 healthy control group (29 nonsmokers, 13 smokers) (ii) The range of age was 35-65 years for group of CP, and 33-57 for healthy control group (iii) 43 were males (23 CP, 20 control), and 43 were females (21 CP, 22 control)	(i) To evaluate plasma levels sRANKL and OPG in smoker versus nonsmoker CP patients	(i) All periodontal measurements were significantly different between the two groups of healthy control and group of CP (P < 0.05). However, these measurements were not differed between smoker and nonsmokers of CP group (P > 0.05). (ii) Chronic periodontitis smokers exhibited significantly lower plasma OPG concentrations (P = 0.007) and higher sRANKL/OPG ratio (P = 0.01) than healthy control smokers

Rao et al., 2013 [104]	Randomized controlled trial	(i) N = 35 out of 40 CP completed measurements: 17 SRP plus SMV 1.2%, and 18 SRP plus placebo	(i) To investigate the effectiveness of Simvastatin (SMV) 1.2% in an indigenously prepared biodegradable controlled release gel as an adjunct to scaling and root planing (SRP) in the treatment of smokers with CP	(i) Compared to placebo group, SMV group had exhibited significantly higher means for probing depth reduction and attachment level gain at all visits (All P < 0.001). Furthermore, the mean percentage of bone fill was significantly higher in the SMV group than the placebo at all visits (All P < 0.001)

Rosa et al., 2014 [105]	Prospective cohort study	(i) N = 116: 61 continued for 24 months: 18 quit (Q), 32 continued smoking (NQ), 11 oscillated (O) (ii) The average of age was 48.2 ± 8.4, and the range was 25-66 years (iii) Around 40% were males, and 60% were females	(i) To assess the effect of smoking cessation on nonsurgical periodontal therapy (NSPT) in adult subjects with CP after 24 months' follow-up	(i) Compared to NQ, Q had significantly higher mean CAL gain in diseased sites and higher reduction in the proportion of sites with CAL ≥ 3 mm. In addition, Q presented significantly higher mean PD reduction relative to NQ (p ≤ 0.05).

Shiloah et al., 2014 [106]	Parallel double-blind randomized pilot study	(i) N = 24 smoker subjects: test group (T) received daily 325 mg of ASA, and control group (C) received placebo (ii) The average of age was 51.15 with a range of age was 34-70 years (iii) 14 were males, and 10 were females (iv) Around 50% of participants were white	(i) To determine the effect of aspirin (325 mg/day) on the clinical outcomes of scaling and root planing in adult smokers	(i) Between groups, there were no significant differences for the posttest mean differences for all periodontal measurements. (ii) Within groups, T group had more statistically significant differences between pretest and posttest scores than that of C group (P < 0.05; one-tailed)

Tang et al., 2009 [107]	Cross-sectional study	(i) N = 149 periodontitis patient: 58 never, 39 former, and 52 current smokers (ii) The range of age was 26-86 years (iii) 56 were males, and 93 were females	(i) To compare the levels of the sRANKL and OPG and their relative ratio in gingival crevicular fluid (GCF) among periodontitis patients with varying smoking histories	(i) There were no significant differences for sRANKL, OPG, and their relative ratio among never-smokers, former smokers, and current smokers. (ii) Compared to never-smokers, high pack-years group had significantly reduced OPG and subsequently increased sRANKL/OPG ratio (positively correlated with pack-year even after adjustment for age and status of current smoking)

Torrungruang et al., 2012 [108]	Cross-sectional study	(i) N = 1,463: 54: 477 nonsmokers, 714 former, 272 current smokers (ii) The range of age was 50-73 years (iii) Analysis only included males	(i) To investigate the effects of cigarette smoking on periodontal conditions in specific tooth regions of older Thai men over 50 years of age	(i) Compared with nonsmokers, smokers had deeper pockets and attachment loss, and the greatest differences were observed in the maxillary posterior palatal region.

Wan et al., 2009 [109]	Prospective cohort study	(i) N = 40: 20 nonsmokers, 20 smokers (ii) Average of age was 45.6 years (iii) All participants were males	(i) To investigate the factors predicting nonsurgical periodontal treatment responses using multilevel modeling	(i) Site-level variations contributed to 70-90% of the total variance. (ii) Three predictors (nonsmokers at subject level, anterior teeth at tooth-level, and site without plaque at site level) were significantly associated with a greater reduction in probing pocket depth over the 12-month study period (P < 0.05). However, there was no consistent predictor found for probing attachment level gain

Zee et al., 2016 [110]	Randomized controlled trial	(i) N = 25: 14 received mYJ, and 11 placebo (ii) The average of age (Mean ± SD): 46.3 ± 6.8 years (iii) All participants were males	(i) To evaluate the adjunctive use of modified YJ (mYJ) or YJ containing additional osteoblast-stimulating and inflammation-modulating Chinese medicinal herbs in the nonsurgical periodontal treatment of smokers with CP	(i) After 12-month follow-up, all of the periodontal parameters had improved, and no statistically significant differences were found between the control group and test group, except for the higher computer-assisted densitometry image analysis values (P = 0.025)

***1.E: Tobacco Smoking and Bone Implants (33 Articles)***				

Aglietta et al., 2011 [111]	Retrospective controlled analysis	(i) N = 40: divided into 4 groups with 10/group: 2 groups of periodontal compromised (PC) [10 turned and 10 screwed surfaces] and 2 groups of periodontal healthy (PH) [10 turned and 10 screwed surfaces] (ii) The mean of age in four groups was similar (≈51 years ± 3 years) (iii) 19 were males, and 21 were females	(i) To compare the 10-year marginal bone loss rates around implants supporting single-unit crowns in tobacco smokers with and without a history of treated periodontitis	(i) There were no significant differences in implant survival between the four groups and the rate ranged between 70% and 100% (P > 0.05). (ii) Compared with the implants placed in PH patients, implants placed in PC patients had significantly higher marginal bone loss independent of the system used (P < 0.05).

Anner et al., 2010 [112]	Prospective cohort study	(i) N = 475 patients with 1,626 implants: 412 nonsmokers, 63 smokers (ii) The average of age 9 (Mean ± SD): 51.96 ±11.98 (iii) 176 were males, and 299 were females	(i) To evaluate the factors associated with long-term dental implant survival	(i) 77 (4.7%) implants were lost in 58 (12.2%) subjects after a mean period of 24.71 ± 25.84 months. (ii) The two factors associated statistically with implant survival were smoking and attendance in a regular supportive periodontal program. However, diabetes mellitus patients who were treated for moderated to advance chronic periodontitis (CP) were not related to the dental implant survival

Ata-Ali et al., 2016 [113]	Prospective cross-sectional study	(i) N = 29 patients: 22 nonsmokers, and 7 smokers with 74 dental implants (ii) The average of age 9 (Mean ± SD): 63.6 ± 10.5 (iii) 12 were males, and 17 were females	(i) To investigate how heavy smoking influences the clinical, microbiological, and host-response characteristics in peri-implant sulcus fluid of patients with healthy dental implants	(i) Smoker had poorer peri-implant parameters but there were no significant differences found between the groups. (ii) Smoker's subgingival microbiota was composed of a greater number of periodontal pathogens. Also, smokers showed a greater expression of IL-1-*β*, IL-6, IL-10, and TNF-*α*, and IL-8; however, these differences were not statistically significant

Balshe et al., 2008 [114]	A retrospective chart review	(i) N = 1,498: 593 with 2,182 smooth-surface, and 905 patients with 2,425 rough-surface (ii) The mean of age (Mean ± SD): 51.3 ± 18.5 smooth -surface, 48.2 ± 17.8 rough-surface (iii) 637 males (271 smooth –surface, and 366 rough-surface), and 861 were females (322 smooth-surface, and 539 rough-surface	(i) To compare the long-term survival rates of smooth- and rough-surface dental implants among smokers and nonsmokers	(i) In implants with rough-surface, smoking was not associated with implant failure (HR = 0.8; 95%CI: 0.3-2.1; P = 0.68). In contrast, smoking was associated with implant failure in group with smooth-surface (HR = 3.1; 95% CI = 1.6-5.9; P < 0.001). (ii) Anatomic location had significant influence on the implant survival in smokers with smooth-surface implants (P = 0.0047), but it had no influence on implant survival in smokers with rough-surface implants (P = 0.45) and in nonsmokers with smooth-surface implants (P = 0.17).

Cakarer et al., 2014 [115]	Retrospective study	(i) N = 274 patients with 940 dental implants patients with 1,626 implants (ii) The mean of age was 50.20 ± 13.21, with a range of 19-84 years (iii) 109 were males, and 165 were females	(i) To evaluate the risk factors associated with the survival rate of the dental implants.	(i) After 5-year follow-up, 15 out of the 940 implants (1.5%) failed during the follow-up period which resulted in 98.5% survival rate. (ii) Smoking did not affect the survival rate of the implants (log-rank, 1.5; P = 0.219). (iii) The survival rate of the implants placed in the maxilla was lower than that of implants placed in the mandible (log-rank, 4.81; P = 0.028).

de Campos et al., 2015 [73]	Cross-sectional study with case & control	(i) N = 38: 19 nonsmokers, 19 smokers (ii) The average of age: 42.0 ± 12.5 nonsmokers, and 52.0 ± 5.7 smokers (iii) 20 were males, and 18 were females	(i) To evaluate the influence of smoking on the gene expression for protein related to bone metabolism in alveolar bone tissue from sites designed to receive dental implants	(i) Despite age, gender, and arch, smoking negatively affected mRNA expression of bone sialoprotein (BSP) and osteocalcin (OCN) and positively altered the expression of type I collagen (COL-I) (P < 0.05). However, smoking was not correlated with the expression of TNF-*α*, transforming growth factor-beta (TGF-*β*), and OPG (P > 0.05)

Cecchinato et al., 2015 [116]	Randomized controlled study	(i) N = 93 patients assigned to receive either a cylindrical or conical/cylindrical implant: 59 nonsmokers, and 35 smokers (ii) The mean of age was 51.0 ± 13.0, with a range of 19-80 years (iii) 48 were males, and 45 were females	(i) To study the peri-implant soft tissues response (recession and the papilla indexes) of patients treated with implants with two different configurations	(i) After the 3-year follow-up, a mean gain of 0.23 mm of gingival zenith (GZ) was measured. (ii) In analysis based on the stratification, no significant differences were found between the mean GZ scores of implants with TB ≤ 1 mm (thin buccal wall) and TB > 1 mm (thick buccal wall) at baseline and after 2 and 3 years of follow-up. (iii) The smoking did not seem to influence GZ changes over the follow-up period.

d'Avila, et al., 2010 [117]	Case-control study	(i) N = 7 edentulous patients received 2 implants with machined and sandblasted acid-etched surface (ii) The mean of age was 63 and the range of age was 37-68 years (iii) 4 were males, and 3 were females	(i) To evaluate the influence of implant surface topography of microimplants retrieved from posterior maxilla of smokers after 2 months of unloaded healing	(i) According to the histometric measurements, the mean bone-to-implant contact was significantly lower in machined implants than sandblasted acid-etched surfaces (10.40 ± 14.16% versus 22.19 ± 14.68%, P < 0.001). (ii) Findings support that the sandblasted acid-etched surface presented better results than the machined surface after a short healing time in smokers

D'Haese & De Bruyn, 2013 [118]	Clinical trial	(i) N = 13 edentulous patients with 78 implants [36 in smoker, and 42 in the nonsmokers] (ii) The mean of age was 53.5 and the range of age was 36-72 years (iii) 11 were males, and 2 were females	(i) To evaluate the effect of smoking habits on accuracy of implant placement using mucosally supported stereolithographic surgical guides.	(i) Mean coronal, angular deviation, and apical deviation were compared between smokers and nonsmokers, but they were not significant. However, there was a significant difference in global coronal and apical deviation between the smokers and the nonsmokers (P < 0.05). (ii) The mean value mucosal thickness was 3.19mm (range: 2.39–4.01mm) among the smokers compared with 2.43mm among the nonsmokers (range: 1.44-3.03mm) (P < 0.05). Smokers have significant thicker supporting mucosal tissues compared with nonsmokers, which may explain inaccuracy due to less stability of the surgical guide or the scanning prosthesis.

D'Haese et al., 2013 [119]	Prospective monocenter case-control study	(i) N = 26 cases for 114 implants using fluoride-modified OsseoSpeed: 17 nonsmokers, and 9 smokers (ii) The mean of age was 51.84 with a range of 20-81 (iii) 16 were males, and 10 were females	(i) To determine implant survival and success in terms of peri-implant bone loss and evaluate whether smoking affects the outcome of implants placed using stereolithographic guided surgery	(i) At patient level, implant loss was observed in 7 out of 26 patients (26.9%, 1 in nonsmokers versus 6 in smokers). (ii) The implant failure was 38.5% in subjects with a full immediately loaded fixed dental prosthesis and it was 15.4% in partially delayed loaded cases. (iii) At implants level, 13 out of 114 implants (1 in nonsmokers, and 12 in smokers) were lost within 12 months after surgery, resulting in 88.6% survival at 1 year. (iv) The overall mean bone loss based on all implants was 0.47 mm ± 0.94. Smokers had greater mean loss than nonsmokers (0.62 mm versus 0.36 mm).

Ferreira et al., 2016 [120]	Prospective controlled study	(i) N = 22: 11 never-smokers, 11 smokers (ii) The mean of age (Mean ± SD): 55.4 ± 4.5 years (iii) 13 were males, and 9 were females	(i) To evaluate the effect of cigarette smoking on the percentage of early bone-to-implant contact (BIC%) and the bone density in the threaded area (BA%) as well as the bone density outside the threaded area (BD%) around microimplants with sandblasted acid-etched surface retrieved from human jaws	(i) Early stages of maturation (new bone) were present primarily in never-smokers. However, two microimplants placed in smokers showed no osseointegration. Also, marginal bone loss, gap, and fibrous tissue were seen around some implants in smokers. (ii) According to histometric evaluation, compared to nonsmokers, smokers had lower BIC% (25.9 ± 9.1 and versus 39.8 ± 14.2, P = 0.02), BA% (28.6 ± 10.1 versus 46.4 ±18.8, P = 0.04), and BD% (19.1 ± 7.6 versus 28.5 ± 18.8, P = 0.21)

Garcia-Delaney et al., 2015 [121]	Case-control study	(i) N = 54: 27 had peri-implantitis and 27 had healthy implant (ii) The mean of age was 50.6 with a range of 40-66 years (iii) 18 were males (9 peri-implantitis, 11 control), and 34 were females (18 peri-implantitis, 16 control)	(i) To evaluate the relationship between IL-1 gene polymorphisms and peri-implantitis in smoking patients	(i) Patients with previous history of periodontitis had significantly higher incidence of peri-implantitis (OR=10.9, P = 0.024). (ii) Both groups were similar regarding IL-1A-C889T, IL-1B+ C3953T and IL-1RN+ T2018C genotypes. (iii) IL-1 polymorphism was not associated with increased risk of peri-implantitis in heavy smokers

Javed et al., 2016 [122]	Randomized prospective controlled study	(i) N = 166: 84 smokers (41 in test group, 43 control), and 82 nonsmokers (40 in test group, and 42 in control) (ii) Range of age was 28-55 years (iii) 120 were males, and 46 were females	(i) To assess the efficacy of mechanical debridement with and without adjunct antimicrobial photodynamic therapy in reducing dental peri-implant inflammation among cigarette smokers and nonsmokers	(i) At 6 months of follow-up, among smokers and nonsmokers, peri-implant PD was significantly higher in the control group compared with the test group (P < 0.05). (ii) At 12 months of follow-up, BOP, PD, and crestal bone loss CBL were not significantly differed between smokers and nonsmokers in the test and control groups.

Levin et al., 2008 [123]	Consecutive prospective cohort study	(i) N = 64: 49 nonsmokers, 5 quit smoking before 5 years, and 6 current smokers (ii) Average of age was 45 with a range of age 18-78 years (iii) 24 were males, and 40 were females.	(i) To compare the long-term marginal implant bone loss, survival, and radiographic success of single dental implants among current, former, and nonsmokers.	(i) Success rate was 93.75%. There were 4 implants failed: 2 due to mechanical neck broke and 2 resulted from peri-implantitis and bone loss. (ii) The survival rates were not correlated with smoking habits. (iii) During all time intervals, current and former smokers demonstrated higher marginal bone loss than nonsmokers, and current smokers demonstrated higher marginal bone loss than the former smokers.

Lin et al., 2012 [124]	Retrospective cohort study	(i) N = 75 subjects with 155 implants: 47 nonsmokers, 28 smokers (ii) 32 were males, and 43 were females	(i) To determine the effect of cigarette smoking and residual native bone height on the survival of dental implants placed immediately in grafted sinuses	(i) At stage-two surgery, the survival rates of implants for nonsmokers and smokers were 93% and 84%, respectively. (ii) After 12 months of functional loading, the survival rate of implants for nonsmokers was 87% and for smokers was 79% (P < 0.001). (iii) When the preoperative bone height is less than 4 mm, there was a significant effect of smoking on implant survival, with an 82.4% implant survival rate in nonsmokers compared to 60% in smokers (P < 0.05). (iv) Smoking considered high risk factor when implants are placed immediately in grafted sinuses in area with limited bone height

Malo et al., 2016 [125]	Retrospective cohort study	(i) N = 721 had systemic disorders or smoking (ii) Average of age was 51 years (iii) 299 males, and 422 females	(i) To evaluate long-term (3-17 years) outcome of dental implant rehabilitation in patients with systemic disorders and smoking habits	(i) 87 patients were lost to follow-up (12%). 45 patients had prosthetic failure which resulted in survival rate of 94.3%. (ii) 127 implants failed in 98 patients resulting in cumulative survival rate of 83.5% at patient and 94.6% at implant level. (iii) The average marginal bone levels were 1.18 mm at 1 years, 1.56 mm at 5 years, and 1.47 mm at 10 years. (iv) 86 patients (11.9%) had biological complications.

Negri et al., 2016 [126]	Cross-sectional clinical study	(i) N = 48 subjects: 23 nonsmokers, and 25 smokers (ii) The average of age: 53.23 ± 12.21 nonsmokers, and 51.72 ± 10.51 smokers (iii) 18 were males (7 nonsmokers, 11 smokers), and 30 were females (16 nonsmokers, 14 smokers) (iv) The majority were Caucasian (76% nonsmokers, 84% smokers)	(i) To evaluate the influence of chronic cigarette smoking on the profile of osteoimmunoinflammatory markers in the peri-implant crevicular fluid (PICF) from clinically healthy implants	(i) Compared to nonsmokers, smokers had significantly lower level of IL-4, 1L-8, and TNF-*α* (P < 0.05). However, OPG and RANKL/OPG were lower in smokers than in nonsmokers but not significant (P > 0.05). (ii) Higher cross-linked telopeptide of type I collagen (ICTP) concentrations and a higher TH1/TH2 ratio were observed in the PICF of the smokers compared with nonsmokers (P < 0.05)

Quaranta et al., 2015 [127]	Retrospective study	(i) N = 15 patients with 30 implants (ii) Average of age was 53.3 years (iii) 9 males, and 6 females	(i) To evaluate the impact of smoking and previous periodontal disease on peri-implant microbiota and health in medium to long-term maintained patients.	(i) There were no differences in bacterial counts between dental and implants sites in all groups. (ii) Nonsmokers with previous history of periodontal disease had significant high counts of pathogenic bacteria and an occasional positive BIOP values. (iii) Patients who were smokers with previous periodontal disease had clinical signs of inflammation (deep pockets and slight bone resorption) and significant counts of pathogenic bacteria.

Raes et al., 2015 [128]	Prospective cohort study	(i) N = 85 subjects: 39 nonsmokers, 46 smokers (ii) The mean of age was 42 for nonsmokers and 46 for smokers (iii) 42 were males (18 nonsmokers, 24 smokers), and 43 were females (21 nonsmoker, 22 smokers)	(i) To compare smokers to nonsmokers in terms of soft tissue alterations following single dental implant treatment in healed bone	(i) All implants were successfully integrated in nonsmokers, whereas 3 implants were failed in smokers. (ii) Papillae regrowth was significantly observed in nonsmokers, whereas smokers showed stable papillae (P = 0.025). (iii) Midfacial soft tissue level demonstrated significant regrowth in nonsmokers, whereas it remained stable in smokers (P = 0.004).

Rodriguez-Argueta et al., 2011[129]	Retrospective cohort study	(i) N = 295 patients, 1,033 dental implants: 182 nonsmokers, and 113 smokers (ii) Average of age was 53.1 ± 12.5 with a range of 21-68 years (iii) 127 were males, and 168 were females	(i) To identify the risk of complications in a group of patients treated with osseointegrated implants and to assess the effect of smoking on these complications	(i) 209 complications were reported: 32 cases of implant loss, 2 cases of infection, 70 cases of peri-implantitis, and 105 cases of mucositis. (ii) There was an association between smoking and increased risk of complications (P = .008)

Romanos et al., 2013 [130]	Prospective study	(i) N = 20 patients: 12 nonsmokers (97 implants: 55 in maxilla, and 42 in mandible), 8 smokers (66 implants: 36 in maxilla, and 30 in mandible) (ii) The average of age: 65.91 ± 4.86 for nonsmokers, and 55.71 ± 7.83 for smokers (iii) 12 were males (8 nonsmokers, 4 smokers), and 8 were females (4 nonsmokers, 4 smokers)	(i) To evaluate the long-term success of immediately loaded platform-switched implants placed in smokers and nonsmokers with edentulous jaws.	(i) Three implants failed during the loading period of 62.53 ± 44.13 months for the smokers and 98.20 ± 19.53 months for the nonsmokers. (ii) Two failed in smokers: 1 due to group overloading and 1 because of peri-implanitis, while the 1 that failed in nonsmoker was related to overloading. (iii) Plaque, bleeding, probing pocket depths, or crestal bone loss (medially or distally) was not significantly differed between smokers and nonsmokers. (iv) The success rate was 92.42% for smokers and 98% for nonsmokers.

Romanos & Nentwig, 2008 [131]	Consecutive case reports	(i) N = 9 patients received 72 implants (all were heavy smokers, smoked > 2 packs/day for 10 years) (ii) The average of age (Mean ± SD): 52.4 ± 8.3 years (iii) 5 were males, and 4 were females	(i) To evaluate the long-term success and the peri-implant soft and hard tissue conditions around immediately occlusal loaded implants in edentulous jaws of heavy smokers	(i) There was 1 mobile implant after 6-66 months of loading period (mean = 33.7 ± 19.0). (ii) All clinical indices were in normal ranges and the Periotest values were decreased with time which indicated that implant becomes more secured in bone. (iii) The long-term success was 98.6% for immediately loaded implants placed in occlusal function in smokers restored with fixed cross-arch implant-supported restorations.

Sayardoust et al., 2013 [132]	Retrospective clinical radiographic case-controlled study	(i) N = 80 patients with advance periodontitis: 40 never-smokers (118 implants), 40 smokers including (134 implants) (ii) The mean of age was approximately 61.5 for never-smokers, and 54 for smokers (iii) 38 were males, and 42 were females	(i) To determine implant survival and marginal bone loss at turned and oxidized implants in smokers and never-smokers with periodontitis	(i) Smokers had lower survival rate than never-smokers (89.6% versus 96.9%). (ii) Compared with oxidized implants, turned implants failed more frequently in smokers. (iii) Smokers had higher likelihood ratio for implant failure than that of never-smoker (4.68 and after subgrouping analysis it was 6.40 for turned and 0.00 for oxidized implants). (a) In smokers, the mean of marginal bone loss at 5 years was 1.54 ± 0.21 mm at turned and 1.16 ± 0.24 mm at oxidized implants. In contrast, in never-smokers, significantly greater bone loss was found at oxidized implants than at turned implants (1.26 ± 0.15 mm versus 0.84 ± 0.14mm). (b) Oxidized implants demonstrated similar bone loss for both groups. Turned implants lost significantly more bone in smokers

Shibli et al., 2010 [133]	Prospective controlled study	(i) N = 24 subjects: 11 nonsmokers, and 13 smokers (ii) The average of age (Mean ± SD): 51.32 ± 7.5 (iii) 10 were males, and 14 were females	(i) To evaluate the impact of smoking on bone-to-implant contact, the bone density in the threaded area, and the bone density outside the threaded area around microimplants with oxidized surface retrieved from human jaws.	(i) Three microimplants placed in smokers showed no osseointegration. Marginal bone loss, gap, and fibrous tissue were present around implants retrieved from smokers. However, the newly formed bone showed early stages of maturation, mainly in the nonsmokers. (ii) According to histometric evaluation, compared to nonsmokers, smokers had lower bone-to-implant contact (25.97 ± 9.02 and versus 40.01 ± 12.98, P < 0.001), bone density (28.17 ± 10.32 versus 46.34 ± 19.12), and bone density outside the threaded area (18.76 versus 25.11, P > 0.05).

Stoker et al., 2012 [134]	Randomized- controlled clinical trial	(i) N = 104 completed study out of 110 edentulous patients treated with overdenture that supported by:37 [2 implants with ball attachments (2IBA)], 37 [2 implants with a bar (2ISB)], and 37 [4 implants with a triple bar (4ITB)] (ii) Average of age at time of evaluation was 59.8 years (iii) 22 were males, and 66 were females	(i) To compare the differences in the long-term clinical and radiologic effect for three different treatment strategies with implant-supported overdentures in the edentulous mandible, with a special emphasis on smoking	(i) In 2IBA group, plaque index was significantly lower than in the other (versus 2ISB, P = 0.013; versus 4ITB, P = 0.001) groups, but it was not associated with other peri-implant parameters. (ii) The marginal bone loss was significantly higher in the 4ITB group than that in the two implant groups. (iii) The maximal probing depth was correlated with peri-implant bone loss (P = 0.011). (iv) Smoking found to double the marginal bone loss regardless of the treatment strategy.

Sun et al., 2016 [135]	Prospective clinical study	(i) N = 32 subjects with 45 ITI Straumann dental implants (16 nonsmokers, 16 heavy smokers) (ii) The range of age was 25 -65 years (iii) 22 were males, and 66 were females	(i) To evaluate the implant stability and peri-implant tissue response in heavy smokers receiving dental implants due to partially edentulous posterior mandibles	(i) In both groups of smokers and nonsmokers, the implant stability quotient (ISQ) decreased from the initial ISQ and then increased starting from 2 weeks after surgery. However, at 3, 4, 6, and 8 weeks after surgery, the ISQ differed significantly between nonsmokers and heavy smokers. (ii) At 12th week after surgery, all implants achieved osseointegration without complications, and at 1 year success rate was 100% for smokers and nonsmokers. (iii) At 6 or 12 months after loading, the marginal bone loss and probing depth were significantly higher in heavy smokers than in nonsmokers, whereas the modified plaque index and modified sulcus bleeding index did not differ significantly between both groups of smokers and nonsmokers

Sverzut et al., 2008 [136]	Retrospective cohort study	(i) N = 650 patients with 1,628 implants in maxilla and mandible: 574 nonsmokers, 76 smokers (ii) The average of age was 42.7 with a range of 13-84 years	(i) To assess tobacco use as a risk factor for early maxilla and mandible implants failure	(i) The early implant loss rate was 3.32% in smoking group and 2.81% in nonsmoking group. (ii) There was no significant association between early implant losses and frequency of tobacco use

Tatli et al., 2013 [137]	Cross-Sectional Study	(i) N = 60 patients with 60 dental implants: 33 nonsmokers, 27 current smokers (ii) The average of age was 44.9 ± 10.46 with a range of 29-62 years (iii) 30 were males (18 nonsmokers, 12 smokers), and 30 were females (15 nonsmokers, 15 smokers)	(i) To evaluate the effects of smoking on peri-implant health status and inflammatory cytokines IL-1, TNF-*α*, and prostaglandin E2 levels in PICF	(i) All clinical parameters with the exception of plaque scores were significantly higher in the smoker compared to nonsmoker. (ii) Cytokines were significantly increased in smoker group than in nonsmoker group. The correlations between the cytokine levels and clinical parameters were more marked in smokers.

Thalji et al., 2015 [138]	Prospective cohort study	(i) N = 21 subjects with 84 mini-implants: 11 nonsmokers, 10 smokers (ii) The average (range) of age was 60.2 (47-69), 50.8 (33-63) years in nonsmokers, smokers, respectively.	(i) To evaluate the impact of smoking on the early molecular events involved in peri-implant healing at either a microroughened or a microroughened with superimposed nanofeatures surface implant.	(i) After 7 days of follow-up, the study failed to identify differences in the gene expression profiles (whole genome array) of implant adherent cells at this early stage of osseointegration comparing smoker and nonsmoker individuals.

Twito & Sade, 2014 [139]	Retrospective consecutive cohort study	(i) N = 7,680 implants (ii) The average of age (Mean ± SD): 41.48 ± 5.61 with a range of 22 -55 years (iii) 6,731(87.6%) implant were placed in men and 949 (12.4%) in women	(i) To investigate the impact of smoking habits and other relevant factors on dental implant survival	(i) The survival rate was 95.8%; 321 out of 7,680 implants did not survive (4.2%). (ii) Smoking status (smoking/no smoking) and amounts of smoking as expressed in pack-years were inversely associated with implant survival rate

Vacharaksa et al., 2015 [140]	Cross-sectional study	(i) N = 52 submucosal samples obtained from dental implants (37 bone level, 15 tissue level) (ii) The average of age (Mean ± SD): 51.92 ± 11.54 for bone level, and 52.7 ± 5.69 for tissue level (iii) 24 were males (18 bone level, 6 tissue level), and 23 were females (19 bone level, 4 tissue level)	(i) To compare peri-implant microbiota associated with implant transmucosal designs or smoking habits	(i) Bone level implant had at least 1 more pathogen than that of tissue level; however, differences in each bacterium were not significant. (ii) Treponema denticola had significantly more prevalence and abundance in smokers than in nonsmokers (P < 0.05). (iii) Smokers and nonsmokers exhibited similar peri-implant microbiota based on the PhyloChip Array analysis for 5 smokers and 5 nonsmokers.

Vandeweghe & De Bruyn, 2011 [141]	Retrospective cohort study	(i) N = 329 patients with 712 installed implants ( 608 in 288 nonsmokers, 104 in 41 smokers) (ii) The average of age (Mean ± SD): 54 ± 13.44 with a range of 18-84 (iii) 141 were males, and 188 were females	(i) To determine the effect of smoking on early implant failures and bone remodeling around moderately rough implants in human jaws	(i) The overall survival rate was 98.3%. Failure rate was threefold higher in smokers than in nonsmokers (4.8% versus 1.2%); this findings was statistically significant on implant rather than patient level. (ii) Sixty implants from 21 smokers lost significantly more bone than the 303 implants in 148 nonsmokers (P = 0.001). (iii) The maxilla is especially prone to bone loss compared with the mandible (1.70 mm versus 1.26 mm, P < 0.001)

Vervaeke et al., 2012 [142]	2-years retrospective cohort study	(i) N = 300 patients (849 nonsmokers, 235 smokers), with 1,106 dental implants (ii) The average of age (Mean ± SD): 56 ± 12.05 with a range of 17-82 (iii) 114 were males, and 186 were females	(i) To compare the survival and peri-implant bone loss of implants with a fluoride-modified surface in smokers and nonsmokers	(i) 19 implants in 17 patients failed; the overall survival rate at implant level was 98.3% and 94.6% at the patient level. (ii) After a follow-up period of 2 years, the cumulative survival rate was 96.7% at patient and 99.1% at implant. (iii) Compared with smokers, implant survival was significantly higher for nonsmokers (implant level P = 0.025; patient level P = 0.017). (iv) The overall mean bone loss was 0.34 mm ± 0.65. Smokers lost significantly more bone compared with nonsmokers in the maxilla (0.74 versus 0.33 mm; P < 0.001), but not in the mandible (0.25 versus 0.22; P = 0.298)

***1.F:Tobacco Smoking and Bone Graft (5 Articles)***				

Costa et al., 2016 [143]	RCT (12 months)	(i) 19 smokers with 38 Miller Class I and II: 19 received both acellular dermal matrix graft (ADMG) and enamel matrix derivative (EMD and 19 control (ADMG alone) (ii) The range of age was 30-50	(i) Study investigated whether EMD contributes to root coverage of gingival recessions when performed with or without ADMG among the smokers	(i) Compared with the control, the intervention group has higher percentage of root coverage (59.7% versus 52.8%) and statistical significance in mean gain in recession height and sites with complete coverage.

Lindfors et al., 2010 [144]	Prospective cohort study	(i) N = 26 patients with 27 bone augmentation using bone graft titanium-reinforced ePTFE membranes: 17 nonsmokers, 8 smokers (ii) The range of age was 17-68 years (iii) 8 were males, and 18 were females	(i) To evaluate treatment outcomes achieved using guided bone regeneration (GBR) with autogenous bone grafting (alveolar crest), particularly the effect of smoking and membrane exposure	(i) Twenty-three (85%) augmentations were successful and 4 (15%) were unsuccessful. Augmentation was successful in 95% of nonsmokers and in 63% in smokers. (ii) Signs of soft tissue inflammation were present in 10 (37%) of the augmentation sites, more often in smokers (75%) than in nonsmokers (21%) (P = 0.008)

Nystrom et al., 2009a [145]	Prospective, long-term (9-14 years), follow-up study	(i) N = 334 implants in 44 patients: 89 implants in smokers, and 245 implants in nonsmokers (ii) 44 patients: 12 active smokers, 5 stopped smoking before surgery, and 27 nonsmokers (iii) Average age in years [(Mean, (range)]: 58 (45-68) (iv) 111 implants in 15 Males, and 223 implants in 29 females	(i) To investigate treatment outcome of onlay bone grafting in the atrophic maxilla and the impact of gender and smoking in long-term study concerning implant survival rate and marginal bone loss adjacent to the surfaces of the implant	(i) 27 out of 334 inserted Branemark implants, with machined surface, were failed. (ii) The implant survival rate was found to be significantly different between genders (P = 0.017). (iii) Marginal bone loss was 1.8 mm 1 year after implant surgery; 2.3 mm after 5 years; and 2.4 mm after 10 years. (iv) The significant difference in marginal bone loss was observed between genders up to 4 years of examination, and such difference was observed up to 5 years of examination between group smokers and nonsmokers

Nystrom et al., 2009b [146]	Prospective, long-term (11-16 years), follow-up study	(i) N = 167 implants in 26 patients: 36 implants in smokers, and 131implants in nonsmokers (ii) 26 patients: 6 active smokers, 4 stopped smoking before surgery, and 16 nonsmokers (iii) Average age in years [(Mean, (range)]: 54.7 (38-70) (iv) 82 implants in 13 Males, and 85 implants in 13 females	(i) To investigate treatment outcome of A Le Fort I osteotomy and interpositional bone graft in combination with implants in the atrophic maxilla and impact of gender and smoking on implant survival rate and marginal bone loss adjacent to the surfaces of the implant	(i) 24 out of 167 implants were failed with a survival rate of 855 at the end of the follow-up. (ii) The implant survival rate was found to be not significantly different based on gender or smoking status. (iii) Marginal bone loss was 2.5, 2.9, 3.0, and 3.1mm from the implant-abutment junction, after 1, 2, 5, and 10 years of follow-up, respectively. (iv) The bone level stabilized after 2 years.

Yilmaz et al., 2010 [147]	Controlled clinical trial with a parallel design	(i) 24 advanced chronic periodontitis with 113 intrabony defects: 12 smokers (smoked regularly 10 cigarettes on a daily basis), and 12 nonsmokers (ii) The age ranged from 32 to 50 years	(i) To assess the healing response of intrabony defects after a combined regenerative treatment [bovine-derived xenograft (BDX) and platelet-rich plasma (PRP)] in two groups of smokers and nonsmokers	(i) The probing depth reduction (P < 0.05), attachment (P < 0.001), clinical (P < 0.001), and radiographic bone gains (P < 0.001) were found to be significantly different between smokers and nonsmokers

**Section 2: Joint ( 54 Articles)**				

***2.A: Tobacco Smoking and Rheumatoid Arthritis(RA) (29 Articles)***				

Abhishek et al., 2010 [148]	Retrospective case-control study	(i) N = 395: 42 poor response (nonsmokers = 10, Ex-smokers = 16, current smokers = 13), and 353 with ≥ moderate response (nonsmokers = 123, Ex-smokers = 157, current smokers = 58) (ii) Average age in years (Mean ± SD): 61.86 ± 13.58 poor response, 60.67 ±11.77 for ≥ moderate response (iii) 89 were males (6 in poor response, and 83 in ≥ moderate response), and 306 were females (36 in poor response, and 270 in ≥ moderate response)	(i) To assess if smoking status among RA patients at the time of commencing anti-TNF-*α* reduces the odds of achieving at least moderate response on the criteria of European League A Rheumatism (EULAR) at 3-month assessment	(i) Forty-two patients failed to show at least moderate response on the EULAR criteria. (ii) Compared with nonsmoking, current smoking at time of commencing all anti-TNF-*α* agent reduced the likelihood in achieving at least a moderate response on the EULAR (Adjusted OR = 0.20, 95% CI = 0.05-0.83, P = 0.03).

Afridi et al., 2011 [149]	Cross-sectional study	(i) N = 105: 53 RA patients [23 nonsmokers, 30 smokers], 52 referents (controls) [26 nonsmokers, 26 smokers] (ii) The age ranged of 42-56 for all group and matched in age and sex	(i) To compare the level of essential and trace elements in scalp hair of smoker and nonsmokers with RA and matched healthy controls of smokers and nonsmokers	(i) Compared to health individual, the mean contents of hair Zn, Cu, and Mn were significantly lower in smokers and nonsmokers RA patients (P = 0.01-0.001). However, Cd and Pb were significantly higher in scalp hair samples of RA patients (smokers and nonsmokers) (P < 0.001). (ii) Compared to nontobacco smokers, the referent smokers had significantly higher scalp hair levels of Cd and Pb (P < 0.01)

Afridi et al., 2015 [150]	Cross-sectional study	(i) N = 105: 53 RA patients [23 nonsmokers, 30 smokers], 52 referents (controls) [26 nonsmokers, 26 smokers] (ii) The age ranged of 42-56 for all group and matched in age and sex	(i) To examine the concentrations of trace essentials (Cu, Mn, Zn, and Se and toxic elements As, Pb, Cd, and Hg in the hair and blood samples of smoker and nonsmoker RA patients and to compare it with age- and sex-matched health control)	(i) Compared to healthy controls, the mean values of four toxic elements were significantly higher in scalp hair and blood samples of RA patients, while the levels of trace essential elements were found to be lower in RA patients, and the difference was significant in the case of smoker patients (P < 0.001)

Chen et al., 2012 [151]	Prospective cohort study	(i) N = 419: 141 nonsmokers, 278 ever-smokers (74 current, and 204 former) (ii) Mean of age was 62 years and with a range of 54-69 (iii) 137 were males, and 282 were females (iv) All were Caucasian	(i) To assess whether common genetic variants of the vascular endothelial growth factor A (VEGFA) gene had influence on serum VEGF-A levels and disease activity in RA	(i) VEGFA-2578 AA genotype was associated with lower serum VEGF-A, as was the most frequent haplotype (A_2578-C_460-G+405, 48.1%) within the 5'-flanking region of the gene. (ii) In never-smokers, the VEGFA-2578 AA genotype and (A_2578-C_460-G+405) haplotype were associated with decreased RA disease activity. (iii) In never-smokers, the VEGFA-2578 SNP was associated with disease activity at presentation (P = 0.029) and over time (P = 0.016) independent of serum VEGF-A levels.

Finckh et al., 2007 [152]	Prospective observation study of a community-based cohort	(i) N = 2004: 1459 nonsmokers, 544 smokers [489 moderate smokers (≤ I pack/day), 55 heavy smokers (> I pack/day) ] (ii) Average age in years (Mean ± SD): 56 ± 13 nonsmokers, 52 ± 13 moderate smokers, and 51 ± 10 in heavy smokers (iii) 94 were males, and 503 were females	(i) To compare the rates of radiographic progression among the smokers and nonsmokers in a large RA cohort	(i) The rates of progression for radiographic joint damage were found to be not significantly differed between status of current smoking and nonsmoking (P = 0.26). However, heavy smokers (>1 pack-day) were significantly associated with the radiographic progression than the progression seen in nonsmokers and moderate smokers (P < 0.001)

Fisher et al., 2014 [153]	Case-control study	(i) N = 1,614: 513 cases of RA ( 96 ever-smokers), and 1101 controls (139 ever-smokers) (ii) Average age in years: 51.0 ± 12.5 for RA cases, and 36.8 ± 12.3 for control (iii) 213 were males (72 in RA cases, and 141 in control) 1,401 were females (441 in RA cases, and 960 in control)	(i) To investigate gene-environment interaction between smoking and HLA-DRB1 shared epitope (SE) with antibodies to citrullinated peptides/proteins (ACPA) [citrullinated *α*-enolase (CEP-1) and vimentin (cVim) peptides] in a Korea cohort	(i) There was a particular association of smoking with levels of anti-CEP-1. However, a gene-environment interaction was associated with all the ACPA positive subgroups, although the highest odd ratio was seen with the subgroup of anti-CCP+/cVim+. (ii) When SE was absent, only smoking provided risk for subset with anti-CCP negative subgroups. (iii) There was no association between existence of erosions and the number of positive ACPA

Inoue et al., 2015 [154]	Cross-sectional study	(i) N = 5,216:3,335 never-smokers [162 male, 3,173 females], 1,159 former smokers [440 males, and 719 females], 523 current smokers [208 males, and 314 females] (ii) The range for the average age for the three smoking groups: 61.6-65.6 males, 56.7-62.3 females (iii) 810 were males, and 4,206 were females	(i) To analyze sex difference in the effect of smoking on remission proportions in patients with rheumatoid arthritis (RA)	(i) In males, compared with former and current smokers, never-smokers exhibited higher remission rate; however in women smoking status had no effect on remission. (ii) In males, compared to never-smokers, past and current smokers were negatively associated with DAS28-erythrocyte sedimentation rate remission [OR = 0.66, 95% CI: 0.44-0.98 for past, and OR = 0.61, 95%CI: 0.39-0.96 for current smokers]. However, in females, past and current smokers were not associated with remission compared to female never-smokers.

Kim et al., 2015 [155]	Secondary analysis for data compiled from Case-control and prospective cohort study	(i) N = 6,302 from 3 case-control studies (ii) The range for the average age in the 3 case-control studies: 34.67-54.33 (iii) The majority were females among the three case-control studies with percentage ranged within 70.44 -100%	(i) To describe the interaction between cigarette smoking and HLA polymorphisms in seropositive RA (based on the recently identified amino acid-based HLA model for RA susceptibility) patients from 3 case-control studies (EIRA study, NHS, and Korean RA Cohort Study)	(i) The susceptibility for RA was associated with heavy smoking, and all investigated HLA amino acid positions and its related haplotypes (ii) Based on the interaction analysis, it was found there was a significant joint effect between heavy smoking and the HLA-DR beta 1 4-amino acid haplotype primarily by positions 11 and 13 [Attribute proportion to interaction (AP) of 0.416, 0.467, and 0.796, in the EIRA, NHS, and Korean studies, respectively]

Kristiansen et al., 2014 [156]	Retrospective cohort study	(i) N = 978: 445 patients with RA (311 ever-smokers), and 533 sex- and age-matched controls. (ii) (323 ever-smokers) (iii) The mean of age [mean(range)]: 52 (19-69) for RA, and 53 (19-69) for controls (iv) 519 were males (313 RA, and 20c control), and 459 were females (132 RA, and 327 control)	(i) To investigate whether polymorphisms in genes coding for [mannose-binding lectin (MBL) and surfactant protein-D (SP-D)] are associated directly or by interaction with smoking with RA, ACPA positive RA, and erosive RA	(i) The low-producing Surfactant, pulmonary-associated protein-D (SFTPD) genotype was associated with erosive disease in the RA patients (OR = 1.8; 95% CI: 1.1-3.0) predominantly in RA ever-smokers (OR = 2.4; 95% CI: 1.3-4.3), however; it was not associated with risk of RA or ACPA positive RA. (ii) The high-producing MBL2 genotype YA/YA was associated with erosive joint disease in RA ever-smokers (OR = 1.8; 95 % CI 1.1-3.0) and with ACPA positive RA (OR = 1.4; 95% CI: 1.0-1.9). (iii) Genetic disposition for low SP-D was not associated with RA but with erosive RA by interaction with smoking. (iv) The genetic disposition for high MBL production was associated with ACPA positive RA regardless of shared epitope

Krol et al., 2015 [157]	Cross-sectional study	(i) N = 153: 49 nonsmokers, 104 ever-smokers (45 current, and 59 former) (ii) Mean of age was 53 with a range of 20-75 years (iii) 53 were males, and 100 were females	(i) To examine to what degree the smoking, shared epitopes, and anti-cyclic citrullinated peptide (anti-CCP) antibodies are associated with erosive disease and disease activity in patients RA at disease onset	(i) The prevalence of patients with shared epitopes (SE) was 48% for 0, 49% for 1, and 23% for 2 SE. (ii) Anti-CCP antibodies, IgM-RF, and IgA-RF were present in 58%, 65%, and 54% patients, respectively. (iii) In smokers, erosive disease was associated with anti-CCP antibodies (OR = 3.9, 95% CI: 1.6-9.3), IgM-RF (OR = 4.9, 95%CI: 1.9-12), and IgA-RF (OR = 2.8, 95%CI: 1.2-6.4), but it was absent with regard to SE. (iv) In never-smokers, there was no association between the erosive disease and any shared epitopes or antibodies. (v) There was an association between all measured levels antibody and smoking and shared epitopes.

Lu et al., 2014 [158]	Prospective cohort study	(i) N = 662: 353 never, 250 former, and 56 current smokers (ii) Average of age (Mean ± SD): 54.6 ± 14.3 never, 61.2 ± 11.0 former, and 54.5 ± 11.453 for current smokers were males, and 100 were females (iii) The majority were females (83.0% of never, 82.4% of former, and 78.6% of current smokers) (iv) The majority were white (93.1% of never, 97.2% of former, and 89.1% of current smokers)	(i) To investigate the associations of smoking and alcohol consumption with disease activity and functional status in RA	(i) Current smoking was not associated with DAS28-CRP3 but was associated with a higher Modified Health Assessment Questionnaire (MHAQ) than nonsmokers with seropositive RA (P = 0.05). (ii) Compared to no alcohol use, Consumption of 5.1-10.0 g/day of alcohol was associated with a significant decrease of MHAQ (P = 0.02). Such effect for alcohol consumption was observed to be stronger in RA patients with HLA-SE positive compared to HLA-SE negative. (iii) When stratified by HLA-SE status, past smoking was associated with DAS28-CRP3 only in HLA-SE–positive patients, but not in HLA-SE–negative patients (P for interaction = 0.02). Although there was no significant interaction between HLA-SE and smoking for MHAQ (P > 0.05), the effect of smoking tended to be stronger in HLA-SE–positive patients compared to HLA-SE–negative patients

Maska et al., 2012 [159]	Randomized, blinded, placebo-controlled clinical trial	(i) N = 412: 293 nonsmokers, 119 current smokers (ii) The average of age (Mean ± SD) was 49.6 ± 12.2 (iii) 270 were males, and 142 were females	(i) To investigate the association between serum cotinine and treatment efficacy in early rheumatoid arthritis	(i) There were no differences in the mean of DAS28 between 48 and 102 weeks based on smoking status (P = 0.881) or treatment (early combination or initial MTX with step-up therapy at 24 weeks)

Mattey et al., 2009 [160]	Prospective cohort study	(i) N = 154: 51 never-smokers, 103 ever-smokers (65 former, 38 current) (ii) The average of age (Mean ± SD): 54.5 ± 12.3 never-smokers, 57.7 ± 12.9 former, and 56.4 ± 12.60 for current smokers (iii) 44 were males, and 110 were females	(i) To determine if there is a quantitative relationship between smoking history and response to therapy with tumor necrosis factor (TNF) antagonists I patients with RA	(i) When smoking pack-year (py) was increased, there was a significant increase in trend of no response at 3 and 12 months [P (trend) = 0.008 and 0.003, respectively]. (ii) There was an inverse association between the change in DAS28 over the first 3 months and number of py (r = -0.28, P = 0.002).

Naranjo et al., 2010 [161]	Cross-sectional data from the multinational QUEST-RA database	(i) N = 7,307: 4,749 never-smokers, 2,558 ever-smokers (1,091 former, and 1,467 current) (ii) The average of age was 55.17 (iii) 1,468 were males, and 5,773 were females	(i) To analyze clinical severity/activity of RA based on the smoking status.	(i) Ever-smokers were more likely to be RF-positive (OR = 1.32; 95%CI: 1.17-1.48, P < 0.001) and to have more frequent rheumatoid nodules (OR = 1.41; 95%CI: 1.24-1.59, P < 0.001). (ii) There were no differences between all smoking categories in regard to the percentage of patients with erosive arthritis and extra-articular disease. (iii) Compared to nonsmokers, ever-smoker had lower mean of DAS28 (4.0 ±1.6 versus 4.4 ± 1.6, P > 0.05).

Nyhall-Wahlin et al., 2009 [162]	Case-control study nested in a cohort study [BARFOT (Better AntiRheumatic FarmacOTherapy)]	(i) N = 160 RA: 40 cases of extra-articular RA (ExRA) [7 never, 15 former, and 18 current smokers], and 120 age-sex-duration symptoms matched controls RA controls [32 never, 50 former, and 32 current smokers] (ii) The average of age (Mean±SD): 58.5 ± 11.6 for ExRA, and 58.5 ± 11.1 for control (iii) 44 were males, and 116 were females	(i) To identify patients with severe extra-articular RA (ExRA) in an early RA cohort and to investigate potential risk factors	(i) Positive RF was more prevalent in ExRA cases than the controls (93% versus 59%). (ii) Patients who developed severe ExRA were more often current smokers and had higher mean of DAS28, HAQ, and CRP at baseline. (iii) During the first 2 years of follow-up, study found DAS28 (OR = 7.79/SD; 95%CI: 3.04-19.95), HAQ (OR = 2.30/SD; 95%CI: 1.37-3.88), and CRP (OR = 3.05/SD; 95% CI 1.77- 5.26) were acting as strong predictors of ExRA development

Rojas-Serrano et al., 2011 [163]	Prospective cohort study	(i) N = 144 of early RA participants received treated methotrexate and sulfasalazine: 22 current smokers (ii) The average of age (Mean±SD):37.4 ± 12.23 (ii) 17 were males, and 127 were females	(i) To determine factors associated with a non-ACR 50 response at 6 months of follow-up in patients with early RA	(i) 144 had a complete follow-up, and 62 (43%) did not reach an ACR 50 response at 6 months. Current smoking was the only factor associated with this outcome (OR = 3.58, P < 0.008, 95%CI: 1.23-11.22).

Roos et al., 2016 [164]	Prospective cohort study	(i) N = 636 RA patients: 493 nonsmokers, 143 ever-smokers (ii) The average of age (Mean±SD): 57.6 ± 15.0 (iii) 210 were males, and 426 were females	(i) To evaluate the possible occurrence and clinical correlations of circulating secretory immunoglobulin A (SIgA) antibodies against the second-generation cyclic citrullinated peptides (CCP) among patients with recent-onset RA.	(i) Seventeen percent (17%) of the patients were found to be positive for circulating SIgA anti-CCP. (ii) Positive baseline of SIgA anti-CCP was associated with slightly higher means of baseline ESR (38 versus 31 mm/first hour, P = 0.004) and CRP (30 versus 23 mg/L, P = 0.047). (iii) During follow-up, SIgA anti-CCP-positive patients had a higher mean AUC regarding ESR (adjusted P = 0.003), although there were no significant differences regarding CRP, tender and swollen joint counts, or radiological joint damage. (iv) SIgA anti-CCP was associated significantly with smoking (AOR 2.19, 95%CI: 1.01-4.37, P = 0.027) but not with carriage of the SE (P = 0.62).

Ruiz-Esquide et al., 2011[165]	Prospective cohort study	(i) N = 156: 90 never-smokers, 13 former smokers, and 47 current smokers (23 heavy smokers) (ii) The average of age (Mean±SD): 54.4 ± 14.9 (iii) 26 were males, and 130 were females	(i) To analyze the effects of cigarette smoking on disease activity and radiographic damage in patients with early RA.	(i) Baseline data shows that ever-smokers had earlier disease onset and a closer association with the SE, but not more seropositive disease. (ii) Smokers and nonsmokers were not significantly different in disease activity and EULAR therapeutic responses. (iii) The baseline of Larsen score in multivariate analysis showed the HLA-DRB*∗*04 genotype, being female, and current smoking were associated with radiographic progression.

Saevarsdottir et al., 2011 [166]	Retrospective cohort study [data retrieved clinical from Epidemiological Investigation of Rheumatoid Arthritis (EIRA) ]	(i) N = 1,430: 873 received methotrexate (MTX) at inclusion, and 535 later received tumor necrosis factor (TNF) inhibitor: 406 never, 402 former, 368 current, and 176 irregular smoking/other tobacco (ii) The median of age was 54 and the interquartile range was 44-61 (iii) 438 were males, and 992 were females	(i) To determine whether cigarette smoking influences the response to treatment (MTX and TNF Inhibitors) in patients with early RA	(i) Current smokers were less likely to achieve a good response at 3 months following the start of MTX (27% versus 36%; P = 0.05) and at 3 months following the start of TNF inhibitors than never-smokers (29% versus 43%; P = 0.03). (ii) There was an inverse association between current smoking and good response remained for MTX (AOR = 0.60, 95%CI: 0.39-0.940) and TNF inhibitor (AOR = 0.52, 95%CI: 0.29-0.96) and the lower likelihood of a good response remained at later follow-up visits. Such finding was similar for remission or joint counts. (iii) Past smoking had no effect on response to MTX or TNF inhibitors. (iv) Current smoking was associated with a lower odd of a good response (AORs for the 3-month, 6-month, 1-year, and 5-year visits 0.61, 0.65, 0.78, 0.66, and 0.61, respectively).

Salinas et al., 2015 [167]	Cross-sectional study [data from Argentine Consortium for Early Arthritis (CONAART)]	(i) N = 1,305 [729 with RA and 576 RA patients with undifferentiated arthritis(UA)]: 879 never-smokers, 124 former smokers, and 302 current smokers (ii) The average of age (Mean ± SD): 48.0 ± 14.0 years (iii) 235 were males, and 1,070 were females	(i) To analyze the effects of cigarette smoking on disease activity, functional capacity, radiographic damage, serology and presence of extraarticular manifestations in patients with RA and UA	(i) In patients with RA, disease activity score of 28 joints was significant differed between the three groups of current smokers, former smokers, and never-smokers (5.4 ± 1.3, 5.2 ± 1.4, 5.1 ± 1.4, respectively, P = 0.011). (ii) In patients with RA, simple erosion narrowing score was higher in current and former smokers than in never-smokers (P = 0.006), and rheumatoid factor titer was higher in current smokers compared with former and never-smokers (P = 0.004). (iii) Simple erosion score was independently associated with tobacco exposure among RA patients (OR = 1.03, 95%CI: 1.00-1.05; P = 0.012). (iv) In patients with UA, there were no associations between smoking status and parameters of activity or radiographic damage. (v) Tobacco exposure was not related to the presence of extra-articular manifestations or to the degree of disability in RA and UA patients. Also, no relationship was found between pack-years and disease activity and severity

Soderlin et al., 2013 [168]	Prospective cohort study [data from BARFOT]	(i) N = 1,421: smoking data available for 1,379 [514 smokers, 634 smokers, and 231 current smokers]	(i) To determine the prevalence of secondhand smoke exposure and to investigate the effect of secondhand exposure to tobacco smoke on disease activity of Swedish RA patients who had never smoke	(i) 68% patients (963 out of 1,421) had had secondhand exposure to tobacco smoke. (ii) The follow-ups for EULAR response at 3, 6, and 12 months and at 2 years for the patients who had never smoked and who had been exposed or had not been exposed secondhand to tobacco smoke were found to be not significantly different (P = 0.91, P = 0.88, P = 0.84, P = 0.61 and P = 0.85, respectively)

Soderlin and Bergman, 2011 [169]	Prospective cohort study [sample derived from BARFOT]	(i) N = 1,587: at inclusion time 508 former smokers, 381 current smokers (ii) The average of age: 58.0 ± 15.0 years (iii) 507 were males, and 1,080 were females	(i) To study the effect of disease duration and smoking on outcome in early RA receiving DMARD (predominantly methotrexate and sulfasalazine) and glucocorticoids	(i) The percentage of patients who had disease duration ≤12 weeks was 11% (180 RA patients). These patients attained good EULAR response significantly at 3 and 12 months than patients with a longer disease duration despite having more aggressive disease. However, Smokers had poorer EULAR response and showed no improvement with regard to disease duration. (ii) There was a significant association between improvement in DAS28 and HAQ and disease duration up to 12 months, but such trend was not seen among the smokers.

Soderlin et al., 2011 [170]	Prospective cohort study [sample derived from BARFOT]	(i) N = 1,787: at inclusion time 791 never, 575 former, and 421 current smokers (ii) Average of age (Mean ± SD): 58.0 ± 15.0 years (iii) 571 were males, and 1,216 were females	(i) To assess the effects of smoking on disease outcome in a large cohort of patients with early RA receiving DMARD and glucocorticoids	(i) At inclusion time, disease activity was not significantly differed between the different groups of smokers. (ii) At 12 months of follow-up, high disease activity was seen in 18% of current smokers, 12% of previous smokers, and 11% of never-smokers (DAS28 > 5.1, P = 0.005). (iii) At 12 months of follow-up, there was significant difference in remission within the current smokers (33%) compared to never-smokers (36%) and previous smokers (42%) (P = 0.013). (iv) The status of current smoking at inclusion time was independently predicted poor EULAR response up to 12 months of follow-up

Soderlin et al., 2012 [171]	Prospective cohort study	(i) N = 934: at starting first anti-TNF: 373 never-smokers, 345 former smokers, and 216 current smokers (ii) The average of age (Mean ± SD): 56.0 ± 12.0 years (iii) 196 were males, and 738 were females	(i) To study the effect of smoking on response to therapy, disease activity measures, and drug survival in RA patients starting their first anti-TNF drug	(i) At baseline, smoking had no effect on disease activity. Heavy smokers had the poorest drug survival. (ii) At the 3-month follow-up, current smoking was a negative predictor for EULAR response (OR = 0.53, 95%CI: 0.32-0.87, P = 0.012) and Simplified Disease Activity Index (SDAI) response (OR = 0.45, 95% CI: 0.27-0.77, P = 0.003). (iii) At 6 months, current smoking was a negative predictor for SDAI response (OR = 0.47, 95% CI: 0.25-0.88, P = 0.02). (iv) History of 11-20 pack-years was a negative predictor for SDAI at 12 months (OR = 0.30, 95% CI: 0.13-0.70, P = 0.005). (v) At 3 months of follow-up, smokers had higher visual analogue scale (VAS) global scores, CRP, and ESR levels

Sparks et al., 2016 [172]	Prospective cohort study [sample derived from Studies of the Etiology of RA (SERA)]	(i) N = 966 at baseline for cross-sectional data (262 after 2 years for prospective analysis): 542 never-smokers, 249 (1to ≤10 pack-years), 171 for > 10 pack-years (ii) The average of age (Mean ± SD): 47.2 ± 15.5 years (iii) 282 were males, and 684 were females (iv) All participants were non-Hispanic white	(i) To examine whether genetic, environmental, and serologic RA risk factors are associated with inflammatory joint signs in a cohort of first-degree relatives (FDRs) of RA patients	(i) Study found 55% of participants had at least 1 HLA shared epitope. (ii) Compared to never-smoking, smoking >10 pack-years was associated with inflammatory joint signs at baseline (OR = 1.89, 95%CI: 1.26-2.82) and at 2 years (OR = 2.66, 95% CI: 1.01-7.03). (iii) Significant interaction was observed between age and smoking in regard to risk of inflammatory joint signs (P = 0.02). (iv) Compared to FDRs who were younger than 50 years and never smoke, FDRs younger than 50 years with >10 pack-years had the highest risk of inflammatory joint signs (OR = 4.39, 95%CI: 2.22-8.66).

Stavropoulos-Kalinoglou et al., 2008 [173]	Cross-sectional study	(i) N = 392: 176 never-smokers, 147 ex-smokers, and 71 current smokers (ii) The median of age was 63.1 and with a range of 55.5-69.9 years (iii) 102 were males, and 290 were females	(i) To identify if there are any associations of smoking with body weight and composition of RA patients	(i) Bothe males and females of current smokers had significantly lower BMI and body fat (BF) when compared with the same genders of ex-smokers and never-smokers. However, fat-free mass (FFM) did not differ between groups. (ii) Current smokers had a significantly smaller waist circumference compared with ex-smokers. (iii) Smoking remained as a significant predictor for BMI (P < 0.001), BF (P < 0.05), and waist circumference (P < 0.05), even after the adjustment for age, disease duration, and HAQ score, (iv) Pack-years were inversely correlated with BF (r = -0.46; P < 0.001), and heavy smokers demonstrated lower FFM (P < 0.05)

Svensson et al., 2014 [174]	Prospective cohort study [sample from BARFOT]	(i) N = 339: 166 never, 89 former, and 84 current smokers (ii) Average of age (Mean ± SD): 56.5 ± 16 years (iii) 115 were males, and 224 were females	(i) To investigate the reproducibility of survivin status and its significance for clinical and immunological assessment of RA patients and to assess the relationship between smoking and surviving status	(i) The same levels of survivin were similar at baseline and after 24 months were seen in 79% (268), while 15% converted from positive into negative and 5% from negative into positive. (ii) Together, smoking and antibodies against cyclic citrullinated peptides (aCCP) predicted persistently high levels of survivin (OR = 4.36, 95% CI: 2.64-7.20, P < 0.001, positive predictive value 0.66, and specificity 0.83). (iii) Compared to survivin-negative, survivin positivity was associated with the progression of joint damage and significantly higher DAS28 and lower rate of remission at 24 and 60 months. (iv) Survivin status was less associated with changes in HAQ, and pain perception on Visual Analogue Scale (VAS)

Vesperini et al., 2013 [175]	Prospective cohort study [sample from ESPOIR French multicenter cohort]	(i) N =641: at inclusion time: 335 nonsmokers, 168 Ex-smokers, and 138 current smokers (ii) The average of age (Mean ± SD): 48.43 ± 12.2 years (iii) 142 were males, and 499 were females	(i) To investigate the initial response to treatment and risk of radiographic disease progression in current smokers, ex-smokers, and nonsmokers in an early RA and to analyze the influence of smoking cessation on RA outcome	(i) Baseline values (ESR and CRP) were significantly lower for current smokers than Ex-smokers and nonsmokers patients [P for ESR = 0.02, and P of CRP = 0.01]. (ii) Smoking status had no influence on HAQ score, DAS28, EULAR response, or use of disease-modifying antirheumatic drugs and biologic therapy in the first 12 months of follow-up (P > 0.05). (iii) Active smoking was associated with the adjusted risk for structural disease progression (OR = 0.50, 95%CI: 0.27-0.93, P = 0.028). (iv) At 12 months, 16 patients quit smoking; the outcome for those patients was not significantly differed from other patients.

Westhoff et al., 2008 [176]	Prospective cohort study	(i) N = 896: 648 never-smokers, 248 smokers (ii) The average of age (Mean ± SD): 56.5 ± 13 years (iii) 266 were males, and 630 were females	(i) To investigate whether smoking had an impact on disease activity, radiographic joint damage, and drug need in RF-positive and RF-negative patients with early RA	(i) Current smokers had significantly RF-positive (71%) than past smokers (66%) or never-smokers (53%), but neither the RF-positive nor the RF-negative current smokers had higher DAS28 or radiographic scores than never-smokers or past smokers. (ii) Within 3 years, current smokers had taken significantly more disease-modifying antirheumatic drugs (DMARD) combinations or biologics. (iii) Nonsmokers and those who smoked less than 20 pack-years had a 2-fold higher likelihood to reach ACR improvement than heavy smokers (smoked > 20 pack-year). (iv) Smokers and nonsmokers in the same serological group had similar radiographic joint damage

***2.B: Tobacco Smoking and Osteoarthritis* (OA) (14 Articles)**				

Amin et al., 2007 [177]	Prospective cohort study	(i) N = 159: 140 nonsmokers, and 19 current smokers (ii) Average age in years (Mean ± SD): 69 ± 9 nonsmoker, and 62 ± 9 (iii) All of 159 participants were males	(i) To assess the effects of smoking on pain and cartilage loss at knee in patients with knee OA.	(i) Once analysis controlled factors of age, BMI, and baseline cartilage, current smokers had increased risk for cartilage loss at the medial tibiofemoral joint (OR = 2.3, 95% CI: 1.0 - 5.4) and the patellofemoral joint (OR = 2.5, 95% CI: 1.1 - 5.7). (ii) Compared with nonsmokers, current smokers had significantly higher adjusted pain scores at baseline (P < 0.05) and at follow-up (P < 0.05).

Dube et al., 2016 [178]	Cross-sectional and longitudinal study [data from Osteoarthritis Initiative (OAI)]	(i) N = 2,250: 1,251 nonsmokers, and 990 smokers (current & former) (ii) Age ranged from 45 to 79 years (iii) 1,332 were males, and 918 were females	(i) To estimate the association between smoking history and symptoms/disease progression among individuals with radiographically confirmed knee Osteoarthritis (OA)	(i) In cross-sectional analyses, compared to never-smokers, current smoking was associated with greater pain (beta 0.59, 95% CI: 0.04-1.15). Also, high number of pack-years (PY) (≥ 15) was associated with slightly greater pain (beta 0.36, 95% CI: 0.01-0.71) and stiffness (beta 0.20, 95% CI: 0.03-0.37); and low PY (< 15) was associated with better joint space width (JSW) (beta 0.15, 95% CI: 0.02-0.28). (ii) In longitudinal analysis, there was no association between high or low PY or baseline smoking status with JSW at 48 months and changes in symptoms at 72 months.

Duchman et al., 2015 [179]	Prospective study	(i) N = 78,191: 63,971 nonsmokers, 6,1585 former, and 8062 were current smokers (ii) Mean of age ± SD: 67.2 ± 10.7 for nonsmokers, 68.0 ± 9.9 for former smoker, and 58.9 ± 10.6 for current smokers (iii) Percentage of female: 61.9% for nonsmokers, 53.1% for former smokers, and 55.1% for current smokers	(i) To identify 30 days' mortality and morbidity following primary total hip and total knee arthroplasty based on the smoking status and pack-year history of smoking.	(i) The risk for perioperative mortality did not differ significantly between the cohorts. (ii) Compared with the former smokers and nonsmokers (1.3%, 1.1%, respectively), the Current smokers had a higher rate of wound complications (1.8%, P < 0.001). (iii) Compared with nonsmokers and current smokers, former smokers had significantly higher rate of total complications (P < 0.001). (iv) Increasing pack-years resulted in increasing risk of total complications. Compared with patients with 0-pack-year history of smoking, the risks for total complications were increased in patients who had 1-to-20-pack-year history of smoking (OR = 1.16, 95% CI: 1.02 -1.32; P = 0.022) or a > 40-pack-year history of smoking (OR = 1.21, 95% CI: 1.00 -1.45, P = 0.045).

Floerkemeier et al., 2014 [180]	Prospective study with an average of 3.6 years' follow-up	(i) N = 533: 435 were nonsmokers, 98 were current smokers (ii) Mean of age of 49 years with a range of 18-84 (iii) 456 were males, and 337 were females	(i) To evaluate the effect of obesity and nicotine abuse on the clinical outcomes (bone nonunion and local complications) after high tibial osteotomy (HTO)	(i) Six percent of 386 interviewed patients experienced at least one local complication. (ii) The functional outcome assessed via Oxford new score was not correlated with the smoking status. However, patient's body mass indexes (BMI) below 30 were found to have higher functional score compared with patients who had BMI above 30

Johnsen et al., 2017 [181]	Prospective cohort study with randomization based on rs1051730 genotype	(i) N = 55,745: 22,843 never-smokers, 15,350 former smokers, and 16,705 current nonsmokers (ii) The average age in years (Mean) for the three groups of 0, 1, 2 rs1051730 T alleles: 49.9, 49.8, and 49.4 (iii) 29,252 were males, and 26,493 were females	(i) To determine whether the observed association between smoking and reduced risk of hip and knee osteoarthritis (OA) and subsequent joint replacement is likely to be causal	(i) In current smokers, rs1051730 T alleles were associated with reduced risk of total joint replacement (HR = 0.84, 95% CI: 0.76 – 0.98, per T allele); however such association was not observed in former smokers (HR = 0.97, 95% CI: 0.88 – 1.07) and never-smokers (HR = 0.97, 95% CI: 0.89 – 1.06).

Kalichman &. Kobyliansky, 2009 [182]	Cross-sectional study	(i) N = 827: (Smokers among OA never smoked or smoked rarely = 51, regular smokers up to 10 cigarettes = 33, and > 10 cigarettes/day = 43) (ii) Mean of age was 47.4 ± 17 for males, and was 50.8 ± 16.3 for females (iii) 443 were males, and 384 were females	(i) To estimate the prevalence of radiographic hand osteoarthritis in Chuvashian community and to investigate its associations with smoking, alcohol use, and demographics (age, and sex)	(i) 89.2% of males and 97.6% of females had at least one affected joint after age of 65. (ii) There was no significant association between the prevalence of hand OA and smoking, as well as between prevalence of hand OA and obesity in male (after adjustment for BMI); however obese females had 3 times higher risk to develop hand OA. (iii) Alcohol consumption was found to be protective on prevalence of hand OA in women

Kang et al., 2016 [183]	Cross-sectional study	(i) N = 9,064 participants received knee or hip X-rays: 1,437 current smokers, 2,155 past smokers, 1,231 indirect smokers, 4,241 nonsmokers (ii) Age: 50 or more (iii) 3,913 were males, and 5,151 were females	(i) To study the association between smoking patterns and prevalence of knee and hip joint osteoarthritis in Koreans population	(i) The prevalence of OA in Korans aged 50 or more was 13.9%, with 3.5-fold higher rate in women than that of men. (ii) Males exposed to indirect smoke had lower odd ratio for the prevalence of knee and hip joint OA than nonsmokers with similar age and sex (OR = 0.271; 95% CI 0.088-0.828)

Lampley et al., 2016 [184]	Retrospective cohort study	(i) N = 642: 359 nonsmokers 249 former smokers, and 34 current smokers (ii) Average age in years (Mean ± SD): 61.8 ± 11.2 for nonsmokers, 64.2 ± 9.1 for former smokers, 52.9 ± 9.3 for current smokers (iii) 297 were males, and 345 were females	(i) To examine the effects of cigarette smoking on complications or functional outcome scores after total ankle replacement (TAR)	(i) Compared with nonsmokers, active smokers had significantly higher risk for wound breakdown (hazard ratio [HR] 3.08, P = 0.047), but such risk was not significant for an increased rate of infection (HR 2.61, P = 0.392), revision surgery (HR 1.75, P = 0.470), and nonrevision surgery (HR 1.69, P = 0.172). (ii) The outcome scores were improved in all groups of smokers at 1- and 2-year follow-up when compared with the perioperative scores. However, the least improvement was seen among the active smokers.

Lombardi et al., 2013 [185]	Retrospective cohort study	(i) N = 498 underwent 534 hip surgery: 159 complex primary and 375 revision cases: 271 nonsmokers, and 167 former smokers, 89 current smokers, and 7 unknown smoking status (ii) The average age in years was 64 (iii) 228 were males, and 306 were females	(i) To assess the incidence and etiology of early failure in patient underwent total hip arthroplasty using ultraporous acetabular components, and examine if any preoperative variables, including smoking, related to failure.	(i) The total number of cup failures was 34 (6%): 17 infections, 14 aseptic loosening, and one each liner breakage, dislocation, and periacetabular fracture. (ii) After controlling for confounding variables, the failure rate was 10% in smokers (current, former) and 3% in nonsmokers.

Mnatzaganian et al., 2011 [186]	Prospective cohort study	(i) N = 11,388 (ii) Average age in years (Mean ± SD): 72.1 ± 4.4 with a range of 65-84 years (iii) All participants were males	(i) To examine the associations of smoking, body weight, and physical activity with risk of undergoing total joint replacement (TJR) in a population-based cohort of men.	(i) Dose-response relationships between weight (overweight increase the risk) and risk of TJR and between smoking and risk of TJR were observed (smoking increase the risk). (ii) Patients who smoked ≥ 48 years were 42-51% less likely to undergo TJR than men who had never smoked. (iii) Vigorous exercise increased the hazard of TJR; however, such hazard reached its significance only in age group of 70-74-year-old age (adjusted HR 1.64, 95% CI: 1.19-2.24).

Mnatzaganian et al., 2013 [187]	Retrospective cohort study	(i) N = 44,614 eligible participants: 41,079 nonsmokers, and 3,535 smokers (ii) Average age in years (Mean ± SD): 72.8 ± 5.0 with a range of 57-91 years. (iii) 19,860 were males, and 24,750 were females	(i) To evaluate the independent association between smoking and primary total joint replacement (TJR) for hip or knee due to osteoarthritis (OA)	(i) In both men and women, smoking was independently associated with the risk for TJR. (ii) Compared to nonsmokers, smokers were less likely to undergo a TJR (adjusted-HRs: 0.60, CI 95%: 0.48-0.75 in men, and 0.70, CI 95%: 0.56-0.86 in women). (iii) BMI was correlated in a dose-response pattern with the TJR (P < 0.001). (iv) There was no association between socioeconomic status and risk for TJR.

Niu et al., 2015 [188]	Prospective longitudinal cohort study data from Multicenter Osteoarthritis (MOST)	(i) N = 3,026 subjects with or at risk for knee OA: 1,674 were never-smokers,1,155 were former smokers, 197 were current smokers (ii) Average age in years (Mean ± SD): 62.5 ± 8.1	(i) To examine the association of smoking to radiographic knee OA and cartilage loss over four points: baseline, 30, 60, and 84 months.	(i) There was no significant relation of smoking to prevalence (baseline was 46%, 43%, and 45% among never-smokers and current and former smokers, respectively) and incidents (during 84-month follow-up were 25%, 23%, and 24% among never-smokers and current and former smokers, respectively) whole knee radiographic OA. (ii) The prevalence of any cartilage morphology (CM) lesion at baseline was 71% among never-smokers and was not higher among current or former smokers with OR 1.1 (0.5 - 2.2) and 0.9 (0.7 - 1.2), respectively. (iii) There was no significant relationship between intensity and radiographic OA outcomes as well as cartilage outcomes.

W-Dahl et al., 2007 [189]	Prospective cohort study	(i) N = 175 with knee deformity operated by hemicallotasis technique (HCO): 113 were nonsmokers/nonsnuffers (control), 21 oral snuff users, 41 were smokers (ii) Average age in years (Mean): 53 with a range of 21-75 (iii) All of 175 were males	(i) To compare the time for bone healing after HTO in three groups of snuffers, smokers, and nonsmokers/nonsnuffers subjects	(i) Compared to smokers and controls, oral snuff users had the shortest time in external fixation (87 ± 11 days for oral snuff users, 93 ± 14 days for controls, and 100 ± 25 days for smokers). The difference was significant between oral snuff users and smokers (P = 0.03). (ii) Compared with oral snuff users, smokers had 6.1 higher risks for developing complications (95% CI: 1.2-36.4)

Zhang et al., 2015 [190]	Cross-sectional study	(i) N = 3,789: 2,930 smoked 0/day; 289 smoked 1-10/day; 312 smoked 11-20/day; and 258 smoked > 20/day. (ii) Average age in years Mean (range): 53.0 ± 7.7 (iii) 1,993 were males, and 1,796 were females.	(i) To estimate the cross-sectional association between cigarette smoking and radiographic OA in Chinese population	(i) The estimated prevalence of radiographic knee OA was 28.4%. (ii) There was an inverse association between smoking and radiographic knee OA (P = 0.019). This association remained significant after adjustments for demographics (age, gender), lifestyle (physical activity, alcohol drinking, and betel quilt chewing), BMI, and educational level (P = 0.016).

***2.C: Tobacco Smoking and Spondylarthrosis (7 Articles)***				

Chung et al., 2012 [191]	Cross-sectional study	(i) N = 647 with inflammatory back pain (IBP) fulfil spondyloarthritis (SpA) criteria: 406 nonsmokers, and 241 smokers (current and former) (ii) Average age in years (Mean): 33.6 for all participants (iii) All of participants were males	(i) To investigate the relationship of smoking with clinical, functional, and imaging outcomes in patients with early axial spondyloarthritis (SpA)	(i) Study found that smoking was associated with an earlier onset of IBP (*β* = -1.46, P = 0.04), higher disease activity [(ankylosing spondylitis disease activity score, *β* = 0.20, P = 0.03); (Bath ankylosing spondylitis disease activity index, *β* = 0.50, P = 0.003)], and worse functional status (Bath ankylosing spondylitis functional index, *β* = 0.38, P = 0.02). (ii) Smoking was associated with worse outcomes: (a) high rate of MRI inflammation of the sacroiliac joints (OR = 1.57, P = 0.02) and the spine (OR = 2.33, P < 0.001), (b) more frequent MRI structural lesions of the sacroiliac joints (OR = 1.54, P = 0.03) and the spine (OR = 2.02, P = 0.01), and (c) higher modified Stoke ankylosing spondylitis spine score (*β* = 0.54, p = 0.03). (iii) Smoking was associated with poorer quality of life on Euro-quality of life questionnaire and short forms 36 score (physical and mental) (all P < 0.001)

Mattey et al., 2011 [192]	Cross-sectional study	(i) N = 612 subject with AS: 308 never-smokers, 298 ever smoker (127 current, 172 former), 6 no data about smoking (ii) Average age in years was 51.0 with a range of 42-60 years (iii) 443 were males, and 169 were females	(i) To examine the association between smoking and parameters related to ankylosing spondylitis (AS) (disease activity, pain, function, and quality of life)	(i) Compared to never-smokers, ever-smokers had higher scores of Bath AS Functional Index (BASFI) (P < 0.0001), pain (P = 0.04), AS quality of life questionnaire (ASQoL) (P = 0.003), and the AS quality of life measures (EASi-QoL) (P < 0.02).

Munk et al., 2016 [193]	Cross-sectional study	(i) N = 297 with axial spondyloarthritis (SpA) [n = 110, 36% smokers] or psoriatic arthritis (PsA) [n = 101, 35% smokers], and control [n = 96] (ii) The average of age for SpA, PsA, and control was 36.6, 37, and 41.5 year, respectively. (iii) 187 were males, and 110 were females	(i) To evaluate the association between type II collagen (turnover marker) and disease profile in patients with axial spondyloarthritis (SpA) and psoriatic arthritis (PsA) based on their smoking status	(i) In PsA, procollagen IIA N-terminal peptide (PIIANP) and matrix metalloproteinase-generated type II collagen fragment (C2M) did not differ according to smoking and HLA-B27, while, in SpA, PIIANP was higher in HLA-B27 positive (P = 0.03), and C2M was lower in smokers (P = 0.02). (ii) The PsA patient's level of PIIANP and C2M did not differ from the controls. Meanwhile, DMARD-naïve SpA patients had higher PIIANP (P = 0.01) and C2M (P = 0.0007)

Poddubnyy et al., 2012 [194]	Prospective cohort study	(i) N = 210 axial SpA: 147 nonsmokers, and 63 current smokers (ii) Average of age in years (Mean ± SD): 37.1 ± 10.6 (iii) 107 were males, and 103 were females	(i) To assess the rates and to explore factors predicting spinal radiographic progression over 2 years in patients with SpA	(i) After 2 years 14.3% of patients showed radiographic progression (20% of those with AS, and 7.4% of those with nonradiographic axial SpA). (ii) Factors that were independently associated with spinal radiographic progression were as follows: presence of syndesmophytes at baseline (OR = 6.29, P < 0.001), ESR (OR = 4.04, P = 0.001), CRP (OR = 3.81, P = 0.001), and cigarette smoking (OR = 2.75, P = 0.012).

Sakellariou et al., 2015 [195]	Cross-sectional study	(i) N = 106 of AS subjects who were on tumor necrosis factor inhibitor: 22 never, 28 former, and 56 current smokers (ii) Average age in years (Mean ± SD): 41.5 ± 12.7 (iii) 100 were males, and 6 were females	(i) To investigate the association between smoking and clinical, inflammatory and radiographic parameters in patients with ankylosing spondylitis (AS).	(i) Compared to never-smokers, ever-smokers had significantly higher Bath AS Functional Index (BASFI) (P = 0.035) and a trend for higher modified Stroke AS Spine Score (mSASSS), while current smokers had significantly higher Bath AS Disease Activity Index (BASDAI) (P < 0.001) and a trend for higher BASFI (P = 0.059), when compared with nonsmokers (never and former smokers). (ii) The intensity of smoking (pack-years) was positively associated with BASFI (r = 0.443, P < 0.001) and mSASSS (r = 0.683, P < 0.001) and duration of inflammatory back pain (r = 0.628, P < 0.001).

Sakellariou et al., 2017 [196]	Cross-sectional study	(i) N = 91 subjects (57 of AS patients on TNF inhibitor, and 34 matched control by sex, age, and BMI): (current smokers: 29 in AS, and 17 in control), (ever-smokers: 45 in AS, and 27 in control) (ii) Average age in years (Mean ± SD): 39.1 ± 1.4 for AS patients, and 38.8 ± 1.0 for control (iii) 85 were males, and 6 were females	(i) To compare level of serum Dickkopf-1 (Dkk-1), sclerostin, and vascular endothelial growth factor (VEGF) of AS patients with healthy controls and to assess their association with smoking, clinical, inflammatory, and radiographic parameters	(i) Serum bone markers were not significantly differed between AS patients and controls. (ii) The high level of Dkk-1 was significantly associated with elevated ESR and CRP, high level of sclerostin, and no syndesmophytes (All P ≤ 0.001). (iii) The high level of VEGF was significantly associated with ever-smoking and elevated ESR and CRP (All P < 0.05)

Wang et al., 2012 [197]	Case-control study	(i) N = 348:172 had cervical spondylotic myelopathy (CSM), and 176 matched control by age and sex: group I (nonsmokers), group II (< 100), group III (101-300), and group IV (> 300). (ii) Average age in years Mean (range): 47.7 ± 7.5 for cases, and for 47.1 ± 6.9 control (iii) 184 were males (96 in cases, 88 in controls), and 164 were females (176 in cases, and 88 in controls)	(i) To investigate the association of collagen IX tryptophan (Trp) alleles (Trp2 and Trp3) and smoking with cervical spondylotic myelopathy (CSM) Chinese patients	(i) Compared to control, the CSM cases had a significantly higher prevalence of Trp2 alleles (Trp2+) (19.8 versus 6.2%, P = 0.002), but not Trp3 alleles (Trp3+) (23.3 versus 21.6%, P = 0.713). (ii) In subjects with Trp2+, smoking < 100 cigarettes was not associated with increased risk for CSM (OR = 1.34, 95% CI = 0.85-2.18, P > 0.05), when smoked cigarettes were between 101 and 300, the risk for CSM reached 3.34 (95%CI = 2.11-5.67, P = 0.011); and such risk was increased further with cigarettes > 300 (OR = 5.56, 95%CI = 3.62-7.36, P < 0.001). (iii) In subject with Trp2-, smoking > 300 cigarettes increased the risk for CSM (OR= 2.14, 95%CI = 1.15-4.07, P = 0.024).

***2.D: Tobacco Smoking and Temporomandibular Joint Disorders (TMD) (4 Articles)***				

Melis et al., 2010 [198]	Prospective study	(i) N = 352: 314 nonsmokers, 38 smokers (38 light smokers, 12 moderate smokers, and 11 heavy smokers)	(i) To examine the effect of cigarette smoking on pain intensity in TMD patients.	(i) Compared to nonsmokers, smokers had significantly higher overall TMD pain intensity (P = 0.007). Also, such differences were found to be particularly significant when nonsmokers compared with heavy smokers (P = 0.004). (ii) There was positive correlation between cigarettes smoked/day and pain intensity for whole group (P < 0.0001) and the group of females (P = 0.001), but such association was not observed in group of males.

Miettinen et al., 2017 [199]	Cross-sectional study	(i) N = 8,678: 5,238 nonsmokers, 1,114 (1-5 cigarettes/day), 2,059 (10-20 cigarettes/day), and 222 (20+ cigarettes/day) (ii) The average of age was 19.6 years (iii) 8,530 were males, and 148 were females	(i) To evaluate the prevalence of TMD symptoms and their associations with smoking or alcohol consumption in Finnish adults	(i) In male population, the prevalence of occasional TMD symptoms varied between 5.8% (difficulty in jaw opening) and 27.8% (temporomandibular joint [TMJ] clicking). In female population, there was a high prevalence for all symptoms with an exception for TMJ clicking. (ii) Smoking was significantly associated with TMD symptoms, except TMJ clicking. Also, use of snuff was associated with facial pain. (iii) There was significant association between alcohol consumption (at least once a week) with TMJ clicking, facial pain, and TMJ pain with or without jaw movement

Sanders et al., 2012 [200]	Case-control study	(i) N = 299: 227 nonsmokers, 44 former smokers, and 28 current smokers (ii) The range of age was 18-60 (iii) All participants were females	(i) To investigate whether age modified an effect of smoking on temporomandibular disorder (TMD) in female population and investigate the extent to which this relationship was explained by psychological profile, inflammatory response, and allergy	(i) Compared with nonsmokers, ever-smokers aged less than 30 years had higher odds of TMD (OR = 4.14, 95% Cl: 1.57-11.35) than older adults (OR = 1.23, 95% Cl: 0.55-2.78) (P (effect modification) = .038). (ii) The effect was attenuated by 45% to nonsignificant level when study adjusted for psychological profile, cytokines, and history of allergy-like conditions.

Weingarten et al., 2009 [201]	Retrospective case series study	(i) N = 606: 359 nontobacco users, 153 former tobacco users, 91 current tobacco users, and 3 no data (ii) Average age in years (Mean ± SD): 44 ± 17 for nonusers, 51 ± 16 former users, and 36 ± 12 for current users (iii) 106 were males, and 497 were females	(i) To examine the joint disorders (TMD) symptoms and current tobacco use in patients evaluated at a specialized orofacial pain clinic	(i) Compared to nonusers, tobacco users were more likely to have moderate to severe pain interference (OR = 1.94, 95% CI: 1.16-3.22). Such difference did not remain significant after adjustment for patient's demographics. (ii) When analysis was conducted in subjects who lacked a myofascial pain (N = 333), the difference remained significant after adjustment (adjusted OR = 4.56, 95% CI: 1.46-14.24)

**Section 3: Muscles: Tobacco Smoking and Skeletal Muscles (20 Articles)**				

Adedoyin et al., 2010 [202]	Pretest-posttest design	(i) N = 104: 52 nonsmokers = 52 smokers (ii) The average of age: 23.2 ± 2.37 for nonsmokers, 23.1 ± 2.9 for smokers (iii) All participants were males	(i) To compare back extensor muscles' strength (BEMS) before and after a fatigue induction in smoking and nonsmoking male athletes	(i) Compared to nonsmokers, athletes smokers demonstrated higher rate of perceived exertion (P = 0.007) and fatigue index (10.76% versus 5.07%) and significant reduction in BEMS following fatigue induction (P < 0.05)

Al-Obaidi et al., 2014 [203]	Comparative nonequivalent quasi-experimental design	(i) N = 177: 66 nonsmokers = 111 smokers (ii) The average of age (Mean ± SD): 34.2 ± 9.7 with a range of 20-59 years (iii) All participants were males	(i) To investigate whether smoking had an effect on grip-strength and fatigue resistance and to investigate if fatigue index can be predicted by demographic data, duration of smoking, packets smoked-per-day, and physical activity	(i) Compared with nonsmokers, smokers exhibited significantly lower number of repetitions to squeeze the soft rubber ball to induce fatigue (t = 10.6, P < 0.001 for dominant hand; t = 13.9, P < 0.001 for nondominant hand). Also, smokers demonstrated significantly higher fatigue index than nonsmokers (t = -8.7, P < 0.001 for dominant hand; t = -6.0, P < 0.001 for nondominant hand). (ii) Compared to nonsmokers, smokers had significant difference in maximum isometric grip-strength (MIGS) scores after induced fatigue (beta = -3.98, standard error = 0.59, P < 0.001). (iii) This study found smoking status was the strongest significant predictor of the fatigue index.

de Oca et al., 2008 [204]	Cross-sectional study	(i) N = 34: 14 non-COPD smokers, 20 healthy controls (ii) The average of age (Mean ± SD): 55 ± 8 for non-COPD smokers, 58 ± 7 for healthy control	(i) To assess the effect of smoking on the skeletal muscles	(i) The cross-sectional area of smoker muscles demonstrated a decreased level of type I and type Ii fibers. (ii) Lactate dehydrogenase levels and the percentage of fibers were increased in smokers. (iii) Both endothelial nitric oxide synthase (eNOS) (38.9 ± 11.0 versus 45.2 ± 7.7 ng/mg protein; P < 0.05) and neuronal endothelial nitric oxide synthase (nNOS) (96.9 ± 11.7 versus 125.4 ± 31.9 ng/mg protein; P < 0.01) were lower in smokers than in nonsmokers

Kim & Kim, 2012 [205]	Pretest-posttest design	(i) N = 31: 16 nonsmokers, 15 smokers (ii) Average age in years (Mean ± SD): 23.00 ± 2.82 for nonsmokers, 22.60 ± 2.29 for smokers	(i) To examine whether smoking had an effect on internal oblique (IO) and transversus abdominis (TrA) muscles	(i) There were significant differences found between both groups of smokers and nonsmokers in regard to the percentage of change in muscle thickness (PCMT) of the TrA and in the relative contribution ratio (RCR) of both TrA and IO. (ii) Compared to nonsmokers, smokers were more dependent on IO than TrA during forceful expiratory conditions

Kimura et al., 2007 [206]	Pretest-posttest design	(i) N = 16: 10 nonsmokers, and 6 smokers (smoking 10-20 cigarettes over last 10 years) (ii) Average age in years (Mean ± SD): 48.7 ± 4.4 for nonsmokers, and 50.3 ± 4.0 for smokers (iii) All subject were males	(i) To investigate influence of chronic moderate smoking on physical fitness and local muscle (vastus lateralis) oxygenation profile during incremental exercise	(i) Both groups of smokers and nonsmokers had similar level of venous oxygen (VO2) and work rate at the level of 2 mmol/L lactate or 80%HR(max) (P < 0.05). (ii) During incremental exercise, 5 out of 6 smokers demonstrate a decrease in O2Hb throughout the incremental exercise, but 8 out of the 10 nonsmokers demonstrated a gradual increase of O2Hb.

Kok et al., 2012 [207]	Prospective cohort study [Subjects from AGHLS study]	(i) N = 4 assessments: at age 21 (n = 181: 68 smokers), 27 (n = 144: 43 smokers), 32 (n = 426: 84 smokers) and 36 (n = 373:93 smokers) (ii) The range of age was 21-36 (iii) 156 were smokers males, and 142 were smoker females	(i) To examine longitudinally whether smoking tobacco is correlated with muscle strength	(i) In both men and women there was an inverse relationship between tobacco smoking and Knee muscle strength (KMS). (ii) In men, there was a 2.9% reduction in the KMS for each 100 g of smoked tobacco, while, in women, there was a 5% reduction in the KMS for each 100 g of smoked tobacco. (iii) The relationship between the smoked tobacco and KMS existed largely and independently of lifestyle covariates (physical activity and dietary intakes)

Kumar et al., 2010 [208]	Pretest-posttest design	(i) N = 120: 60 nonsmokers, 60 smokers (ii) Average age in years Mean (range): 35 ± 5 years (iii) All participants were males	(i) To determine the isometric lumbar extensor strength before and after fatigue challenge among smokers and nonsmokers	(i) There was a confirmed relationship between reduced lumbar extensor strength and cigarette smoking. (ii) The negative impact of smoking on lumbar extensors suggests increased susceptibility to lumbar injuries and thereby low back pain

Lee et al., 2013 [209]	Cross-sectional study	(i) N = 30 teenagers: 15 nonsmokers, and 15 smokers (ii) The average of age was 13.03 years	(i) To assess effect of smoking on teenagers' (IO) and (TrA) expiratory muscles	(i) No significant differences were found between both groups of smokers and nonsmokers in the thickness, PCMT, and RCR of both the IO and TrA muscles. (ii) Compared to nonsmokers, smokers had significantly lower forced expiratory volume at one second (FEV1) (3.74 ± 0.65 versus 4.33 ± 0.72, P = 0.03) and peak expiratory flow (PEF) (5.89 ± 1.62 versus 7.73 ± 1.56, P < 0.01).

Morse et al., 2007 [210]	Pretest-posttest design	(i) N = 19: 9 male smokers, and 10 male activity-matched nonsmokers (ii) The average of age (Mean ± SD): 22.2 ± 2.5 smokers, 25.4 ± 2.9 nonsmokers (iii) All participants were males	(i) To determine whether muscle function (quadriceps muscle mass and contractile properties) is compromised in male healthy smokers in comparison with activity-matched nonsmokers	(i) The healthy male smokers and nonsmokers were found to be similar in regard to the maximal voluntary contraction torque, quadriceps anatomical cross-sectional area, knee extensor torque/quadriceps cross-sectional area, activation, coactivation, and force-frequency relationship. (ii) Compared to nonsmokers, smokers were found to have 17% lower fatigue index

Neves et al., 2016 [211]	Pretest-posttest design	(i) N = 40 healthy males: 20 smokers, and 20 control nonsmokers (ii) The average of age (Mean ± SD): 34.35 ± 6.45 smokers, 34.30 ± 5.92 nonsmokers (iii) All participants were males	(i) To investigate whether cigarette smoking is associated with proinflammatory cytokines, oxidative stress, and changes in muscular and physical performances	(i) Smokers showed an increase in the oxidative stress represented by an increase in the levels of thiobarbituric acid reactive substances (TBARS) and a decrease in the total antioxidant capacity of plasma and catalase level (P < 0.05). Also, one of the inflammatory markers (sTNFR1) was increase in the P < 0.05. (ii) Smokers showed a decrease in the total work which reflects skeletal muscle dysfunction (P < 0.05) (iii) IL-6, IL-10, sTNFR2, superoxide dismutase (SOD), peak torque, VO2 peak, HRmax, and walking distance were similar between groups

Orozco-Levi et al., 2012 [212]	Case-control study	(i) N = 21: 14 male COPD patients who were smokers, 7 healthy nonsmoker controls (ii) The average of age (Mean ± SD) was 67 ± 7 (iii) All participants were males	(i) To investigate whether the presence of chronic obstructive pulmonary disease (COPD) is associated with peripheral muscle injury	(i) Both groups of control and COPD patients were found to have signs of injury in their skeletal muscles, and such injury was not only shown in cases showing severe airflow obstruction but also in the mild or moderate stages of the disease. (ii) There was a significant association between current smoking and presence of COPD with the increased injury of the muscle as evidenced by the finding of electron microscopy techniques.

Patel et al., 2016 [213]	Cross-sectional study	(i) N = 87: 61 COPD including current and former smokers, 13 smokers had normal spirometry "healthy Smokers", and 13 never-smoking controls (ii) The average of age (Mean ± SD): 64 ± 10 COPD, 51 ± 7 healthy smokers, and 65 ± 8 for never-smokers (iii) 53 were males, and 34 were females	(i) To evaluate skeletal muscle and Klotho protein in smokers and COPD patients and to investigate the relationships between Klotho and skeletal muscle parameters	(i) Quadriceps Klotho levels were lower in current smokers regardless of their spirometry (P = 0.01) but were not lower in COPD patients. (ii) Current smoking was the only independent factor associated with the quadriceps Klotho levels. (iii) Serum Klotho level was independently associated with quadriceps strength, but it was not related to quadriceps Klotho levels or to the spirometric parameters

Puthucheary et al., 2015 [214]	Prospective cohort study	(i) N = 215 healthy army recruits: 142 nonsmokers, 61 Ex-smokers, 11 current smokers (ii) The average of age (Mean ± SD): 20.0 ± 2.3 (iii) All participants were males	(i) To investigate the relationships between short exercise-training period and lower limb and bone and muscle. Also, to investigate the influence of smoking status on this relation.	(i) Pretraining, femoral bone, and rectus femoris volumes (RFVOL) were smaller in smokers than in nonsmokers (100.9 ± 20.2 versus 108.7 ± 24.5, P = 0.018 for dominant limb; 96.2 ± 16.9 versus 104.8 ± 21.3, P = 0.002 for nondominant limb), although RFVOL increases significantly with training (P < 0.001). (ii) Pretraining RFVOL was correlated to bone cortical volume (r2 = 0.21 for dominant, and 0.30 for nondominant leg, P < 0.001) and specifically to periosteal (r2 = 0.21 for dominant, and 0.23 for nondominant leg, p < 0.001) volume. (iii) Pretraining dominant RFVOL was independently associated with total hip bone mineral density (P < 0.001).

Rapuri et al., 2007 [215]	Cross-sectional study	(i) N = 487 elderly women: 268 nonsmokers, 163 former smokers, and 56 current smokers (ii) The average of age (Mean ± SD): 71.7 ± 0.22 nonsmokers, 71.4 ± 0.29 former smokers, 70.6 ± 0.42 current smokers (iii) All participants were females	(i) To examine whether smoking is associated with physical performance and if this association was mediated through smoking effect on vitamin D or estrogen metabolism	(i) Current smokers had significantly slower timed rise and timed walk tests and had decreased grip-strength than those of former or nonsmokers (all P < 0.05). (ii) The predictors for physical performance measures were smoking, age, total body fat, and serum 1, 25(OH) (2) D. (iii) The estimated effect of current smoking on physical performance was equivalent to 7-11 years of age-related drop in physical performance.

Rhee & Kim, 2017 [216]	Pretest-posttest design	(i) N = 30: 15 nonsmokers, 15 smokers (ii) The average of age (Mean ± SD): 30.07 ± 4.46 for nonsmokers, and 27.13 ± 3.31 for smokers (iii) All participant were males	(i) To compare the differences in the activation of abdominal muscles during trunk extension between smokers and nonsmokers	(i) There were no significant differences in the maximal voluntary isometric contraction (% MVIC) of rectus abdominis and external oblique between smokers and nonsmokers. However, the % MVIC of internal oblique and transversus abdominis was significantly greater in smokers than in nonsmokers

Rom et al., 2015a [217]	Cross-sectional study	(i) N = 49 heavy smokers with an average of 31.4 ± 23.3 pack-years (ii) The average of age (Mean ± SD): 44 ± 12 (iii) 23 were males, and 26 were females	(i) To examine the association between pack-years, body composition (BC), metabolic and inflammatory markers, and muscle strength in heavy smokers.	(i) Pack-years were positively correlated with fasting blood sugar, ALP, and CRP levels. (ii) Pack-years were positively correlated with waist circumference, BMI, whole-body, and trunk fat mass measured by both DEXA and BIA. (iii) There was a negative correlation between pack-years and muscle strength. (iv) Pack-years were found to be only correlated with CRP levels after the adjustment for age, sex, and BMI.

Rom et al., 2015b [218]	Prospective cohort study	(i) N = 81 adult participating in smoking cessation program combining group counselling and varenicline treatment: 41 who completed measurement (31 continued smoking, and 10 quitters). (ii) The average of age: 44 ± 12 for those completed the measurements (iii) 22 were males ( 17 continued, 5 quitters), and 19 were females (14 continued, 5 quitters)	(i) To investigate the effects of smoking cessation on body composition and muscle strength	(i) Compared to subject continued smoking, all quitter's parameters of body composition and muscle strength were increased. (ii) The adjusted differences [95%CI)] between quitters and smokers were as follows: body weight 4.43kg (1.56-7.31kg); lean mass 1.26kg (0.24-2.28kg); fat mass 3.15kg (0.91-5.39kg); BMC 48.76g (12.06-85.54g); BMD 0.024g/cm2 (0.004-0.043g/cm2); handgrip strength 3.6kg (1.12-6.08kg); predicted one-repetition maximum of chest press 7.85kg (1.93-13.76kg); and predicted one-repetition maximum of leg press 17.02kg (7.29-26.75kg).

van den Borst et al., 2011[219]	Prospective cohort study [subject from Health, Ageing and Body Composition (ABC) Study]	(i) N = :260 with obstructive lung disease (OLD), 1,914 control with normal lung function [157 smokers, 866 former smokers, and 891 never-smokers] (ii) At baseline the average of age (Mean ± SD): 74 ± 3 (iii) 1,059 were males, and 1,115were females	(i) To examine if smoking and OLD accelerate the ageing-related decline in lean mass and physical functioning.	(i) At baseline, participants with OLD and smoking controls had a significantly lower weight, fat mass, lean mass and BMC than that of never-smoking (P < 0.05). (ii) There was no differences in the loss of weight, fat mass, lean mass and strength between patients with OLD and never-smoking controls. (iii) Short Physical Performance Battery declined 0.12 points/year faster in men with OLD, (P = 0.01), while in BMC declined 4 g/year faster women with OLD, (P = 0.02). (iv) Compared with never-smoking controls, smoking controls lean mass declined 0.1 kg/year faster in women (P = 0.03) and BMC 8 g/year faster in men (P = 0.02).

Wust et al., 2008a [220]	Pretest-posttest study	(i) N = 10: 5 smokers (smoked on average15 ± 9 cigarettes/day) and 5 nonsmokers (never smoked) (ii) The average of age (Median, Range): (40, 25-72) for smokers, and (45, 23-72) for nonsmokers (iii) 4 were males, and 6 were females	(i) To investigate whether the lower fatigue resistance in smokers than in nonsmokers is caused by a compromised muscle oxidative metabolism	(i) The vastus lateralis of both smokers and nonsmokers had similar level of capillarization, myoglobin concentration, and succinate dehydrogenase (SDH) activity. (ii) In nonsmokers, there was a relationship between fatigue resistance and SDH activity (r = 0.93; P = 0.02), however; such relationship was absent in smokers (r =0.67; P = 0.21)

Wust et al., 2008 [221]	Pretest-posttest study	(i) N = 85: 40 smokers matched with 45 nonsmokers (ii) The average of age was 38.87 with a range of 18-73 years (iii) 41 were males (19 in smokers, and 22 in nonsmokers), and 44 were females (21 in smokers, and 23 in nonsmokers)	(i) To study the relationship between smoking history and skeletal muscle (quadriceps) function and fatigue resistance	(i) There were no significant differences in maximal strength and isometric contractile speed between smokers and nonsmokers. (ii) Compared with nonsmokers, smokers had greater muscle fatigue (P = 0.014). (iii) The muscle fatigue among the smokers did not correlate with pack-years (r = 0.094, P = 0.615), number of daily cigarettes (r = 10.092, P = 0.628), respiratory function (r = -0.187, P = 0.416), or physical activity (r = -0.029, P = 0.877).

**Section 4: Cartilage (19 Articles)**				

***4.A: Tobacco Smoking and Knee Joint Cartilage (7 Articles*)**				

Balain et al., 2012 [222]	Retrospective cohort study	(i) N =193 out of 316 patients responded after 2 round of questionnaire: 158 nonsmokers, 35 smokers (ii) The average of age was 40.6 with a range of 16-76 years (iii) 149 were males, and 44 were females	(i) To determine the effect of compartment location, concomitant arthroscopic surgery and smoking on the medium-term outcome (mean of 37 months) of knee microfracture	(i) Overall the satisfaction rates in regard to the outcomes were found in 72% of the patients. (ii) Compared with patients with an affected medial compartment, patients with an affected lateral or patellofemoral compartment had larger improvement (1.5 SD versus 1 SD). However, their satisfaction rates did not differ significantly. (iii) Having concomitant knee surgery did not significantly affect the functional outcome or satisfaction rate. (iv) Smoking was associated with a lower satisfaction rate (54 for smokers versus 76% for nonsmokers), but it did not affect the outcome of microfracture

Blackwell et al., 2016 [223]	Retrospective chart review cohort study	(i) N = 104: 52 nonsmokers, and 52 current smokers matched in age, sex, and ACL status (ii) Average age in years (Mean ± SD): 28.6 ± 8.9 (iii) 64 were males, and 40 were female	(i) Compare the risk of early meniscus repair failure between the current smokers and nonsmokers	(i) Meniscus repair failure occurred more significantly in smokers (15 failures in 56 menisci, 27% failure) than in nonsmokers (4 failures in 56 menisci, 7% failure) (P = 0.0076).

Davies-Tuck et al., 2009 [224]	Prospective cohort study	(i) N = 271: 167 current nonsmokers, and 104 smokers (current or former) (ii) Average age in years (Mean ± SD): 57.6 ± 4.8 for nonsmokers, and 58.2 ± 5.8 for smokers (iii) 102 were males, and 169 were females	(i) To investigate effect of smoking on the tibial and patella cartilage and the risk for development or persistence of bone marrow lesions (BMLs)	(i) Being a smoker was associated with increased annual loss of medial (Difference = 13.4 *μ*l, P = 0.03), but not lateral (Difference = 4.86 *μ*l, P = 0.45), or patella cartilage volume (Difference = -2.57 *μ*l, P = 0.79). (ii) Increasing smoked pack-years were associated with increased medial cartilage volume loss (P = 0.04). (iii) Compared to never-smokers, the BMLs among ever-smokers were 11.4 more likely to persist over 2 years [95% CI: 1.54 - 89.9; P = 0.02]

Ding et al., 2007 [225]	Case-control study	(i) N = 325: 162 subject with at least 1 parent with severe primary knee OA and 163 control (ii) 162 subject: 75 never-smokers, 47 former smokers, and 40 current smokers (iii) 163 control: 95 never-smokers, 38 former smokers, and 30 current smokers. (iv) Mean of age was 45 years and with a range of 26-61. (v) 135 were males, and 190 were females	(i) To describe the effect of smoking on the change in knee cartilage volume and defects and to test for interaction between smoking and family history of OA	(i) Smoking was associated with annual change in medial and lateral tibial cartilage volume. (ii) Compared with never-smokers, current smokers had significantly higher risk for medial and lateral tibiofemoral cartilage defect (OR: 4.91 versus 2.98, P < 0.05), and such risk was increased with the amount of smoking (OR = 9.90 for heavy smoking, and OR = 12.98 for never-smoking, all P < 0.05). However, such findings were not reported among control group except for change in lateral tibial cartilage volume. (iii) Smoking had significant interaction with offspring status for change in medial tibial cartilage volume (P = 0.047) and increases in medial and lateral tibiofemoral cartilage defects (P = 0.03, P = 0.049, respectively)

Gungor et al., 2016 [226]	Cross-sectional study	(i) N = 88: 45 nonsmokers, and 43 smokers (ii) Average age in years (Mean ± SD): 35.9 ± 8.0, with a range of 18-56 (iii) 71 were males, and 17 were females	(i) To assess the impact of cigarette smoking on sonoelastographic properties of distal femoral cartilage in asymptomatic adults	(i) Compared to nonsmokers, smokers had significantly higher thickness for the femoral medial, intercondylar, and lateral cartilage (P = 0.002, P = 0.017, and P = 0.004, respectively). (ii) Compared with the nonsmokers, smokers had lower strain ratio for the medial distal femoral cartilage (P = 0.003). (iii) There was significant positive association between amounts of smoking and cartilage thicknesses (P < 0.05). (iv) There was significant negative association between amounts of smoking and medial cartilage strain ratios (P < 0.05).

Jaiswal et al., 2009 [227]	Case-control study	(i) N = 129: 66 nonsmokers, 15 Ex-smokers, and 48 smokers (ii) Average age in years Mean (range): 33.7 years (14-50) for nonsmokers, for Ex-smokers 36.4 years (20-49), and 33.4 years (18-50) for smokers (iii) 65 were males, and 64 were females	(i) To assess effect of smoking on the outcome of autologous chondrocyte implantation for that used to treat patient's knee suffered from full-thickness chondral defects	(i) Compared to nonsmokers, smokers had significantly lower mean of Modified Cincinnati Knee score before (P = 0.037) and 2 years after surgery (P < 0.05). (ii) After 2 years of follow-up, smokers had significant improvement in the knee score compared to that of nonsmokers (P < 0.05). Graft failure was only reported on the smokers (P = 0.016). (iii) Strong negative correlation was observed between the number of cigarettes smoked and the outcome following surgery (r = -0.65, P = 0.004)

Racunica et al., 2007 [228]	Prospective cohort study	(i) N = 297: 179 were nonsmokers, 118 ever-smokers (ii) Average age in years (Mean ± SD): 58.0 ± 5.5 (iii) 111 were males, and 186 were females	(i) To examine the association between tobacco use and tibial cartilage volume and tibiofemoral cartilage defects in healthy adult population.	(i) The volume of tibial cartilage was positively associated with subjects who ever smoked (P = 0.02) as well as pack-years smoked (P = 0.03). However, the study found no association between smoking and presence of tibiofemoral cartilage defects.

***4.B: Tobacco Smoking and Spinal Cartilage (12 Articles)***				

Appaduray et al., 2013 [229]	Retrospective cohort study	(i) N = 902: 40 patients were diabetic and had positive smoking history, 75 with diabetes, 343 with smoking history, and nonsmokers, and 444 control group (ii) Average of age was 55 ± 17.7 years	(i) To determine the effects of diabetes and smoking on the outcomes of lumbar spinal surgery	(i) Diabetes was significantly associated with an increased risk of developing single (P = 0.007) or multiple complication (P = 0.008), infectious complication (P = 0.015), and cardiovascular complications (P = 0.015). However, the history of positive smoking was not found to increase the rate of poor outcome.

Bydon, et al., 2015 [230]	Retrospective cohort study	(i) N = 500 who underwent laminectomy at 1, 2, or 3 levels (ii) 284 were males (238 males were nonreoperation group and 46 were in reoperation group), and 216 were females (181 females were nonreoperation group and 35 were in reoperation group)	(i) To identify the predictors of an increased risk for reoperation in patients who had underwent lumbar laminectomy	(i) Out of 500, 81 patients (16.2%) developed subsequent spinal disorders that required reoperation. (ii) Smoking was an independent predictor for all reoperations (OR 2.15, P = 0.01) including reoperation after single level (OR 11.3, P = 0.02) and multilevel and after a multilevel (2 or 3 levels) laminectomy (OR 1.98, P = 0.05), but not for nine patients who underwent nondegenerative conditions. (iii) Also, smoking was a significant predictor for reoperation in 72 with spinal degeneration (OR 2.06, p = 0.04), but it was not a significant predictor for 9 patients that underwent reoperation for nondegenerative conditions

Eubanks et al., 2011 [231]	Retrospective cohort study	(i) N = 158: 117 were nonsmokers, 41 were smokers (ii) Mean of age was 61 with a range of 35-87 years (iii) 93 were males, and 65 were females	(i) To assess whether smoking reduces the fusion rate in patients that underwent posterior cervical fusion with lateral mass instrumentation and iliac crest bone grafting, and if it is associated with increased pain, decreased activity level, and decreased rate of return of work	(i) The fusion rate (100%) was similar in both groups of smokers and nonsmokers. However, 80% of patients had Grade I or II in Odom Criteria used to assess the clinical outcome. (ii) Smokers were 5 times more likely to develop advance grades (Grade III or IV) and usually had huge limitation in their physical activities. (iii) The fusion rate was not influenced by diagnosis, age, and gender

Glassman et al., 2007 [232]	Retrospective cohort study	(i) N = 148: 117 were nonsmokers, 41 were smokers (ii) Mean of age was 51.1 years with a range of 18-78. (iii) 71 were males, and 77 were females	(i) To investigate whether smoking influences fusion rate and clinical outcome in patients that underwent rhBMP-2 matrix (AMPLIFY) or iliac crest bone graft for single-level lumbar fusion	(i) The fusion rate was 100% in all 55 nonsmokers in the rhBMP-2 group, while it was seen in 20 out of 21 smokers in the rhBMP-2 group (95.2%). (ii) In the groups of iliac crest bone graft (ICBG), the fusion rates among smokers was 94.1% (48 out of 51), while it was 76.2% (16 out of 21) among the smokers. (iii) At every postoperative interval, statistically significant improvement from baseline was observed for Oswestry Disability Index (ODI) and SF-36 Physical Component score measures in both smokers and nonsmokers.

Gulati et al., 2015 [233]	Multicenter prospective cohort study [NORspine]	(i) N = 825: 619 were nonsmokers, 206 were smokers (ii) Mean of age was 67.6 for nonsmokers and it was 62.9 for the smokers (iii) 417 were males, and 408 were females (301 nonsmoker, and 100 in smoker groups)	(i) To examine relationships between daily smoking and patient-reported outcome at 1 year using Oswestry Disability Index (ODI), length of hospital stay, and perioperative complications after microdecompression for single- and two-level central lumbar spinal stenosis (LSS)	(i) ODI changes at 1 year were significantly different between nonsmokers and smokers (4.2 points, 95% CI 0.98-7.34, P = 0.010). (ii) Smokers were found to be different than nonsmokers in terms of hospital length of stay at either single level (2.3 versus 2.2 days, p = 0.99) or two levels (3.1 versus 2.3 days, p = 0.175), and in regard to the overall complication rate (11.6% versus 9.2%, p = 0.34)

Hermann et al., 2016 [234]	Prospective cohort study	(i) N = 50: 34 were nonsmokers, 16 were smokers (ii) Mean of age was 58 years with a range of 29-81 in nonsmokers and it was 47 years with a range of 29-75 in smokers. (iii) 23 were males, and 27 were females	(i) To assess whether smokers yield worse results concerning lumbar interbody fusion than nonsmokers	(i) Compared to the smokers, nonsmokers had significantly higher rate of fusion (P = 0.01). (ii) There were no differences in the in ODI improvement (P = 0.93) and pain reduction (P = 0.54) between smokers and nonsmokers. (iii) One year after the surgery, the intake of opioids was only reduced in nonsmokers from 32.35% to 29.41%. However, it remained unchanged among the smokers (43.75%)

Lau et al., 2014 [235]	Retrospective cohort study	(i) N = 160: 79 were nonsmokers (never smoke), 41 were quitters (stopped for at least 1 year), and 40 were current smokers (ii) Mean of age (mean ± SD): 52.2 ± 11.3 for nonsmokers, 55.0 ± 11.7 for quitters, and 52.0 ± 12.0 for never-smokers (iii) 93 were males, and 67 were females	(i) To examine the effects of smoking on perioperative outcomes and pseudarthrosis rates following anterior cervical corpectomy	(i) Current smoking was significantly associated with higher complication rates (P < 0.001) and longer lengths of stay (P < 0.001). (ii) The complications that were experienced in current smokers were mostly infections (76.5%), and this proportion was greater than that of quitters and nonsmokers (p = 0.013). (iii) The follow-up at 1 year found that current smoking acts as an independent risk factor for pseudarthrosis (P = 0.012).

Leboeuf-Yde et al., 2008 [236]	Secondary analysis for cross-sectional study	(i) N = 412: 169 were nonsmokers, and 243 were current smokers (ii) All subject had same age = 40 years (iii) 199 were males, and 213 were females	(i) To examine whether heavy smoking, overweight, and hard physical work were associated more frequently with vertebral inflammatory process (VIP) than discs generation and low back pain	(i) There was no significant association between smoking and pain or disc generation even though the VIP was reported in higher rate among heavy smokers (26%) compared with the nonsmokers (15%). Also, this association was not found between the hard physical work or overweight with the pain or disc generation. (ii) Compared with subjects who neither smoked heavily nor had a hard physical job, the risks to have VIP were increased in subjects who both were heavy smokers and had a hard physical job (OR = 4.9, 95% CI: 1.6–13.0).

Lee et al., 2015 [237]	Retrospective cohort study	(i) N = 1,038: 705 were nonsmokers, and 333 were smokers (ii) Mean of age was 50 years with a range of 22-89 (iii) 514 were males, and 524 were females	(i) To determine the rate of adjacent segment pathology (ASP) development and to identify the risk factors for reoperation after anterior cervical arthrodesis	(i) The occurrence of secondary surgery on adjacent segments was 2.4% per year (95% CI, 1.9-3.0), and there were 22.2% of patients that need to undergo reoperation for the adjacent segment every 10 years postoperatively (ii) Smoking, female gender, and number of arthrodesis segments (1 or 2 segments had a 1.8 times greater risk than 3 or > segments) were significantly increasing the risk for reoperation.

Macki et al., 2017 [238]	Retrospective cohort study	(i) N = 110: 82 were nonsmokers, and 28 were smokers (ii) Mean of age: 61.1 ± 13.1 for nonsmokers, and 53.9 ± 9.6 for smokers (iii) 47 were males, and 63 were females	(i) To examine the effects of smoking status on rhBMP-2 supplementation in lumbar spinal fusion constructs	(i) After a mean follow-up of 59 months, compared with nonsmokers, smokers had a significantly higher incidence of reoperation for pseudarthrosis, instrumentation failure, or adjacent segment (32% for smokers versus 13.4% for nonsmokers, P = 0.027). (ii) The odds of reoperation among smokers were 4.75-fold higher than that for nonsmokers (95% CI: 1.48–15.24, P = 0.009).

Martin et al., 2016 [239]	Retrospective review of multicenter prospectively collected data	(i) N = 35,477: 27,246 were never-smokers, 563 were former smokers (quit smoking 12 months before the surgery), and 7,669 were current smokers (ii) Mean of age ± SD: 59.70 ± 14.96 for never-smokers, 63.97 ± 12.55 for former smoker, and 50.84 ± 13.12 for current smokers (iii) Percentage of female: 48.4% for never-smokers, 40.57% for former smokers, and 47.16% for current smokers	(i) To determine the impact of smoking or prior smoking cessation on 30 days' morbidity following lumbar spine surgery	(i) Compared with never-smokers, current smokers had a significantly higher risk of total 30-day morbidity (P = 0.04), superficial surgical site infection (P < 0.05), and overall wound complications (P < 0.05). Meanwhile, former smokers show a trend toward having increased risk, but this did not reach significance in any category. (ii) Patients who smoked 1-20 pack-years and >40 pack-years had a significantly greater risk of superficial surgical site infections (P < 0.05 for each)

Sanden et al., 2011 [240]	Prospective cohort study	(i) N = 4,555: 3,797 were nonsmokers, and 758 were current smokers (ii) Mean of age (mean ± SD): for 70.0 ± 8.6 nonsmokers, and 64.6 ± 8.5 for smokers (iii) 2,020 were males, and 2,535 were females	(i) To determine the relation between smoking status and disability after 2 years of surgical treatment for lumbar spinal stenosis	(i) Compared with nonsmokers, smokers need more analgesics (OR = 1.86; 95% CI: 1.55-2.23) and their walking ability was less likely to be improved (OR = 0.65, 95% CI: 0.51-0.82). (ii) Smokers had inferior Quality of Life in most of the scales: Short Form (36) (P < 0.001), EuroQol (P < 0.001), and Oswestry Disability Index (ODI; P < 0.001)

**Section 5: Tendons: Tobacco Smoking and Tendons (6 Articles)**				

Agladioglu et al., 2016 [241]	Case-control	(i) N = 69: 35 smokers, and 34 nonsmokers (ii) Average age in years (Mean ± SD): 35.5±7.8 (iii) 57 Males, and 12 females	(i) Study aims to explore the properties of patellar and Achilles tendons in smoking and nonsmoking healthy adults	(i) Smoking groups had significant thinner patellar and Achilles tendons in the proximal, middle, and distal thirds region of the tendons. Also, smoking groups had significant lower strain ratio measurements in the same regions (P < 0.05). (ii) The thickness of patellar was found to be negatively correlated with smoking amount (all P< 0.05).

Carbone et al., 2012 [242]	Cross-sectional study	(i) N = 408 who underwent arthroscopic repair of cuff tear: 277 nonsmokers,131 smokers (ii) The average of age (Mean ± SD): 59 ± 11.3 with a range of 47-68 (iii) 228 were males, and 108 were females	(i) To assess whether smoking is a risk factor for development of rotator cuff tears and if smoking influence rotator cuff tear size	(i) The rates of type I, type II, type III, and type IV were 23.3%, 52.25%, 18.1%, and 6.1%, respectively. (ii) There were higher percentages of smokers in patients with type II tears compared with type I tear (34.8% versus 23.2%, P = 0.033). (iii) Compared to the patients with type I tear, patients with at least a type II tear had significantly higher total number of cigarettes [F (1,127) = 4.694, P = 0.032]

Justan et al., 2011[243]	Case-control cross-sectional	(i) N = 57 autologous tendon grafts in 48 patients: 28 nonsmokers, and 20 smokers: 9 heavy smokers (≥15 cigarettes/day), 11 light smokers (<15 cigarettes/day)	(i) Aim to study the effect of smoking on postoperative finger range of motion (ROM) in patients with tendon grafts	(i) Smoking groups had significant improvement in their ROM after reconstruction and it is even slightly better in smokers than in nonsmokers

Kukkonen et al., 2014 [244]	Prospective cohort study	(i) N = 564: 450 were nonsmokers, 114 were smokers (ii) Average age in years: 61 ± 9.4 for nonsmokers, and 55±9.1 for smokers (iii) 265 males and 185 females were nonsmokers, and 64 males and 40 females were smokers	(i) To evaluate the effect of smoking on the perioperative outcome of rotator cuff reconstruction	(i) There preoperative Constant score and intraoperative measured tendons tear were not statistically significant between the two groups of smokers and nonsmokers. However, the final postoperative 1-year follow-up for Constant score was significantly lower in smoker group (71 versus 75, P = 0.017). (ii) The pack-years of smoking were not significantly associated with the Constant score (P = 0.815) and the size of the tear (P = 0.786).

Lundgreen et al., 2014 [245]	Case-control cross-sectional	(i) 10 smokers (10 cigarettes or more per day), and 15 nonsmokers with full-thickness tears	(i) Aim to investigate the effect of smoking on the supraspinatus tendon degeneration including cellular alterations, proliferation, and apoptosis of tendon cells	(i) Smokers presented significantly more advanced degenerative changes in the supraspinatus tendons (Bonar score, 13.5 for smokers versus 9 for nonsmokers; P < 0.001), and smoker's tendons showed increased density of apoptotic cells (0.108 versus 0.0107; P = 0.024) accompanied by reduced tenocyte density (P = 0.019) and upregulation of proliferative activity (P < .0001)

Oudelaar et al., 2015 [246]	Retrospective cohort study	(i) N = 431: 319 were nonsmokers, 112 were smokers (ii) Average of age of 51.4 ± 9.9 years. (iii) 154 Males, and 277 females	(i) Aim to study the effect of smoking and morphology on the outcome of needle aspiration of calcific deposits (NACD) for calcific tendinitis of the rotator cuff	(i) Multivariate logistic regression found smoking was significantly associated with failure of NACD (AOR = 1.7, 95%CI: 1.0-2.7, P = 0.04)

**Section 6: Ligaments: Tobacco Smoking and Ligaments (4 Articles)**				

Brophy et al., 2015 [247]	Prospective multicenter cohort study	(i) N = 2,198: 213 smokers, 1947 nonsmokers, and 21 has no data about smoking status. (ii) Average age in years (Mean ± SD): 26.8 ± 11.0	(i) To determine the association between the infection after anterior cruciate ligament (ACL) reconstruction and risk factors such as smoking, diabetes, BMI and graft choice	(i) Seventeen out of 2,198 (0.8%) reported a postoperative infection. (ii) Diabetes and graft choice were found to be a significant risk factor for infection (OR for DM = 18.8; P < 0.001; compared to bone-tendon OR for hamstring autograft = 4.6; P = 0.026, and for hamstring = 4.3; p = 0.047), while the smoking was found to be a nonsignificant factor (OR=2.54; P = 0.167).

Cancienne et al., 2016 [248]	Cohort study	(i) N = 13, 358 with ACL reconstruction: 1,659 tobacco users and 11,699 nontobacco users matched controls. (ii) Range of age = 20-59. (iii) 65% were males, and 35% were female.	(i) To study whether tobacco use is associated with complications after primary ACL reconstruction such as postoperative infection, venous thromboembolism (VTE), arthrofibrosis, and subsequent ACL reconstruction.	(i) Compared to nontobacco users, ACL reconstruction in tobacco use was associated with significantly increased risks of postoperative infection (OR = 2.3; P < 0.0001), VTE (OR = 1.9; P = 0.035), and subsequent ACLS reconstruction (OR = 1.7; P < 0.0001); but it was not found to be significant for the postoperative stiffness (OR = 0.9; P = 0.656).

Kim et al., 2014a [249]	Retrospective cohort study	(i) N = 487 underwent unilateral ACL reconstruction: 165 smokers, and 322 never-smokers. (ii) Average age in years (Mean ± SD): 31.32 ± 91.27 for smokers, and 30.43 ± 11.21 for nonsmokers. (iii) Smokers (22 females, and 143 males); nonsmokers (59 females, and 263 males).	(i) To compare the clinical outcomes of ACL reconstruction between smokers and nonsmokers. (ii) To find an optimal graft in ACL reconstruction with regard to clinical outcomes for smoking patients.	(i) Compared to nonsmokers, the smokers were found to have significant unsatisfactory outcomes regarding the stability (P < 0.001 in side-to-side difference in anterior translation) and functional scores (P < 0.001 in Lysholm Knee Score, IKDC subjective and objective score). (ii) The Achilles tendon-bone allograft showed the worst outcomes, with statistically significant mean differences for smoking patients in the side-to-side difference in anterior translation, Lysholm knee score, and IKDC subjective score

Kim et al., 2014b [250]	Retrospective cohort study	(i) N = 251 with unilateral ACL reconstruction with use of bone autograft: 69 current, 24 former smokers, and 158 nonsmokers. (ii) Average age in years [(Mean, (range)]: 32 (19-66) for current smokers, 34.8 (26-49) for former smokers, and 30.6 (18-60) (for nonsmokers. (iii) Current smokers (7 females, and 62 males); former smokers (3 females and 21 males); nonsmokers (32 females, and 126 males)	(i) To compare the clinical outcomes of ACL reconstruction between current smokers, former smokers, and nonsmokers. (i) To investigate the association between the amount of smoking and outcomes following ACL reconstruction	(i) The three groups differed significantly in terms of stability represented by the postoperative knee translation (P = 0.003) and function represented by Lysholm score (P < 0.001) and IKDC subjective score (P < 0.001) (ii) There was a dose-dependent relationship between pack-years and postoperative anterior translation (P = 0.015) and IKDC objective grade (P = 0.002).

**Section 7: Intrauterine and secondhand smoking effect on musculoskeletal system (8 Articles)**				

Golding et al., 2014 [251]	Secondary data analysis for anthropometrics children measurement (7-17 years) from ALSPAC	(i) N = 8,290 at age 7 to 5,217 at age 17 (attending children for examination)	(i) To investigate the long-term associations between prenatal smoking habits of mothers (M), paternal grandmothers (PGM), and maternal grandmothers (MGM) on the growth of their children	(i) In case of PGM+M-, the girls become taller and both sexes had larger bone and lean mass. However, in case of MGM+M-, the boys had heavier weight with increasing age. (ii) In case of MGM+M+ girls developed lesser height, weight, and fat/lean/bone mass when compared with girls born to cases of MGM-M+

Hagnas et al., 2016 [252]	Prospective cohort study [Northern Finland Birth Cohort 1986]	(i) N = 508 mothers and their offspring: 342 nonsmokers, 95 stopped smoking during pregnancy, and 59 continued smoking during pregnancy (1 or more cigarettes/day)	(i) To investigate the long-term associations between maternal smoking and aerobic fitness in young men aged 19-20 years	(i) Compared to the offspring of nonsmoker mothers during the pregnancy, the offspring for smoker mothers during pregnancy was associated with lower aerobic fitness in Cooper test (2356m; 95%CI: 2265-2446mvs. 2537m, 95%CI: 2499-2574m)

Holmberg et al., 2011 [253]	Cross-sectional	(i) N = 15, 038 underwent bone mineral density (BMD) scan: 5, 829 exposed to home passive smoking in their adulthood (ii) Mean age of 52.7±13.8 [range: 18-95] (iii) 40% were males, and 60% were females	(i) To investigate the association between phalangeal BMD and self-reported passive smoking	(i) Subjects who have been exposed to passive smoking at home as an adult had significantly lower BMD than unexposed subjects (0.343 versus 0.331 g/cm2; P < 0.01), even when adjusted for age, gender, weight, height, and smoking (pack-years; 0.339 versus0.337 g/cm2; P < 0.05)

Jones et al., 2013 [254]	Cohort study	(i) N = 415 (ii) Average of age was 16.3 years (iii) 265 males, and 150 females	(i) To determine if maternal smoking, birth weight, and maternal smoking were associated with BMD and fracture at age of 16 years.	(i) Smoking in utero was not associated with BMD or fracture. (ii) Breastfeeding was associated with 2-3% increase in BMD in all site except radius and was associated with a 1/3 reduction in risk of fracture (iii) Birth weight was associated with hip, radius, and total body BMD, but such association did not stand after the multivariate analysis. Furthermore, birthweight was not associated with the fracture

Kim et al., 2013 [255]	Cross-sectional study	(i) N = 925 never smokes: 212 with secondhand smoke (SHS), and 713 without SHS (ii) Mean age of 51.4 ± 9.9 years (iii) Mean of age (mean ± SD): 64.6 ± 7.1 for subject with SHS, and 66.3 ± 7.8 for subject without SHS	(i) To assess the association between SHS and postmenopausal osteoporosis	(i) Compared to participants not exposed to SHS, participants who are actively exposed to SHS from family members had higher adjusted OR for femoral neck osteoporosis (OR: 3.68; 95%CI: 1.23-10.92) (ii) Compared with the nonexposed group, the group who lived with cohabitant smokers had increased risk for lumbar and femoral osteoporosis regardless of the number of cigarettes consumed by their cohabitant

Macdonald-Wallis et al., 2011 [256]	Prospective cohort study from ALSPAC	(i) N = 7,121 children: 6,101 sets of parents: 3,576 both parents nonsmoked, 369 only mother smoked, 1,313 only father smoked, and 843 both parents smoked	(i) Study aims to investigate an intrauterine influence of maternal and paternal smoking during pregnancy on offspring bone mass at mean age 9.9 years	(i) Both paternal and maternal smoking were associated with increased total body less head (TBLH) and spine BMC, bone area (BA), and BMD in girls but not boys

Martinez-Mesa et al., 2014 [257]	Prospective cohort study (1993 Pelotas Birth Cohort)	(i) N = 3,075 adolescents (ii) 1,512 were Males, and 1,563 were females	(i) To evaluate the associations between maternal smoking during pregnancy with offspring bone health at age of 18 years	(i) Study found that there was an inverse association between cigarette smoking during pregnancy and BMC [-4.20 g per 1 cigarette in males (95%CI: -8.37 to -0.05), but not in females [-2.22 g per 1 cigarette (95%CI: -5.49 to 1.04)]; weaker inverse associations were observed for BMD

Zadzinska et al., 2016 [258]	Cross-sectional study	(i) N = 978 (ii) Range of age: 7-10 years (iii) 348 were males, and 630 were females	(i) To assess the effect of maternal, paternal, and parental smoking during pregnancy on relative leg length in 7-10-year-old children.	(i) Neither mother nor father smoking during pregnancy showed a significant effect on relative leg length of the offspring. However, both parents' smoking showed a significant effect on relative leg length of offspring (ii) The parent who smoke has a greater risk to have offspring with shorter leg length (OR = 2.7) as compared with parents who do not smoke

**Note:** Type of sample was mentioned when it is only random; otherwise the sample was nonrandom sample. Race/ethnicity was mentioned if it were given in the article.
